# *Verticillium dahliae-Arabidopsis* Interaction Causes Changes in Gene Expression Profiles and Jasmonate Levels on Different Time Scales

**DOI:** 10.3389/fmicb.2018.00217

**Published:** 2018-02-13

**Authors:** Sandra S. Scholz, Wolfgang Schmidt-Heck, Reinhard Guthke, Alexandra C. U. Furch, Michael Reichelt, Jonathan Gershenzon, Ralf Oelmüller

**Affiliations:** ^1^Department of Plant Physiology, Matthias Schleiden Institute of Genetics, Bioinformatics and Molecular Botany, Friedrich-Schiller-University Jena, Jena, Germany; ^2^Systems Biology and Bioinformatics Group, Leibniz Institute for Natural Product Research and Infection Biology—Hans-Knöll-Institute, Jena, Germany; ^3^Department of Biochemistry, Max-Planck Institute for Chemical Ecology, Jena, Germany

**Keywords:** *Arabidopsis*, calcium, JA, defense, *Verticillium dahliae*

## Abstract

*Verticillium dahliae* is a soil-borne vascular pathogen that causes severe wilt symptoms in a wide range of plants. Co-culture of the fungus with *Arabidopsis* roots for 24 h induces many changes in the gene expression profiles of both partners, even before defense-related phytohormone levels are induced in the plant. Both partners reprogram sugar and amino acid metabolism, activate genes for signal perception and transduction, and induce defense- and stress-responsive genes. Furthermore, analysis of *Arabidopsis* expression profiles suggests a redirection from growth to defense. After 3 weeks, severe disease symptoms can be detected for wild-type plants while mutants impaired in jasmonate synthesis and perception perform much better. Thus, plant jasmonates have an important influence on the interaction, which is already visible at the mRNA level before hormone changes occur. The plant and fungal genes that rapidly respond to the presence of the partner might be crucial for early recognition steps and the future development of the interaction. Thus they are potential targets for the control of *V. dahliae*-induced wilt diseases.

## Introduction

Vascular wilts caused by members of the genus *Verticillium* are among the most devastating fungal diseases worldwide, and these soil-borne ascomycete fungi attack a large variety of plant hosts in many parts of the world (Decetelaere et al., 2017) which leads to massive yield losses (Pegg and Brady, 2002). Among the 10 species within the *Verticillium* genus, *Verticillium dahliae* has the broadest host range with the ability to infect >200 plant species worldwide (Agrios, 1997; Inderbitzin et al., 2011; Inderbitzin and Subbarao, 2014). *Verticillium* species produce microsclerotia, which can survive in soil or dead plant material for more than 10 years, but they also form resting mycelia which survive in dead plant material. Newly growing hyphae rapidly penetrate the roots of their hosts, reach the vascular tissue and ultimately propagate in the xylem (Puhalla and Bell, 1981; Schnathorst, 1981). Initially, these hemibiotrophic fungi show biotrophic behavior that does not lead to severe reductions in plant performance. However, at later stages, they shift to a necrotrophic interaction characterized by the reprogramming of phytohormone metabolism (Veronese et al., 2003; Thaler et al., 2004; Tjamos et al., 2005), synthesis of hydrogen peroxide (H_2_O_2_) and nitric oxide (NO, Yao et al., 2011, 2012), and defense gene activation which ultimately induces host cell death (Reusche et al., 2012; Zhang et al., 2016). A typical symptom of these pathogens is wilting, which occurs as a consequence of impaired vascular transportation (Reusche et al., 2012).

*Arabidopsis* is an ideal model plant to study the *Verticilllium* infection process at the molecular level. Sun et al. (2014) showed that jasmonate phytohormones are highly induced upon *V. dahliae* infection, including the jasmonic acid (JA) pre-cursor *cis*-(+)-12-oxo-phytodienoic acid (*cis*-OPDA) and the jasmonic acid-isoleucine conjugate (JA-Ile). JA-Ile, synthesized by the enzyme JASMONATE RESISTANT 1 (JAR1), is the active signaling compound that binds to the JA receptor CORONATINE INSENSITIVE 1 (COI1) to initiate the downstream signaling cascade (Xie et al., 1998; Staswick and Tiryaki, 2004; Fonseca et al., 2009). Hydroxylation of JA-Ile by a P450 enzyme leads to the deactivation of the molecule (Koo et al., 2011). Previous observations on JA-deficient *def1* tomato plants showed decreased fitness after *V. dahliae* treatment (Thaler et al., 2004), while external application of methyl JA (MeJA) to cotton and tomato plants partially blocked disease development due to the reduced growth of the fungus (Li et al., 1996). Recent RNA-seq analysis in cotton showed that in addition to the biosynthesis of JA, also other components of the JA signaling cascade are targets of *Verticillium*. For instance, the gene of a repressor protein of COI1, *GhJAZ10*, was significantly upregulated in a *V. dahliae*-resistant cotton cultivar (Chini et al., 2007; Thines et al., 2007; Zhang W. W. et al., 2017). On the other hand, *Verticillium* itself needs an activated COI1 signaling pathway in the plant to cross the root-shoot barrier and to induce disease symptoms in the leaf. Consequently, *V. dahliae*-infected *coi1* mutant plants showed less severe wilt symptoms (Ralhan et al., 2012).

Besides jasmonates, *Verticillium*-infested plants also accumulate other phytohormones like salicylic acid (SA) and ethylene (ET), (Fradin and Thomma, 2006; Ratzinger et al., 2009; Sun et al., 2014). SA plays an important role in plant defense against pathogens by activation of systemic acquired resistance (SAR) (Metraux, 2001). In *Brassica napus* the concentration of SA increased after infection with *Verticillium longisporum* in the xylem sap of the plants (Ratzinger et al., 2009). Also in *Arabidopsis*, a significant increase of SA could be detected in *V. dahliae*-infested plants (Sun et al., 2014). Exogenous application of SA protected cotton callus cells against a *V. dahliae (VD)*-toxin preparation by increase of chitinase and β-1,3-glucanase activities (Li et al., 2003; Zhen and Li, 2004). Interestingly, several *Arabidopsis* genotypes affected in different steps of SA signaling did not show altered resistance against *V. dahliae* infection (Veronese et al., 2003).

The role of ET in pathogen responses is still controversial although it was shown that ET can increase resistance and control symptom expression in some hosts. In soybean, tobacco and *Aarabidopsis*, ET is involved in host resistance against particular classes of pathogens (Knoester et al., 1998; Hoffman et al., 1999; Thomma et al., 1999), while a tomato mutant impaired in ET perception exhibited a significant reduction in disease symptoms after inoculations with different bacterial and fungal pathogens (Lund et al., 1998). Similarly, a tomato mutant with decreased ET biosynthesis showed significantly reduced symptom development (Robinson et al., 2001). The involvement of ET in response to *Verticillium* infection and *VD*-toxins has been shown repeatedly (Pegg and Cronshaw, 1976; Mansoori and Smith, 2005; Sun et al., 2014). Also the ET-resistant *Arabidopsis* mutant *etr1-1* was found to display less chlorosis upon *Verticillium* inoculation (Veronese et al., 2003; Tjamos et al., 2005; Pantelides et al., 2010).

A growing number of other chemical compounds of both plant and fungal origin have been identified that participate in disease development of infected host plants. For *Verticillium*, a range of peptides, hormones like cytokinins, and other metabolites were shown to confer partial resistance to infection (Reusche et al., 2013; Bu et al., 2014; Gaspar et al., 2014; Roos et al., 2014). In addition, the *Ve* gene provides resistance against isolates of *V. dahliae* in tomato and *Arabidopsis* (Kawchuk et al., 2001; Fradin et al., 2009, 2011) and the *VET1* gene was able to convey increased tolerance with milder chlorosis symptoms (Veronese et al., 2003).

Due to the vascular location of the growing fungus, *Verticillium* wilt of agricultural plants is difficult to control by chemicals or molecular tools. Even after removal of infected plants from agricultural fields, the resting structures (microsclerotia) remain in soil making them a hazard for future plantings (Fradin and Thomma, 2006). Biocontrol studies with bacterial and fungal isolates like *Pseudomonas putida* B E2, *Pseudomonas chlororaphis* K15, *Serratia plymuthica* R12 or *Paenibacillus alvei* K165 on *Solanaceae, Malvaceae*, and *Brassicaceae* have been shown to be an alternative strategy to restrict *Verticillium*-induced wilts (Berg et al., 2001; Tjamos et al., 2005; Li et al., 2012). Sun et al. (2014) showed that *Piriformospora indica*, a beneficial endophytic fungus of Sebacinales which colonizes the roots of many plant species, is an efficient biocontrol agent that restricts *V. dahliae* growth in the model plant *Arabidopsis thaliana*.

*V. dahliae* rapidly and efficiently colonizes *Arabidopsis* roots within 24 h post germination. In order to identify plant and fungal genes involved in these early recognition processes we performed an RNA-seq analysis under standardized co-cultivation conditions. We found a massive reprogramming of both the plant and fungal expression profiles that occurred before pathogen induced, defense-related phytohormone levels changed in the host. This alteration in expression profiles clearly indicates that both plant and pathogen respond very rapidly to the presence of the other.

## Materials and methods

### Growth conditions of seedlings

*A. thaliana* wild-type (ecotype Columbia-0) seeds, and seeds of the *jar1, coi1-16*, and *cyp94B3* mutants (kindly provided by Axel Mithöfer, MPI-CE, Jena) were surface-sterilized and placed on Petri dishes with MS media supplemented with 0.3% gelrite (Murashige and Skoog, 1962). After cold treatment at 4°C for 48 h, plates were incubated vertically for 9 or 14 days at 22°C under long day conditions (16 h light/8 h dark; 80 μmol m^−2^ s^−1^, for experimental setup, cf. Figure S1).

### Growth conditions of fungi and preparation of spore solutions

*V. dahliae* wild-type (FSU-343, Jena Microbial Resource Center, Germany) and a GFP-labeled strain (IPP0860, kindly provided by Prof. Tiedemann, University of Göttingen) were grown for 1–2 weeks on Potato-Dextrose-Agar (PDA) medium (Bains and Tewari, 1987) at 23°C in the dark. To obtain high spore production, the still white mycelia (with low amount of microsclerotia) were transferred to liquid KM medium (Hill and Kaefer, 2001) and incubated for 4–5 days at room temperature (RT) in the dark and 110 rpm. The cultures were filtered through two layers of a nylon membrane (75 μm pore size), pelleted and washed with water. The spore concentration was determined with a hemocytometer and adjusted to 5 × 10^6^ per ml.

### Co-cultivation assays

For short time co-cultivation assays, 9 day-old *A. thaliana* seedlings of equal sizes were used. Co-cultivation of *A. thaliana* and the fungus was performed under *in vitro* culture conditions on a nylon membrane on PNM medium (Johnson et al., 2011). Two days prior to use, 100 μl of the generated spore solutions (in water) or an equal amount of water were plated on six sterile membrane stripes (4 × 1 cm) placed on PDA plates and incubated at room temperature (Figure S1). For the co-cultivation the membrane stripes with fungus or water were placed on the PNM plates together with two plants per membrane. The plants were placed in the way that the roots were in contact with the fungus while the leaves were not (Figure S1). Plates were sealed with 3M™ Micropore tape and incubated for 24 h at 80 μmol m^−2^ s^−1^ with light from one side (leaves directed to light). Roots and leaves were harvested separately for further analysis. All experiments were performed three times independently (≈120 seedlings per replicate).

For long term co-cultivation [10 and 20 *days post infection* (*dpi*)], 14 day-old *A. thaliana* seedlings of equal size were used. Plants were grown and infected as described above and transferred to Magenta boxes (Sigma-Aldrich, Germany) after 24 h. One plant was added per box which contained 30 g sterile vermiculite mixed with 100 ml liquid PNM medium. The boxes were incubated at 23°C under short day conditions (9 h light/15 h dark; 80 μmol m^−2^ s^−1^). After 10 or 20 *dpi*, plants were visually examined and photographed. The performance of the plants [*n* = 6 (control) and 9 (*VD*-infected)] was quantified based on the efficiency of the photosynthetic electron transfer measured with the Fluorcam as described before (Matsuo et al., 2015).

### RNA isolation and RNA-seq

RNA of both plant and fungus was isolated from 100 mg root material (≈ 120 seedlings per replicate, *n* = 3). The frozen roots were finely ground with mortar and pistil and weighed. The RNA from the samples was extracted with peqGOLD TriFast™ FL (VWR, Darmstadt, Germany) according to the manufacturer's protocol. The RNA was further processed by use of the PureLink™ RNA Mini Kit (Thermo Fisher Scientific, Dreieich, Germany) with on-column DNAse treatment. RNA was dissolved in water and checked for quality. The isolated RNA was shipped to GeneCore (Heidelberg, Germany) where library construction was performed using the mRNA sequencing Sample Preparation Guide (Illumina, Cat#RS-930-1001), followed by a validation of the library and RNA sequencing (Hi Seq 2000, single-end 75 bp, ~50 Mill. reads/sample). The removal of low quality reads and Illumina adapters was performed using Trimmomatic (Bolger et al., 2014). The remaining reads were then aligned to the *A. thaliana* (TAIR10.33) and *V. dahliae* (ASM15067v2.37) reference genomes using the RNA-seq aligner STAR (Dobin et al., 2013). Differential gene expression analysis was performed using DESeq2 (Love et al., 2014) with the raw counts obtained from FeatureCounts (Liao et al., 2014). Differentially Expressed Genes (DEGs) of both interaction partners were analyzed [FoldChange ≥ 2 and *p*-Value (FDR) ≤ 0.05]. The function of the DEGs was analyzed with the TAIR (www.arabidopsis.org) and the Fungi Ensembl (http://fungi.ensembl.org/Verticillium_dahliae) databases. Pathway analysis for the plant and the fungus was executed with the KEGG (Kyoto Encyclopedia of Genes and Genomes) Mapper tool (http://www.genome.jp/kegg/tool/map_pathway2.html; RRID:SCR_012773).

### Analysis of gene expression and fungal colonization

The RNA (*n* = 3) was isolated as described above. One μg of RNA was transcribed to cDNA using the Omniscript RT Kit (Qiagen, Hilden, Germany). Fifty nanograms of synthesized cDNA was used as template for RT-qPCR in a CFX Connect™ Real-Time PCR Detection System (Bio-Rad, Munich, Germany) with the Brilliant II SYBR® Mastermix (Agilent, Böblingen, Germany). The mRNA levels for each cDNA probe were normalized with respect to the *RPS18B* (plant) or *VD_Actin2* (fungus, e.g., Klimes and Dobinson, 2006; Yang et al., 2013) mRNA levels. The normalized fold expression of GOIs was calculated according to ΔΔCP (Pfaffl, 2001). The primer pairs are given in Table S5. To analyze fungal colonization of different mutant plants, normalized fungal housekeeping gene expression was compared to that in WT plants.

### Confocal microscopy

Samples for confocal laser scanning microscopy were prepared according to the method for the short time co-cultivation described above. The GFP-labeled *V. dahliae* strain on *Arabidopsis* seedlings was imaged using a LSM 880 (Zeiss Microscopy GmbH, Jena, Germany) with the 488 nm laser line of an argon multiline laser (11.5 mW). Images were taken with a 40x objective (Plan-Apochromat 40x/0.8). Lambda stacks were created using the 32 channel GaAsP detector followed by Linear Unmixing with ZEN software (Zeiss, Jena, Germany). Z-stacks were taken from specific areas of the sample and Maximum Intensity Projections were produced with ZEN software. Cross-sections of the roots with a width of 14 μm were done with a Microm HM560 Cryostar (Southeast Pathology Instrument Service, Charleston, USA).

### Quantification of phytohormones

Phytohormones were extracted from the green parts of co-cultivated seedlings (1 sample = seedlings from 1 plate, ≈ 50–100 mg, *n* = 10). Frozen samples were homogenized for 30 s at 1000 rpm in a 2010 Geno/Grinder® (Spex SamplePrep, Stanmore, UK) and mixed with 1 ml methanol containing 40 ng/ml of D_6_-JA, D_6_-ABA, D_4_-SA, and 8 ng/ml of D_6_-JA-Ile (Scholz et al., 2017). All samples were shaken for 30 min at 4°C and centrifuged at 13,000 rpm for 20 min at 4°C. The supernatants were collected and the sample re-extracted with 500 μl methanol. The combined supernatants were evaporated to dryness at 30°C using a vacuum concentrator. Residues were re-suspended in 200 μl methanol and centrifuged at 13,000 rpm for 10 min. The supernatants were collected and measured with the API 5000 LC-MS/MS system (Applied Biosystems, Framingham, USA) as previously described (Vadassery et al., 2012). Since it was observed that both the D_6_-labeled JA and JA-Ile contained 40% of the corresponding D_5_-labeled compounds, both peaks were combined for analysis.

### Statistics

Experiments were repeated three times to ensure reproducibility and 120–150 seedlings were used in each treatment for each mutant. Data of all independent experiments were pooled and analyzed. For comparison of two groups, the Mann Whitney U-test was applied. For statistical analyses of multiple groups, one-way analysis of variance (one-way ANOVA) was used as indicated in the figure legends. Different letters indicate significant differences between treatments. SigmaPlot13 and Origin Pro were used for data analysis and graph composition.

## Results

### Co-cultivation of *Arabidopsis* seedlings with *V. dahliae* for 24 H

**Figure 1:**
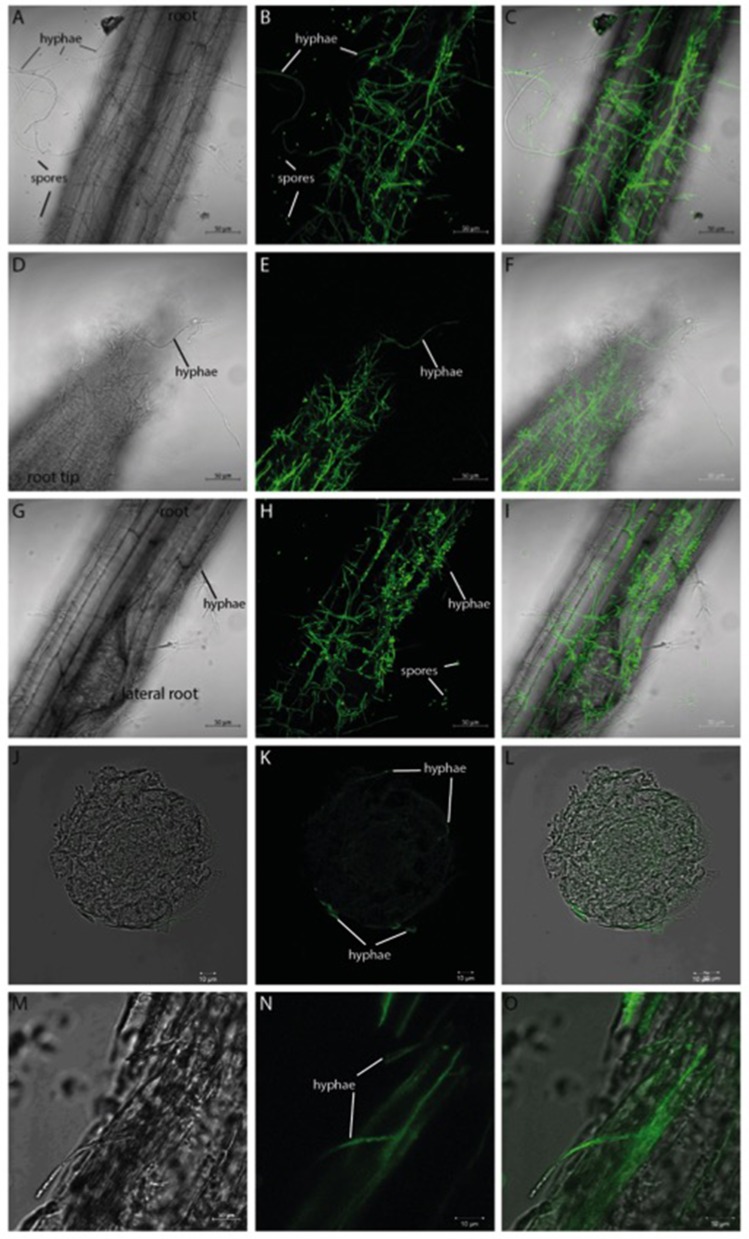
GFP-labeled *Verticillium dahliae* colonizing roots of 10 day-old seedlings of *Arabidopsis thaliana* after 24 h co-culture. Shown are pictures (obtained from confocal microscopy) of the colonized *Arabidopsis* root surface **(A–C)**, root tip **(D–F)**, area around lateral roots **(G–I)** as well as a cross-section **(J–L)** and longitudinal section **(M–O)**. A bright field image (left), the GFP image (middle), and the overlay of both (right) are shown for each analyzed area. Both the hyphae and the spores of *V. dahliae* show a GFP fluorescence.

*Verticillium* species are considered as hemibiotrophs, where a biotrophic phase—within the root xylem without a visible disease phenotype—is followed by a necrotrophic phase in the aerial parts of the plant (Reusche et al., 2012). We focused on the very early phase of infection, the pre-vascular growth phase, and analyzed the plant and fungal expression profiles during the first 24 h of fungal root colonization in *Arabidopsis*. By developing a stable co-cultivation method (scheme in Figure S1), a reproducible colonization of the seedlings by the fungus was achieved. Confocal microscopic pictures taken 24 h after co-culture demonstrate that *Arabidopsis* roots are already heavily colonized by the GFP-labeled *V. dahliae* (Figure 1). The hyphae of the fungus form a net over the root surface and root tip while the first spores can be observed at the site of lateral root formation (Figures 1G–I). In cross-sections of the colonized root (Figures 1J–O) it can be observed that the fungus penetrates the root surface, but did not yet invade the vascular tissue after 24 h of co-culture.

### Dual-RNA-seq of *Arabidopsis-Verticillium* co-culture reveals high number of differentially-expressed genes (DEGs)

Recent studies of plant-*Verticillium* co-cultures focus on analysis of the plant transcriptome by RNA-seq and indicate major changes in nearly 19% of pathways in early phases of infection (1 or 4 *dpi*, e.g., Faino et al., 2012; Zhang W. W. et al., 2017). The RNA-seq data generated in this study 24 h after co-cultivation was analyzed for changes in both the plant and fungal transcriptomes and revealed a total number of 4432 DEGs for both organisms (Table 1). Compared to *Arabidopsis* seedlings grown alone, co-cultivation with *Verticillium* results in 1143 DEGs, 903 of them are significantly up-regulated and 240 down-regulated (Tables S1, S2). For the fungus, 3289 DEGs were detected with 1695 significantly up-regulated (Table S3) and 1594 significantly down-regulated genes (Table S4). This suggests that the fungal expression profile responds more strongly to the presence of a host than the plant genome to a pathogen. The results for selected genes were verified by RT-qPCR (**Figures 7**, **8**, WT).

**Table 1:** Overview of differentially regulated genes in *A. thaliana* (ATH) and *V. dahliae* (VDA) co-culture compared to organisms grown separately.

	**Up**	**Down**
ATH grown with VDA vs. ATH alone	903	240
VDA grown with ATH vs. VDA alone	1695	1594

### Multiple *Arabidopsis* pathways are affected by *V. dahliae* infection

**Figure 2:**
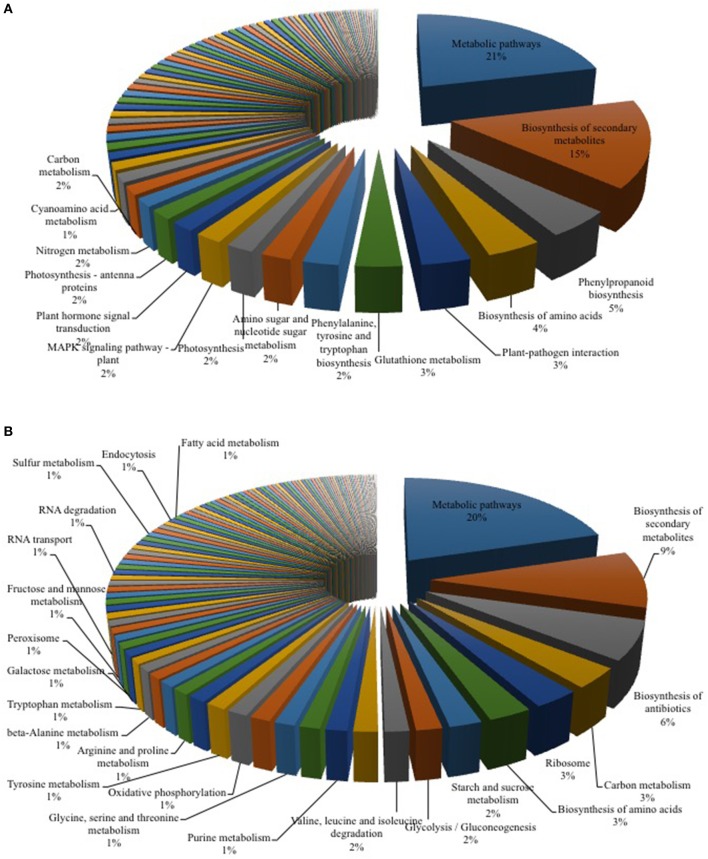
KEGG Mapper analysis of RNA-seq data for *Arabidopsis-Verticillium* co-culture. Shown are the mapped pathways (*n* = 3) for differentially expressed genes in *Arabidopsis* alone vs. co-culture **(A)** and *Verticillium* alone vs. co-culture **(B)**. A full list of the other regulated pathways is shown in Tables 2, 3.

Previous studies during longer co-cultivation times have shown that infection of *Arabidopsis* plants with *Verticillium* species results in disruption of water transport and massive accumulation of phytohormones like jasmonates (JAs), ethylene (ET), salicylic acid (SA), or abscisic acid (ABA), as well as stomata closure (Pegg and Brady, 2002; Fradin and Thomma, 2006; Klosterman et al., 2009; Ralhan et al., 2012; Sun et al., 2014). To identify early targets of the fungus in *Arabidopsis* and to analyze and classify the plant DEGs, we chose the 24 h co-cultivation time point and mapped the identified plant DEGs to distinct pathways in the KEGG database (Kanehisa, 1997). In total, 78 pathways were affected by the fungus (Table 2 and Figure 2A). Metabolic pathways involved in carbon and amino acid metabolism seemed to be the major targets, such as *SWEET* genes and sugar efflux transporters, which often respond to pathogens and symbionts for nutritional gain (Chen et al., 2010). In our study, *SWEET11*, −*3*, and −*12* were down-regulated. Genes involved in plant development were also affected. So was ROOT CAP POLYGALACTURONASE28, a crucial player in root tip growth, which was also down-regulated at the mRNA level (Kamiya et al., 2016). Defense-related genes, genes involved in the synthesis and propagation of defense signals like JA, SA, NO, and reactive oxygen species (ROS), membrane-associated receptor kinases are up-regulated while pathways involved in or related to photosynthesis, carotenoid and flavonoid biosynthesis are down-regulated. We observed the activation of genes for various defense-related WRKY transcription factors (WRKY18, −28, −30, −33, −41, −45, −53, −55, −71). It has been shown that WRKY71 promotes shoot branching, acceleration of flowering and cell death (cf. references in TAIR, www.Arabidopsis.org); WRKY33 is a target of *Botrytis* to repress defense in *Arabidopsis* (Liu et al., 2017), WRKY53 is involved in disease resistance against *Verticillium longisporium* (Reusche et al., 2013), and WRKY30 confers general abiotic stress response to the plant (Scarpeci et al., 2013). Upregulation of these genes suggest that the plant reprograms its metabolism for defense. Also candidate genes for the perception of pathogen-associated molecular patterns, and enzymes as well as signaling components which might lead to the activation of defense compounds in the host were upregulated in the presence of the fungus. This includes six genes for receptor-like protein kinases (RLP7, −15, −19, −20, −35, −38) and seven genes for proteins with TIR-NBS-LRR domains involved in disease resistance responses (At1g57630; At1g66090; At5g45240; At5g41750; At1g63750; At1g72900, At1g72920). For the RLP genes, no clear function has been described yet. The mRNAs for the Toll and Interleukin-1 Receptor (TIR)-Nucleotide-Binding Site (NBS)-Leucine-Rich Repeat (LRR) proteins (TIR-NBS-LRR), At1g66090 and At1g72920, have been shown to travel to distant tissue upon stress and thus might be involved in systemic signal propagation (Thieme et al., 2015). Furthermore, numerous genes for cytochrome P450 enzymes responded to the fungus. Of these, 17 are up-regulated and 11 have a described role in plant defense (cf. TAIR). Two of the three down-regulated genes (CYP87A2 and CYP705A12) are involved in cytokinin signaling (cf. TAIR). Many of the *cyp* mRNAs are also known to be mobile within the plant. Finally, defense gene activation is often mediated via Ca^2+^ signaling and Ca^2+^-binding proteins. The majority of genes that code for proteins with Ca^2+^-related functions are involved in signaling, ion uptake and distribution. Many of these are also up-regulated in *Arabidopsis* upon *V. dahliae* infection. At an early phase of interaction with the pathogen, the plant appears to shift its resources from primary metabolism to defense processes (cf. Discussion).

**Table 2:** KEGG pathway classification of genes differentially expressed in *Arabidopsis* alone vs. co-culture with *V. dahliae*.

**KEGG Pathway**	**Number**	**Percent**	**Pathway ID**
Metabolic pathways	113	21.3	ath01100
Biosynthesis of secondary metabolites	81	15.3	ath01110
Phenylpropanoid biosynthesis	25	4.7	ath00940
Biosynthesis of amino acids	20	3.8	ath01230
Plant-pathogen interaction	18	3.4	ath04626
Glutathione metabolism	17	3.2	ath00480
Phenylalanine. tyrosine and tryptophan biosynthesis	13	2.4	ath00400
Amino sugar and nucleotide sugar metabolism	11	2.1	ath00520
Photosynthesis	10	1.9	ath00195
MAPK signaling pathway—plant	10	1.9	ath04016
Plant hormone signal transduction	9	1.7	ath00380
Photosynthesis—antenna proteins	9	1.7	ath04075
Nitrogen metabolism	8	1.5	ath01200
Carbon metabolism	8	1.5	ath00460
Tryptophan metabolism	8	1.5	ath00910
Cyanoamino acid metabolism	7	1.3	ath00966
Starch and sucrose metabolism	6	1.1	ath00500
Glucosinolate biosynthesis	6	1.1	ath00966
Glycine. serine and threonine metabolism	6	1.1	ath00260
Pentose and glucuronate interconversions	5	0.9	ath00040
2-Oxocarboxylic acid metabolism	5	0.9	ath01210
Flavonoid biosynthesis	5	0.9	ath00941
Glycolysis/Gluconeogenesis	5	0.9	ath00010
Carotenoid biosynthesis	5	0.9	ath00906
Cysteine and methionine metabolism	5	0.9	ath00270
Protein processing in endoplasmic reticulum	5	0.9	ath04141
Phenylalanine metabolism	4	0.8	ath00360
Tropane. piperidine and pyridine alkaloid biosynthesis	4	0.8	ath00960
Sulfur metabolism	4	0.8	ath00920
Purine metabolism	4	0.8	ath00230
Valine. leucine and isoleucine degradation	4	0.8	ath00280
Sesquiterpenoid and triterpenoid biosynthesis	4	0.8	ath00909
Beta-Alanine metabolism	3	0.6	ath00410
SNARE interactions in vesicular transport	3	0.6	ath04130
Fatty acid degradation	3	0.6	ath00071
Fatty acid metabolism	3	0.6	ath01212
Pentose phosphate pathway	3	0.6	ath00030
Ubiquinone and other terpenoid-quinone biosynthesis	3	0.6	ath00130
Propanoate metabolism	3	0.6	ath00640
Pyruvate metabolism	3	0.6	ath00620
Taurine and hypotaurine metabolism	3	0.6	ath00430
Ascorbate and aldarate metabolism	3	0.6	ath00053
Alpha-Linolenic acid metabolism	3	0.6	ath00592
Fatty acid biosynthesis	3	0.6	ath00061
Carbon fixation in photosynthetic organisms	3	0.6	ath00710
Steroid biosynthesis	3	0.6	ath00100
Galactose metabolism	3	0.6	ath00052
Tyrosine metabolism	3	0.6	ath00350
Inositol phosphate metabolism	2	0.4	ath00562
Selenocompound metabolism	2	0.4	ath00450
Monobactam biosynthesis	2	0.4	ath00261
Isoquinoline alkaloid biosynthesis	2	0.4	ath00950
Protein export	2	0.4	ath03060
Linoleic acid metabolism	2	0.4	ath00591
RNA degradation	2	0.4	ath03018
Peroxisome	2	0.4	ath04146
Fructose and mannose metabolism	2	0.4	ath00051
Arachidonic acid metabolism	1	0.2	ath00590
Citrate cycle (TCA cycle)	1	0.2	ath00020
Alanine. aspartate and glutamate metabolism	1	0.2	ath00250
Transporters	1	0.2	ath02010
Riboflavin metabolism	1	0.2	ath00740
Lysine degradation	1	0.2	ath00310
Flavone and flavonol biosynthesis	1	0.2	ath00944
Glyoxylate and dicarboxylate metabolism	1	0.2	ath00630
Zeatin biosynthesis	1	0.2	ath00908
Vitamin B6 metabolism	1	0.2	ath00750
Valine. leucine and isoleucine biosynthesis	1	0.2	ath00290
Terpenoid backbone biosynthesis	1	0.2	ath00900
Biosynthesis of unsaturated fatty acids	1	0.2	ath01040
Circadian rhythm—plant	1	0.2	ath04712
Stilbenoid. diarylheptanoid and gingerol biosynthesis	1	0.2	ath00945
Pantothenate and CoA biosynthesis	1	0.2	ath00770
Butanoate metabolism	1	0.2	ath00650
Indole alkaloid biosynthesis	1	0.2	ath00901
Phagosome	1	0.2	ath04145
Glycerophospholipid metabolism	1	0.2	ath00564
Lysine biosynthesis	1	0.2	ath00310

Interestingly, two genes for SNARE proteins (soluble N-ethylmaleimide-sensitive-factor attachment receptor, At5g39630, and At1g16225) also responded to the fungus. SNARE proteins are involved in vesicle trafficking and membrane fusion and deliver defense products to infection sites during exocytosis-associated immune responses (Wang et al., 2017, and references cited therein). Stimulation of *EXO70H4* and *EXO70A3* for EXOCYST subunits is consistent with the stimulation of exocytosis-mediated callose deposition and cell wall maturation by the fungus (Li et al., 2010; Kulich et al., 2015).

### *V. dahliae* pathways respond to the interaction with *Arabidopsis*

Many genes annotated in the 117 fungal pathways that were affected by co-cultivation with *Arabidopsis* (Table 3 and Figure 2B), are still uncharacterized or with unknown function, which limits our analysis on the fungal side. However, like on the plant side, the majority of the affected pathways involved in primary metabolism, e.g., genes for the amino acid metabolism, were highly down-regulated, while those for sugar metabolism and sugar transport processes were up-regulated. This suggests that the fungus starts quite early to reprogram its primary metabolism and adapts amino acid and sugar metabolism to being inside a host. mRNA for several enzymes employed in degradation of the plant cell wall, such as the cell wall glycosyl-hydrolase *YteR* (Moore et al., 2016), were up-regulated as shown previously. Genes involved in processes such as oxidative phosphorylation, ribosome formation, RNA transport and degradation or proteasome functions were preferentially down-regulated in the co-cultivated fungus. Closer inspection of the data supports the idea that the fungus down-regulates genes for essential processes (primary sucrose, N and P metabolism, ion homeostasis, redox processes, defense, and secondary metabolites) perhaps because of its increasing reliance on the plant. Reduced defense gene activation may indicate that the fungus prevents the synthesis of compounds that restrict its growth and propagation in the host.

**Table 3:** KEGG pathway classification of genes differentially expressed in *Verticillium* alone vs. co-culture.

**KEGG Pathway**	**Number**	**Percent**	**Pathway ID**
Metabolic pathways	344	20.4	vda01100
Biosynthesis of secondary metabolites	151	9.0	vda01110
Biosynthesis of antibiotics	104	6.2	vda01130
Carbon metabolism	55	3.3	vda01200
Ribosome	50	3.0	vda03010
Biosynthesis of amino acids	47	2.8	vda01230
Starch and sucrose metabolism	35	2.1	vda00500
Glycolysis/Gluconeogenesis	28	1.7	vda00010
Valine. leucine and isoleucine degradation	26	1.5	vda00280
Pentose and glucuronate interconversions	26	1.5	vda00040
Purine metabolism	23	1.4	vda00230
Amino sugar and nucleotide sugar metabolism	22	1.3	vda00520
Glycine. serine and threonine metabolism	22	1.3	vda00260
Pyruvate metabolism	22	1.3	vda00620
Oxidative phosphorylation	22	1.3	vda00190
Tyrosine metabolism	22	1.3	vda00350
Cysteine and methionine metabolism	20	1.2	vda00270
Arginine and proline metabolism	19	1.1	vda00330
Glycerolipid metabolism	18	1.1	vda00561
Propanoate metabolism	17	1.0	vda00640
Beta-Alanine metabolism	17	1.0	vda00410
Tryptophan metabolism	16	0.9	vda00380
Galactose metabolism	16	0.9	vda00052
Peroxisome	16	0.9	vda04146
Methane metabolism	15	0.9	vda00680
Fructose and mannose metabolism	15	0.9	vda00051
Butanoate metabolism	15	0.9	vda00650
Cyanoamino acid metabolism	15	0.9	vda00460
RNA transport	14	0.8	vda03013
2-Oxocarboxylic acid metabolism	14	0.8	vda01210
Histidine metabolism	13	0.8	vda00340
Protein processing in endoplasmic reticulum	13	0.8	vda04141
Alanine. aspartate and glutamate metabolism	13	0.8	vda00250
Ribosome biogenesis in eukaryotes	12	0.7	vda03008
RNA degradation	12	0.7	vda03018
Steroid biosynthesis	12	0.7	vda00100
Phenylalanine metabolism	12	0.7	vda00360
Lysine degradation	12	0.7	vda00310
Pentose phosphate pathway	12	0.7	vda00030
Fatty acid degradation	11	0.7	vda00071
Glyoxylate and dicarboxylate metabolism	11	0.7	vda00630
Glycerophospholipid metabolism	11	0.7	vda00564
Sulfur metabolism	11	0.7	vda00920
Pantothenate and CoA biosynthesis	11	0.7	vda00770
Nicotinate and nicotinamide metabolism	10	0.6	vda00760
Autophagy—yeast	9	0.5	vda04138
Endocytosis	9	0.5	vda04144
Fatty acid metabolism	9	0.5	vda01212
Phenylalanine. tyrosine and tryptophan biosynthesis	8	0.5	vda00400
Ubiquinone and other terpenoid-quinone biosynthesis	8	0.5	vda00130
Valine. leucine and isoleucine biosynthesis	8	0.5	vda00290
Porphyrin and chlorophyll metabolism	8	0.5	vda00860
Spliceosome	8	0.5	vda03040
Pyrimidine metabolism	7	0.4	vda00240
Arginine biosynthesis	7	0.4	vda00220
Nitrogen metabolism	7	0.4	vda00910
Aminoacyl-tRNA biosynthesis	7	0.4	vda00970
Glutathione metabolism	6	0.4	vda00480
Folate biosynthesis	6	0.4	vda00790
Ubiquitin mediated proteolysis	6	0.4	vda04120
Terpenoid backbone biosynthesis	6	0.4	vda00900
Nucleotide excision repair	6	0.4	vda03420
Citrate cycle (TCA cycle)	6	0.4	vda00020
Other glycan degradation	5	0.3	vda00511
Mitophagy—yeast	5	0.3	vda04139
Base excision repair	5	0.3	vda03410
Taurine and hypotaurine metabolism	5	0.3	vda00430
Proteasome	5	0.3	vda03050
Sphingolipid metabolism	5	0.3	vda00600
MAPK signaling pathway—yeast	5	0.3	vda04011
Various types of N-glycan biosynthesis	5	0.3	vda00513
Phagosome	5	0.3	vda04145
Ascorbate and aldarate metabolism	5	0.3	vda00053
Fatty acid elongation	4	0.2	vda00062
Riboflavin metabolism	4	0.2	vda00740
SNARE interactions in vesicular transport	4	0.2	vda04130
N-Glycan biosynthesis	4	0.2	vda00510
One carbon pool by folate	4	0.2	vda00670
Vitamin B6 metabolism	4	0.2	vda00750
mRNA surveillance pathway	4	0.2	vda03015
Carotenoid biosynthesis	4	0.2	vda00906
Linoleic acid metabolism	3	0.2	vda00591
Ether lipid metabolism	3	0.2	vda00565
Meiosis—yeast	3	0.2	vda04113
Synthesis and degradation of ketone bodies	3	0.2	vda00072
Cell cycle—yeast	3	0.2	vda04111
Glycosylphosphatidylinositol (GPI)-anchor biosynthesis	3	0.2	vda00563
Arachidonic acid metabolism	3	0.2	vda00590
Sulfur relay system	3	0.2	vda04122
Basal transcription factors	3	0.2	vda03022
Thiamine metabolism	3	0.2	vda00730
Selenocompound metabolism	3	0.2	vda00450
Glycosphingolipid biosynthesis—globo and isoglobo series	3	0.2	vda00603
Phosphonate and phosphinate metabolism	2	0.1	vda00440
Non-homologous end-joining	2	0.1	vda03450
RNA polymerase	2	0.1	vda03020
Autophagy—other	2	0.1	vda04136
Biosynthesis of unsaturated fatty acids	2	0.1	vda01040
Alpha-Linolenic acid metabolism	2	0.1	vda00592
Lysine biosynthesis	2	0.1	vda00300
DNA replication	2	0.1	vda03030
Protein export	2	0.1	vda03060
Inositol phosphate metabolism	2	0.1	vda00562
Biotin metabolism	2	0.1	vda00780
Mismatch repair	2	0.1	vda03430
Mannose type O-glycan biosynthesis	1	0.1	vda00515
Lipoic acid metabolism	1	0.1	vda00785
Other types of O-glycan biosynthesis	1	0.1	vda00514
Phosphatidylinositol signaling system	1	0.1	vda04070
Monobactam biosynthesis	1	0.1	vda00261
Fatty acid biosynthesis	1	0.1	vda00061
Sesquiterpenoid and triterpenoid biosynthesis	1	0.1	vda00909
Caffeine metabolism	1	0.1	vda00232
AGE-RAGE signaling pathway in diabetic complications	1	0.1	vda04933
Carbapenem biosynthesis	1	0.1	vda00332
ABC transporters	1	0.1	vda02010
Glycosaminoglycan degradation	1	0.1	vda00531

### *V. dahliae* growth is reduced in *Arabidopsis* JA mutants

Former studies indicate that the accumulation and perception of jasmonates are key events for both plant and fungal responses to the interaction. The plant defense machinery is activated by an elevation of jasmonates and activation of the receptor COI1, while the fungus activates the plant COI1-dependent JA pathway to induce disease symptom development in the host (Feys et al., 1994; Xie et al., 1998; Ralhan et al., 2012). Upregulation of genes involved in JA biosynthesis and responses within the first 24 h of co-cultivation demonstrates that the course is already set even before significant changes in the plant hormone levels can be detected (cf. below). We observed several DEGs involved in the α-linolenic acid pathway as well as in plant hormone signal transduction pathways (Table 2 and Figure 2A). While the expression of growth-associated genes like the auxin-responsive genes *ARF5* (Krogan et al., 2016) and several members of the *GH3*-family (e.g., *GH3.17, GH3.4, DFL2, WES1*, Staswick et al., 2005) was decreased, an up-regulation of the JA biosynthetic genes *LIPOXYGENASE 3* and *4* (*LOX3* and *4*, Acosta and Farmer, 2010; Umate, 2011) and *OXOPHYTODIENOATE-REDUCTASE 3* (*OPR3*, Müssig et al., 2000) as well as of an ET response factor (*ERF1*, Fujimoto et al., 2000) was observed.

**Figure 3:**
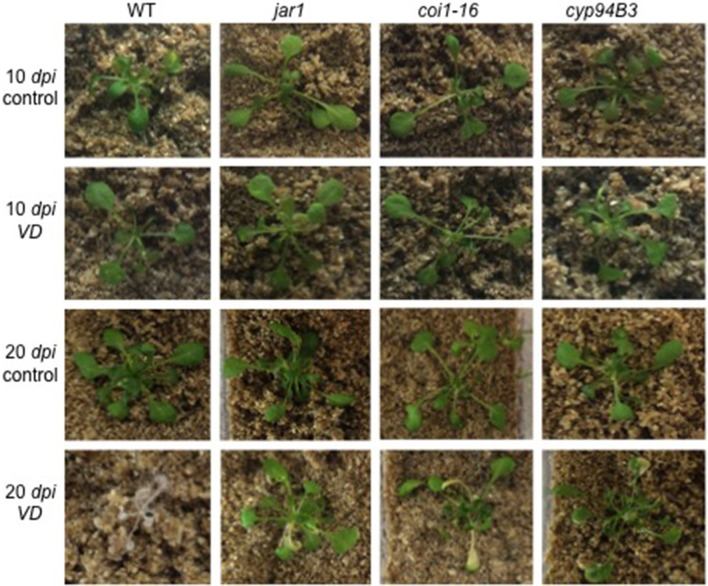
Phenotype of *V. dahliae (VD)*-infected *Arabidopsis* WT and JA mutant plants. Shown are representative phenotypes of WT, *jar1, coi1-16*, and *cyp94B3* plants 10 (upper two rows) and 20 *dpi* (lower two rows) grown in Magenta boxes. 14 day-old plants were treated with water (control, *n* = 6) or *VD* (*n* = 9) for 24 h and then transferred to the boxes.

**Figure 4:**
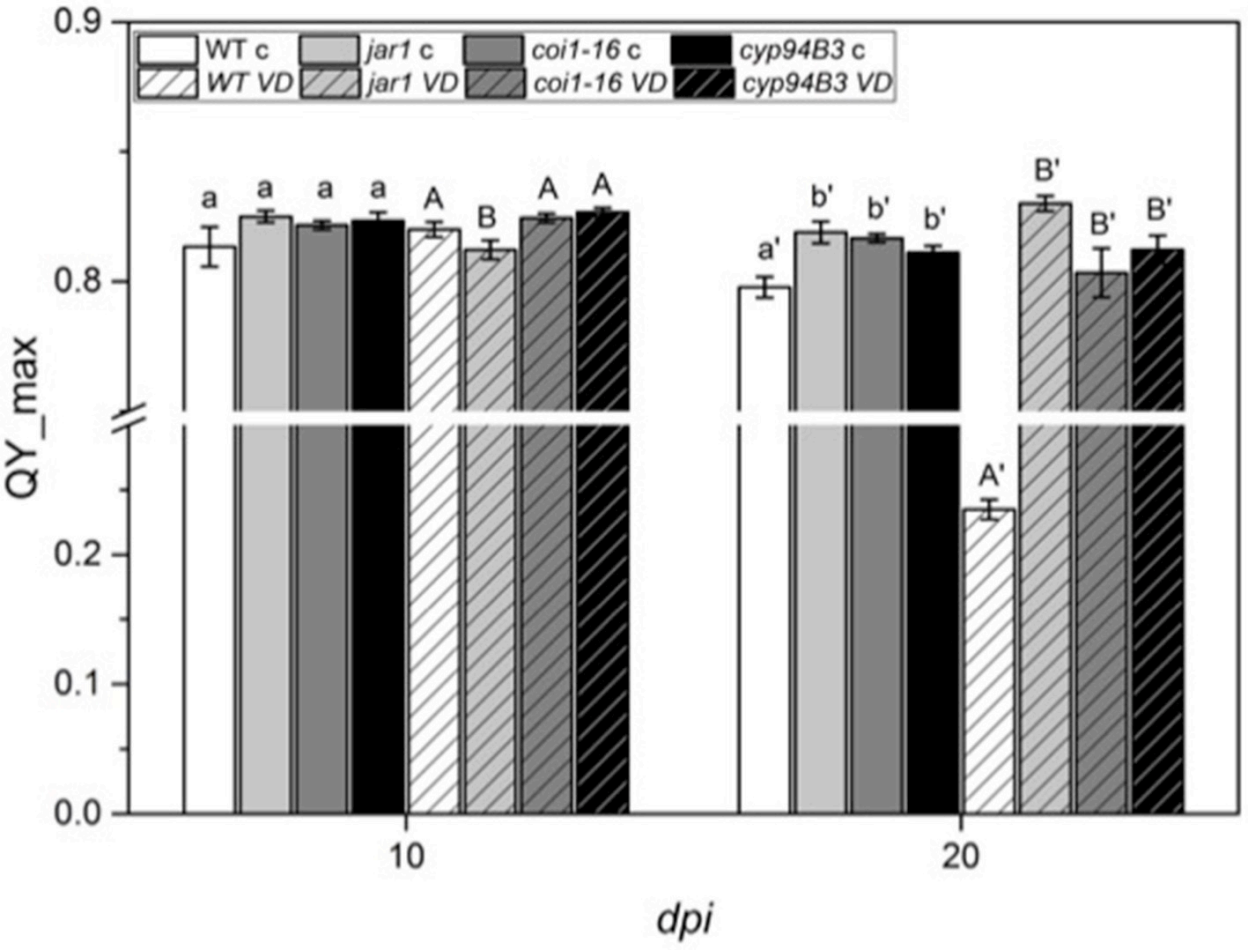
Chlorophyll fluorescence (QY_max) analysis of *Arabidopsis* WT and JA mutant plants after 24 h of *V. dahliae* infection. Shown are the QY_max values [± SE, *n* = 6 (control) and 9 (*VD*-infected)] in control (plain color) and *VD*-infected (stripes) plants 10 and 20 *dpi*. QY_max was analyzed in WT (white), *jar1* (light gray), *coi1-16* (dark gray) and *cyp94B3* (black) plants. Statistically significant differences between controls and between infected plants were analyzed by one-way ANOVA separately, *p* < 0.05 (Sidak). Different letters indicate a statistically significant difference.

To further investigate the role of JA in the *Arabidopsis*-*V. dahliae* interaction, we analyzed the phenotype and vitality of *Verticillium*-infected JA mutants *jar1* (Staswick et al., 1992; Staswick and Tiryaki, 2004), *coi1-16* (Ellis and Turner, 2002), and *cyp94B3* (Koo et al., 2011). Although there was no visual difference between the mutants and the WT plants 10 *dpi*, the WT plants were dead after 20 *dpi*, while the mutant plants showed disease symptoms in leaves but were still vital and alive (Figure 3). This result was confirmed by analysis of the efficiency of the electron transfer during photosynthesis using chlorophyll fluorescence measurements, a sensitive marker for plant vitality (Figure 4). At 10 *dpi*, there were only small differences between the mutants and the WT, and chlorophyll fluorescence values around 0.81 indicate that the plants were capable of photosynthesis. At 20 *dpi*, the JA mutants still possessed a fluorescence value between 0.81 and 0.83, while WT plants were at 0.24 corresponding to very low levels of electron transport and photosynthetic efficiency. Thus, these non-invasive measurements provide an efficient tool to determine and quantify disease development in *Verticillium*-infected hosts. To obtain further insight into the colonization efficiency of *V. dahliae*, the content of fungal RNA—in the plants previously analyzed for chlorophyll fluorescence—was determined in roots and shoots separately, and the levels were compared to those in WT plants (Figure S2). The colonization pattern at the two tested time points was very different. While *coi1-16* and *cyp94B3* roots showed a significantly lower colonization at 10 *dpi* compared to WT (38 and 30%, respectively), *jar1* roots showed an intermediate level with 83% (Figure S2A). The respective shoots of the plants show the same trend (Figure S2B). At 20 *dpi*, no difference in colonization of the mutant shoots was detectable; all of them showed significantly lower fungal RNA levels compared to the WT (Figure S2D). The levels of fungal RNA in the roots of *coi1-16* plants was significantly lower compared to those of all other genotypes.

### Root colonization and plant hormone levels are not altered after the first 24h of co-culture with *V. dahliae*

**Figure 5:**
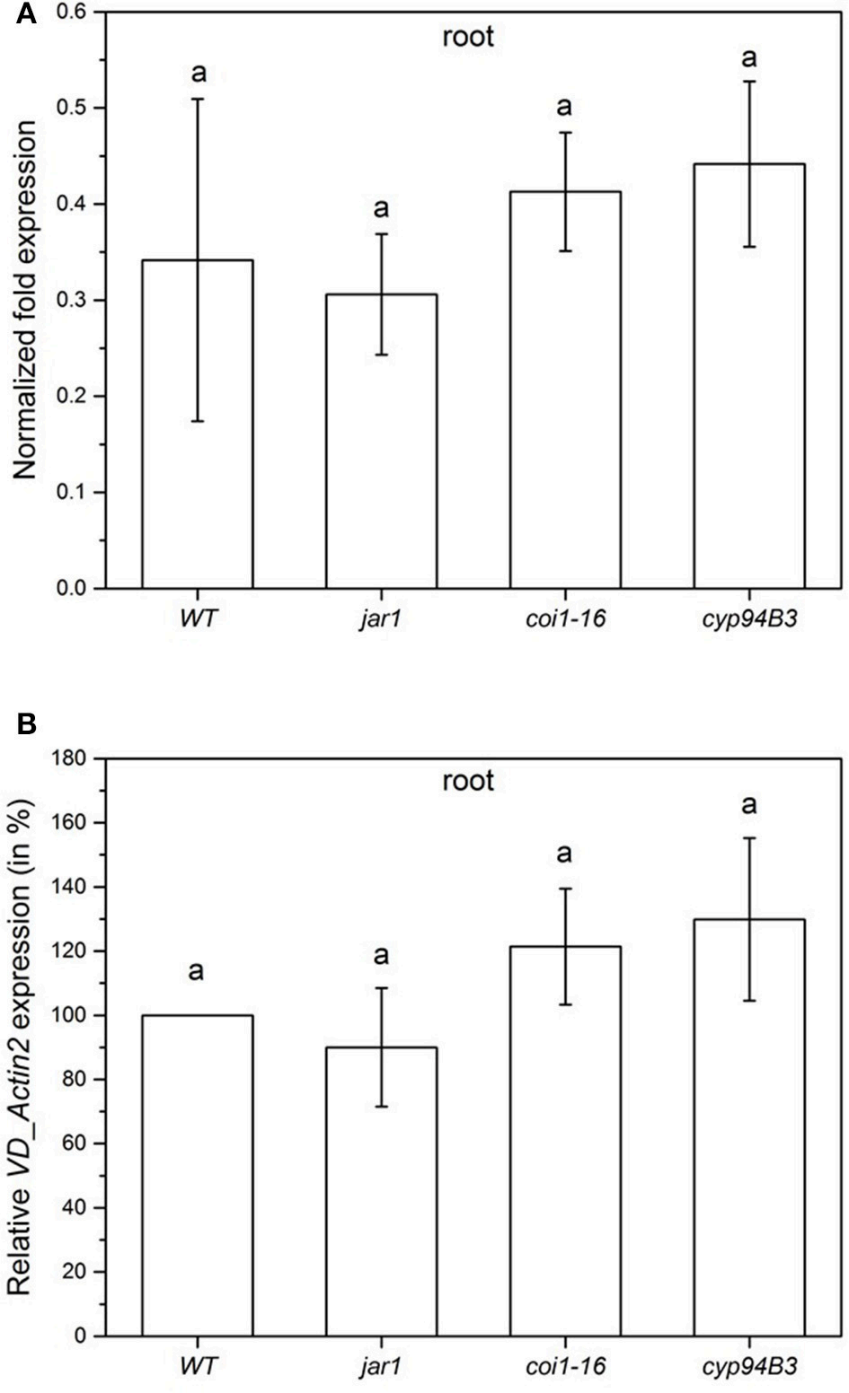
Colonization of *Arabidopsis* WT and JA mutant plants by *V. dahliae* after 24 h. Shown is the normalized fold expression (± SE, *n* = 3) of *VD_Actin2* in *VD*-infected WT, *jar1, coi1-16* and *cyp94B3* plants **(A)** and the relative expression in % **(B)**. The expression level of *VD_Actin2* in *VD*-infected WT plants was used as control and set to 1.0. The mRNA levels for each cDNA probe were normalized with respect to the *RPS18B* mRNA level. Statistically significant differences between the mutants were analyzed by one-way ANOVA, *p* < 0.05 (Sidak). Different letters indicate a statistically significant difference.

To compare the colonization of the mutant plants in the very early phase, the content of fungal RNA was also analyzed 24 h after co-cultivation (Figure 5). All plants showed a similar fungal colonization with no statistically significant differences (Figure 5A). Compared to the WT colonization level (set as 100%), *jar1* plants showed a colonization of 90.0%, *coi1-16*, and *cyp94B3* of 121.4 and 129.8%, respectively (Figure 5B). These results suggest that there are also no differences in plant JA levels during the first 24 h of co-culture.

**Figure 6:**
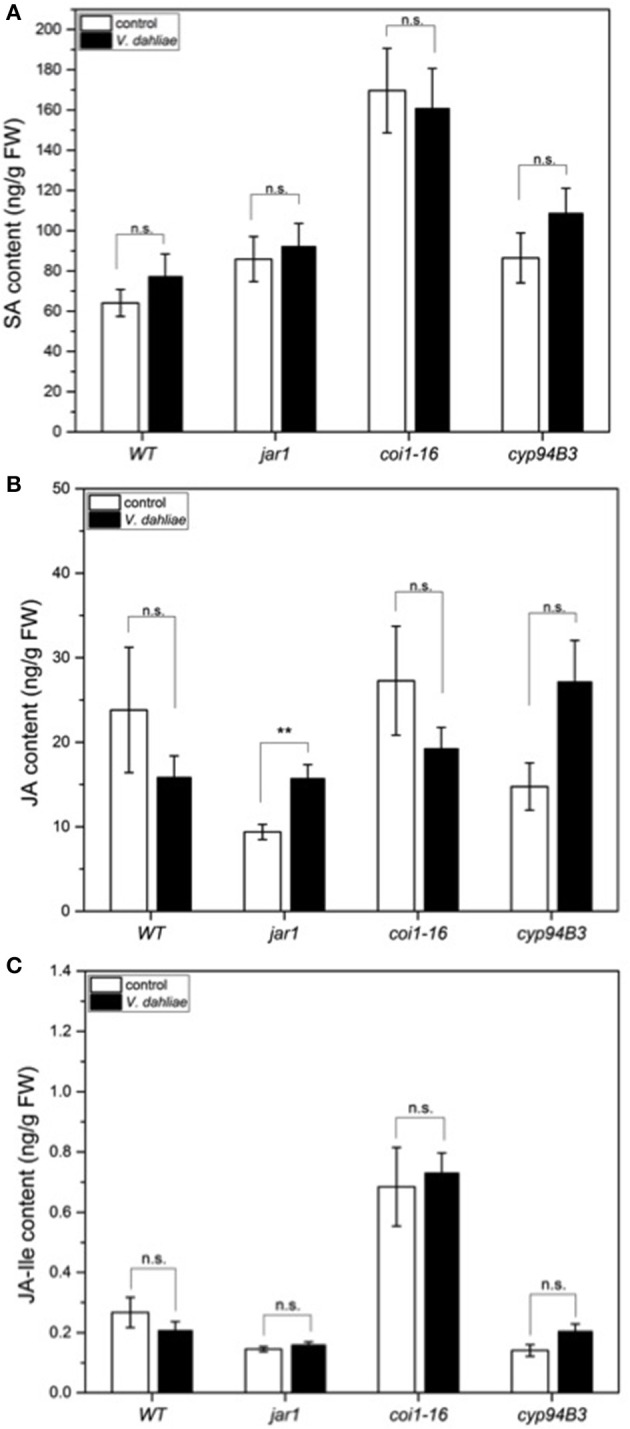
Phytohormone levels in *Arabidopsis* WT and JA mutant plants after 24 h of *V. dahliae* infection. Shown is the content (± SE, *n* = 10) of SA **(A)**, JA **(B)**, and JA-Ile **(C)** in control (white) and *VD*-infected (black) WT, *jar1, coi1-16*, and *cyp94B3* plants. Statistically significant differences were analyzed by Mann Whitney U-test, *p* < 0.05; n.s. not significant, ^**^p < 0.01.

In a previous study we demonstrated that *V. dahliae* infection leads to an elevation of JA, JA-Ile, and SA 21 *dpi* (Sun et al., 2014). To analyze whether the accumulation of these phytohormones was already induced in the first 24 h, leaf tissue of WT and the mutants was analyzed, but the content of SA (Figure 6A) and JA-Ile (Figure 6C) were found not to be significantly induced by the fungus. There was also no difference in the JA content in WT, *coi1-16* and *cyp94B3*, while a slight difference was observed in *jar1* plants. Accumulation of JA in this mutant is caused by the inactivation of the Ile-conjugating enzyme. These data confirm that the changes in the gene expression profiles described above occur before the fungus induces changes in the plant phytohormone levels. Thus, the identified genes respond most likely to fungal signals and not to fungus-induced phytohormone changes in the plant. JA signaling in the host is necessary for disease development only during later phases of the interaction, which can be strongly repressed or retarded when JA function is impaired.

### Gene expression in JA mutants is different to that in the WT in the early phase of colonization

**Figure 7:**
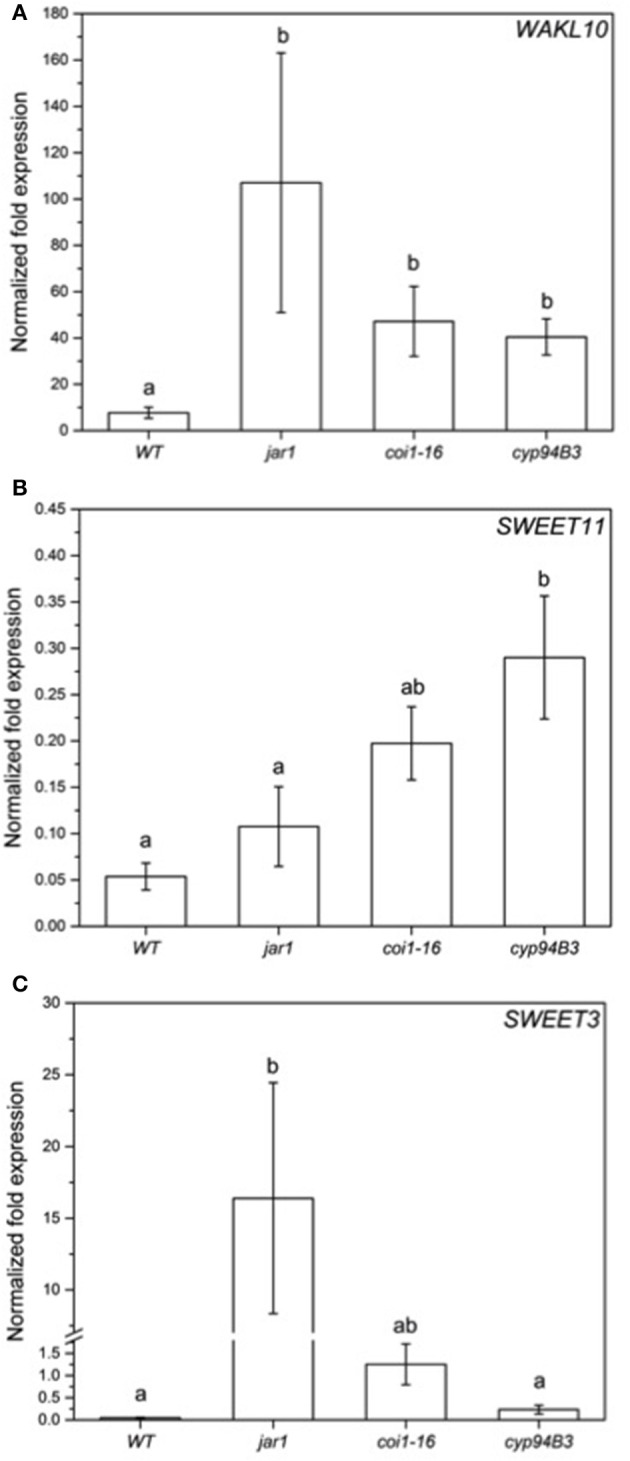
Expression of selected genes in *Arabidopsis* WT and JA mutant plants after 24 h of *V. dahliae* infection. Shown is the normalized fold expression (± SE, *n* = 3) of *WAKL10* **(A)**, *SWEET11* **(B)**, and *SWEET3* **(C)** in *VD*-infected WT, *jar1, coi1-16*, and *cyp94B3* plants. The expression level of genes of interest (GOIs) in water-treated plants was used as control and set to 1.0. The mRNA levels for each cDNA probe were normalized with respect to the *RPS18B* mRNA level. Statistically significant differences between the mutants were analyzed by one-way ANOVA, *p* < 0.05 (Sidak). Different letters indicate a statistically significant difference.

**Figure 8:**
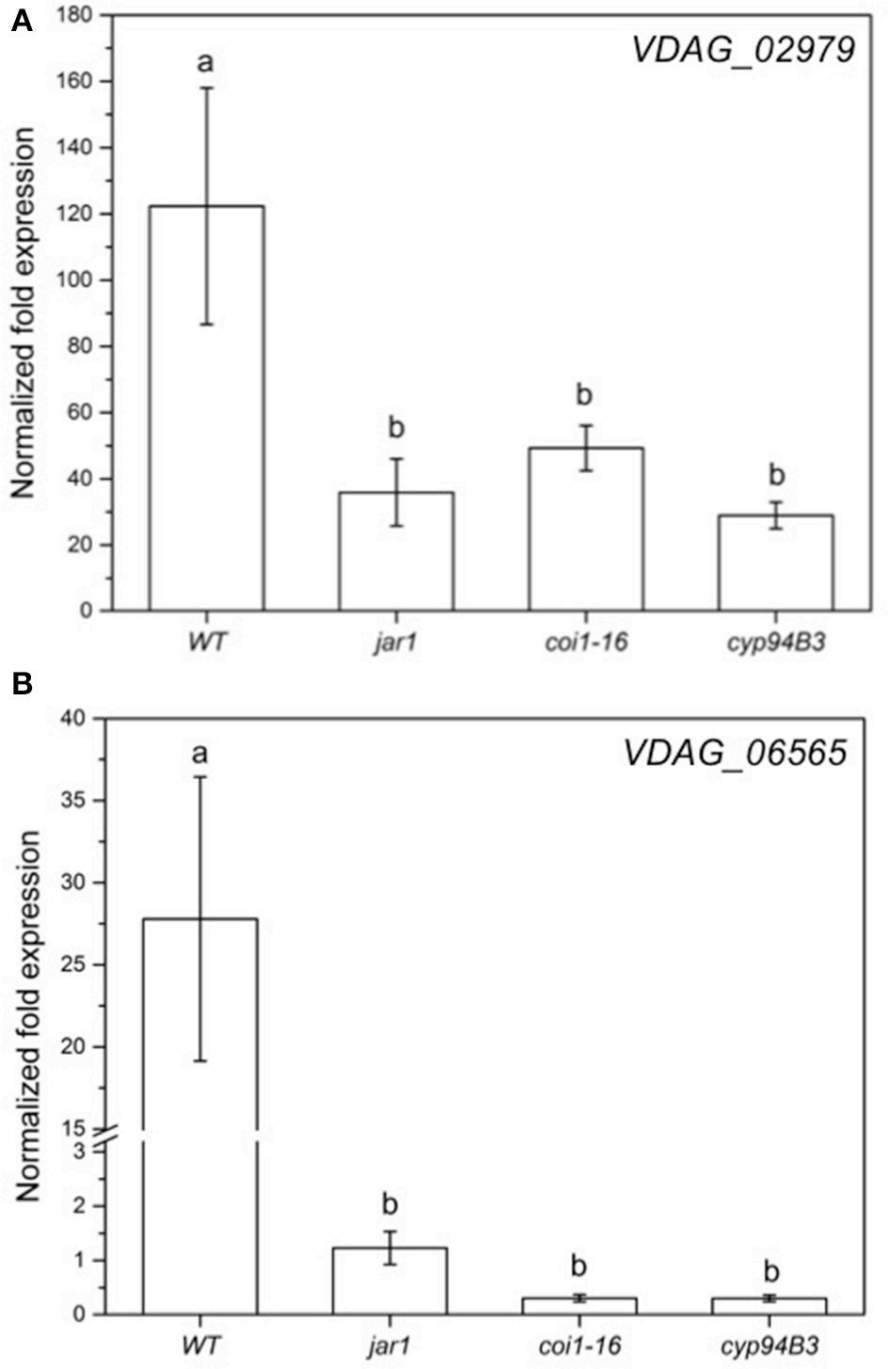
Expression of selected genes in *V. dahliae* after colonizing *Arabidopsis* WT and JA mutant plants for 24 h. Shown is the normalized fold expression (± SE, *n* = 3) of *VDAG_02979* **(A)** and *VDAG_06565 (PARN)* **(B)** in *VD*-colonized WT, *jar1, coi1-16*, and *cyp94B3* plants. The expression level of GOIs in *VD* alone was used as control and set to 1.0. The mRNA levels for each cDNA probe were normalized with respect to the *VD_Actin2* mRNA level. Statistically significant differences between the mutants were analyzed by one-way ANOVA, *p* < 0.05 (Sidak). Different letters indicate a statistically significant difference.

To validate the RNA-seq results obtained, the expression of selected regulated genes in the data set was analyzed by RT-qPCR for co-cultivated WT plants (Figure 7) and for the fungus (Figure 8). Highly regulated members of the SWEET family (Chen et al., 2010) and a Wall-Associated Kinase-like gene (*WAKL10*, Meier et al., 2010) were chosen for the plant and *VDAG_02979* (a glucose transporter) and *VDAG_06565* (poly-A-ribonuclease, PARN) for the fungus. In the RNA-seq analysis, the expression of *WAKL10* was 56-fold induced in *V. dahliae*-infested plants compared to plants grown alone. A 10-fold induction was observed in the RT analysis (Figure 7A). *SWEET11* and -*3* were 20- and 10-fold downregulated in the RNA-seq data set, respectively, while a 9.7- and 6.8-fold decrease was detected by RT-qPCR (Figures 7B,C). Interestingly, the expression of these genes was differently regulated in the JA mutants. The expression of *WAKL10* was induced ~100-fold in *jar1*, i.e., significantly higher than in WT seedlings, while *SWEET3*, which is down-regulated in the WT, was induced 17-fold in the mutant (Figure 7). Additionally, *SWEET11* was less repressed in the mutants: for *jar1*, the repression was 50% lower compared to WT plants. This clearly indicates that the expression of interaction-specific plant genes is influenced by JA content.

The RNA-seq results for the fungal genes were also validated by RT-qPCR analysis (Figure 8). The mRNA levels of *VDAG_02979* (RNA-seq: + 200-fold; RT-qPCR: + 120-fold) and *VDAG_06565* (RNA-seq: + 3-fold; RT-qPCR: + 27-fold) in response to the fungus showed the same trend. Again, when co-cultivation was performed with the JA mutants, the expression of both genes was less induced compared to WT plants (Figure 8). Thus, both partners require plant JA for the typical regulation of genes that respond to the symbiotic interaction.

## Discussion

### *V. dahliae* is in the pre-vascular growth phase 24h after co-cultivation with *Arabidopsis*

The propagation of *Verticillium* species within their hosts is characterized by a biphasic interaction: initially a biotrophic phase allows rapid growth of the microbe in the xylem without major effects on plant performance followed by a necrotrophic phase in which the host is ultimately killed (Reusche et al., 2012). Analysis of the initial contact and early phase of the interaction between both partners could lead to the identification of target genes that will help understand further phases of the interaction and provide useful targets for pest control. Confocal analysis 24 h after co-cultivation demonstrated that the pathogen colonizes the root efficiently (Figure 1) by developing a dense hyphal network at the root surface and root tip. In areas of lateral root formation, an accumulation of conidia spores was detectable (Figures 1G–I). This matches previous observations that showed the lateral root to be a primary area of *Verticillium* infection in diverse host plants (Zhou et al., 2006; Vallad and Subbarao, 2008; Zhao et al., 2014). Further analyses of root cross-sections demonstrated that the fungus invades the plant tissue but did not reach the vascular system within the first 24 h of co-culture (Figures 1J–O). We conclude that the fungus was still growing in the pre-vascular phase during our experiments, optimal for the identification of genes regulated during the very early interaction phase. During this period we also did not observe significant differences in phytohormone levels, which is consistent with the microscopic information.

### Initial contact of *Arabidopsis* and *V. dahliae* leads to reprogramming of primary metabolism in both organisms

Plant-pathogen interactions involve an adaptation of both partners. While the plant typically recognizes the pathogen and induces appropriate defense responses, the fungus manipulates the biology of its host to gain sufficient nutrients for growth and reproduction (Boyd et al., 2013). To identify early targets in the *Arabidopsis*—*V. dahliae* interaction, an RNA-seq analysis after 24 h of co-cultivation was performed for both partners. This time point was chosen because no structural changes were detected in plant and fungal tissues under the microscope, and because the phytohormone levels in the plant were not yet altered, thereby avoiding secondary effects due to the response of the plant genome to an altered phytohormone environment. We detected 4432 DEGs for both organisms (Table 1, Tables S1–S4), 1143 DEGs in *Arabidopsis* belong mainly to primary metabolic pathways (21%, Figure 2A). This result is comparable to a recent study in cotton where 26% of the DEG reads could be assigned to metabolic pathways (Zhang W. W. et al., 2017). Comparably, in both studies, pathways associated with plant-pathogen interaction (3%) and plant hormone signal transduction (2%) were also regulated upon *Verticillium* stress.

Several members of the plant sucrose efflux transporters of the SWEET family were highly regulated in the analyzed interaction. *SWEET11* as well as –*3*, −*12*, −*15*, and –*8* were downregulated in decreasing order (Table S2). SWEET11 and −12 are involved in phloem loading with sugars (Chen et al., 2010, 2012; Boyd et al., 2013). By silencing of *OsSWEET11* in rice it could be demonstrated that the growth of *Xanthomonas oryzae pv. oryzae* (Xoo) is decreased which resulted in more resistant plants (Yang et al., 2006). Also *SWEET3* was reported to be involved in defense and was downregulated after Geminivirus infection (Ascencio-Ibá-ez et al., 2008). These observations suggest that the plant is trying to prevent export of its sugars to restrict fungal growth.

Besides the defense genes discussed above, members of the *WAK* family are also highly represented in our data sets. *WAK-L10 and WAK3*, –*5*, −*1*, and −*4* responded to *Verticillium* treatment (in declining order, Table S1). *WAK1* is induced upon infection with *Ps. maculicola* ES4326 and the protein stimulated *PR1* expression (He et al., 1998). Additionally, *WAK1*, −*2*, −*3*, and −*5* are inducible by SA which accumulated during pathogen infection (He et al., 1999). Likewise, the WAK-like gene *WAKL10* is SA-induced and involved in defense against bacteria and fungi (Meier et al., 2010). Among the genes involved in controlling Ca^2+^ homeostasis, genes for in- and efflux transporters of the cyclic nucleotide-gated channel (CNGC), autoinhibited Ca^2+^-ATPase (ACA), and glutamate-like receptor (GLR) protein families responded to the fungus, indicating that many signaling events induced during the early phase of the interaction are Ca^2+^-dependent. Consistent with this idea, genes for Ca^2+^-binding proteins involved in signal perception and propagation showed increased expression, especially members of the calmodulin-like (CML) protein family. However, closer inspection of the genes did not allow any meaningful conclusion about which pathways are major targets of the fungus. Besides regulation of defense responses, many genes for Ca^2+^-binding proteins are involved in ion homeostasis, enzyme activity control, and biotrophic plant/microbe interactions (for details compare genes in Table S1 with the TAIR database). An induction of defense genes is always accompanied by a lower investment in plant growth (Huot et al., 2014). This is reflected by the down-regulation of *ROOT CAP POLYGALACTURONASE28* and enzymes involved in cytokinin signaling. The phase of reprogramming of plant primary metabolism lasts for several days, as in *Verticillium*-infected tomato plants where it was shown that both gene expression and protein synthesis of metabolic pathways proteins are still down-regulated at 7 *dpi* (van Esse et al., 2009; Witzel et al., 2017). The global transcriptome analysis performed in this study provides significant insights into components involved in early phases of the *Arabidopsis*—*V. dahliae* interaction and suggests a number of genes and pathways that could be employed as markers in breeding for wilt tolerance.

In contrast to previous studies focusing on analysis of the plant's reaction to the fungus (van Esse et al., 2009; Faino et al., 2012; Witzel et al., 2017; Zhang W. W. et al., 2017), we present also DEGs from the fungus caused by response to the plant. The majority of the 3289 DEGs code for proteins with unknown functions (Tables S3, S4). The up-regulation of *VDAG_02979*, which encodes for a glucose transporter, suggests that it might play an important role in the early interaction. Besides this initial observation, a huge number of identified genes belong to families which might become important targets after elucidation of their function in *V. dahliae*. Therefore, this list might provide useful information for genes with interaction-specific functions, in particular since the knowledge about the function of *V. dahliae* genes is strongly increasing. For example, very recent studies revealed that homeodomain and bZIP transcription factors (Fang et al., 2017; Sarmiento-Villamil et al., 2017), two less characterized transcription factors (Sarmiento-Villamil et al., 2010; Zhang W. Q. et al., 2017), polyketide synthases (Zhang T. et al., 2017), endochitinases (Cheng et al., 2017), a novel *V. dahliae* protein that targets the plant nucleus (Zhang L. et al., 2017), LysM effectors (Kombrink et al., 2017), a isochorismatase hydrolase (Zhu et al., 2017), a factor involved in the fungal secretory pathway (Xie et al., 2017), a RACK1-like protein involved in root entry (Yuan et al., 2017), pathogenesis-related exudated proteins (Chen et al., 2016), and the mitogen-activated protein kinase 2 (Tian et al., 2016) are important components in controlling *V. dahliae*-induced disease development in various plant species. Members of all these protein families can be found in the list of *V. dahliae* genes up-regulated after infection of *Arabidopsis*.

### JA mutants are less susceptible to *Verticillium* infection

Various studies have shown the involvement of several classes of plant hormones in the control of *Verticillium* growth and propagation in *Arabidopsis*. While ET perception mutants are more susceptible to *Verticillium* infection, an elevation of cytokinins enhances plant resistance (Pantelides et al., 2010; Reusche et al., 2013; Sun et al., 2014). Downregulation of plant genes involved in cytokinin signaling might therefore be induced by the fungus. Interestingly, jasmonates are not only accumulated by the plant to induce defense, but the fungus also requires a JA-independent COI1 function in roots to elicit disease symptoms in *Arabidopsis* shoots (Ralhan et al., 2012). To further analyze the role of JA levels in the interaction of *Arabidopsis* and *V. dahliae*, JA biosynthesis (*jar1*), perception (*coi1-16*) and degradation (*cyp94B3*) mutants were studied. All of these mutants performed better, showed less severe disease symptom development in the leaves at 20 *dpi* compared to the WT control, which were already dead at this time point (Figure 3), and had a higher photosynthetic potential, as demonstrated by their QY_max value above 0.80 (F_v_/F_m_, Figure 4) (e.g., Kim et al., 2009; Sztatelman et al., 2015). The observation, that *jar1* plants performed better than WT plants, contradicts earlier findings where *jar1* plants were as susceptible as WT plants (Fradin et al., 2011). To analyze this contradiction, the colonization of the mutants used was compared to WT plants (Figure S2). While there was no difference to the WT colonization level after 10 *dpi* in the roots and the shoots, there was a significant difference at 20 *dpi*. The colonization level of the root was similar to WT while the colonization in the shoots was significantly lower in *jar1* (Figure S2D). This observation could explain the better performance of the aerial parts of the *jar1* plants. Taken together, the altered expression of the interaction-specific genes of plant and fungal origin in the JA mutants confirms the important role of this phytohormone also during early phases. Apparently, the altered expression profile occurs before a significant change in phytohormone levels become detectable.

To gain insight into the growth behavior of *V. dahliae* on JA mutants during the first 24 h of co-cultivation, the colonization of the roots was analyzed. The detected differences in the colonization (Figure 5) were not significant at this time point, but may have a greater impact in later phases where a clear difference is obvious. After 24 h of co-cultivation, there was also no detectable difference in the levels of the phytohormones SA, JA and JA-Ile (Figure 6). Since changes in phytohormone levels upon pathogen attack are normally very rapid in plants, *Arabidopsis* might have not yet recognized the microbe as friend or foe, in spite of the already initiated reprogramming of its gene expression pattern. It is also conceivable that the penetration rate is still too low to induce the accumulation of JA and JA-Ile, since this is often associated with wounding or pathogen-induced cell disruption, which was not visible in our microscopic studies (e.g., Suza and Staswick, 2008; Koo et al., 2009). The biphasic interaction with an initial biotrophic period followed by a necrotrophic period may also leave the plant undecided whether it responds to the pathogen with SA- or JA-dependent defense strategies. Furthermore, both phytohormones cross-talk (Thaler et al., 2012; Proietti et al., 2013).

The low content of active JA-Ile in *jar1* plants and the reduced perception in the receptor mutant *coi1-16* lead to a decreased activation of the downstream signaling pathway by the receptor complex SCF^COI1^ (Thines et al., 2007). Since *Verticillium* propagation from the roots to the leaves depends on an activated COI pathway (Ralhan et al., 2012), this could be a reason why *Verticillium* causes a reduced leaf growth rate of the JA mutant plants (Figure 3). The reduced spread in the green parts of the JA mutants is also reflected in the gene expression analysis of chosen genes for both the plant and the fungus (Figures 7, 8). In the mutant plants, the SA-induced defense gene *WAKL10* is more highly expressed than in WT plants and the downregulation of *SWEET11* is less pronounced (Figure 7). In the fungus, both target genes *VDAG_02979* and *VDAG_06565* are more weakly expressed in the mutants than in the WT plants (Figure 8). *VDAG_02979* codes for a glucose transporter (http://fungi.ensembl.org/Verticillium_dahliae), which contributes to the nutrient supply of the fungus. The lower growth rate in the mutants may be a consequence of this transporter being less expressed.

In conclusion, biotrophic plant-microbe interactions are characterized by the stimulation of SA, but not JA levels, while the opposite hormone regulation occurs during nectrophophic interactions (reviewed in Chanclud and Morel, 2016). Within the first 24 h of interaction studied here, none of these phytohormone levels increase significantly, while hormone-synthesis related genes, as well as defense-related genes responding to both hormone types are already up-regulated in the host. This suggests that no clear decision has been taken yet about which strategy will follow initial contact. The numerous genes identified during early reprogramming of the fungal and plant development might be crucial for the initiation and propagation of the pest, and thus may be helpful for developing strategies which potentially restrict fungal development after infection. Considering our results, identification of crucial players which control the interaction at early stage is apparently difficult, because many metabolomic pathways are already re-adjusted within the first 24 h of the contact of the two partners.

The strong retardation of disease symptom development in host plants impaired in jasmonate mutants has been attributed to the fact that *Verticillium* stimulates the host JA functions in order to promote host cell death during the later necrotrophic phase. There is a substantial crosstalk between JA and SA signaling in which each hormone inhibits the accumulation and/or function of the other. Complete or strong inhibition of JA functions in the mutants may favor SA accumulation and/or SA signaling function which—in turn—may prolong the biotrophic phase and thus retard necrosis and disease development.

## Author contributions

SS: designed and performed the experiments; WS-H: analyzed the RNA-seq data and DEGs; AF: prepared, analyzed, and evaluated samples for confocal microscopy; MR: analyzed the phytohormones; RG, JG, and RO: supervised the projects; RO: coordinated the project; SS, JG, and RO: wrote the manuscript; All authors read and approved the final manuscript.

## Data access

Raw and calculated RNA-seq data was submitted to NCBIs Gene Expression Omnibus (GEO, http://www.ncbi.nlm.nih.gov/geo/) under the accession number GSE104590.

### Conflict of interest statement

The authors declare that the research was conducted in the absence of any commercial or financial relationships that could be construed as a potential conflict of interest.
