# Supplementary material for: Verticillium dahliae-Arabidopsis Interaction Causes Changes in Gene Expression Profiles and Jasmonate Levels on Different Time Scales
Source: Front Microbiol. 2018 Feb 13;9:217. doi: 10.3389/fmicb.2018.00217 (PMC5819561; doi:10.3389/fmicb.2018.00217)
Supplement: Supplementary file 1 [file Table1.DOCX]

**Supplemental tables**

**Table S1.** List of up-regulated plant genes in *A. thaliana* (ATH) and *V. dahliae* (VDA) co-culture compared to *A. thaliana* grown separately. Cut-off was made at a log_2_-fold change of 1.33.

| **GeneID** | **Symbol** | **base Mean** | **log_2_ Fold Change** | **Fold Change** |
| --- | --- | --- | --- | --- |
| **AT4G31970** | *CYP82C2* | 23053.0 | 11.0 | **1995.0** |
| **AT2G30750** | *CYP71A12* | 41627.3 | 8.9 | **477.3** |
| **AT1G26380** | *FOX1* | 15693.6 | 8.6 | **385.7** |
| **AT1G26410** | *ATBBE6* | 4503.8 | 7.9 | **243.9** |
| **AT2G30770** | *CYP71A13* | 1312.2 | 7.3 | **161.1** |
| **AT5G19880** | *PER58* | 1255.7 | 6.8 | **114.2** |
| **AT1G26390** | *ATBBE4* | 6868.1 | 6.8 | **111.8** |
| **AT3G12910** | *NAC (No Apical Meristem) domain transcriptional regulator* | 485.3 | 6.7 | **106.0** |
| **AT1G02930** | *GSTF6* | 85808.1 | 6.4 | **86.5** |
| **AT2G25297** | *transmembrane protein* | 469.6 | 6.3 | **81.3** |
| **AT1G66700** | *PXMT1* | 4429.3 | 6.2 | **75.4** |
| **AT5G44990** | *glutthione s transferase* | 535.3 | 6.2 | **75.3** |
| **AT3G24510** | *defl* | 260.0 | 6.1 | **68.0** |
| **AT3G60120** | *BGLU27* | 366.5 | 6.0 | **63.1** |
| **AT4G23280** | *CRK20* | 472.8 | 5.9 | **59.9** |
| **AT4G11170** | *RMG1* | 808.3 | 5.9 | **59.0** |
| **AT5G57220** | *CYP81F2* | 16076.1 | 5.9 | **58.3** |
| **AT4G19970** | *nucleotide diphospho sugar transferase* | 312.7 | 5.8 | **56.2** |
| **AT1G79680** | *WAKL10* | 821.8 | 5.8 | **56.0** |
| **AT4G28420** | *tyrosine transaminase* | 280.8 | 5.8 | **55.8** |
| **AT4G21380** | *SD18* | 544.7 | 5.8 | **54.6** |
| **AT5G56960** | *BHLH41* | 325.1 | 5.8 | **54.3** |
| **AT2G29350** | *SAG13* | 356.7 | 5.7 | **53.4** |
| **AT5G40990** | *GLIP1* | 1861.5 | 5.7 | **51.2** |
| **AT2G23270** | *transmembrane protein* | 299.5 | 5.6 | **49.6** |
| **AT5G37840** | *plastid movement impaired protein* | 182.2 | 5.6 | **49.5** |
| **AT1G02920** | *GSTF7* | 123467.0 | 5.5 | **45.7** |
| **AT1G67980** | *CCOAMT* | 4064.2 | 5.5 | **44.6** |
| **AT4G31950** | *CYP82C3* | 268.0 | 5.4 | **43.0** |
| **AT1G26240** | *Proline-rich extensin-like family protein* | 8584.4 | 5.4 | **42.4** |
| **AT4G22030** | *f box protein* | 153.1 | 5.4 | **41.4** |
| **AT2G43000** | *JUB1* | 4260.4 | 5.3 | **40.3** |
| **AT1G57630** | *Toll-Interleukin-Resistance (TIR) domain family protein* | 316.1 | 5.2 | **37.6** |
| **AT4G10540** | *Subtilase family protein* | 130.7 | 5.2 | **36.2** |
| **AT4G10530** | *Subtilase family protein* | 383.7 | 5.2 | **35.7** |
| **AT2G40740** | *WRKY55* | 162.0 | 5.1 | **33.9** |
| **AT5G39580** | *PER62* | 6987.6 | 5.0 | **32.7** |
| **AT5G24110** | *WRKY30* | 641.0 | 4.9 | **30.9** |
| **AT2G02010** | *GAD4* | 663.9 | 4.9 | **30.7** |
| **AT1G05880** | *ARI12* | 269.9 | 4.9 | **29.7** |
| **AT3G60270** | *Cupredoxin superfamily protein* | 1294.0 | 4.9 | **29.3** |
| **AT2G17740** | *VLG* | 116.7 | 4.8 | **27.7** |
| **AT5G27765** | *transmembrane protein* | 85.5 | 4.8 | **27.1** |
| **AT2G01379** | *?* | 98.2 | 4.7 | **26.8** |
| **AT1G14540** | *PER4* | 6319.8 | 4.7 | **26.6** |
| **AT1G05675** | *UDP-Glycosyltransferase superfamily protein* | 148.0 | 4.7 | **25.7** |
| **AT2G29100** | *GLR2.9* | 177.0 | 4.6 | **25.1** |
| **AT3G26830** | *CYP71B15* | 10737.4 | 4.6 | **24.7** |
| **AT3G55790** | *unknown* | 142.2 | 4.6 | **24.1** |
| **AT2G39400** | *MAGL6* | 2573.6 | 4.6 | **23.8** |
| **AT2G26560** | *PLP2* | 13719.0 | 4.6 | **23.6** |
| **AT5G26920** | *CBP60G* | 1645.7 | 4.5 | **23.4** |
| **AT5G11140** | *PEARLI 4-like* | 244.4 | 4.5 | **23.2** |
| **AT1G51920** | *unknown* | 256.9 | 4.5 | **23.1** |
| **AT2G25440** | *RLP20* | 213.4 | 4.5 | **22.9** |
| **AT1G14550** | *PER5* | 1138.6 | 4.5 | **22.4** |
| **AT5G52350** | *EXO70A3* | 94.7 | 4.5 | **22.3** |
| **AT1G51913** | *cryptdin protein-related* | 167.2 | 4.5 | **22.0** |
| **AT5G39150** | *RmlC-like cupins superfamily protein* | 1468.7 | 4.4 | **21.6** |
| **AT3G50160** | *DUF247* | 143.2 | 4.4 | **21.6** |
| **AT5G39180** | *RmlC-like cupins superfamily protein* | 1518.0 | 4.4 | **21.5** |
| **AT2G02930** | *GSTF3* | 4190.6 | 4.4 | **21.3** |
| **AT4G28460** | *unknown* | 342.6 | 4.4 | **21.1** |
| **AT3G02840** | *ARM repeat superfamily protein* | 248.1 | 4.4 | **21.1** |
| **AT4G37290** | *unknown* | 232.0 | 4.4 | **20.6** |
| **AT5G65600** | *LECRK92* | 1211.1 | 4.4 | **20.6** |
| **AT4G10520** | *Subtilase family protein* | 100.0 | 4.2 | **18.8** |
| **AT5G39120** | *RmlC-like cupins superfamily protein* | 779.6 | 4.2 | **18.8** |
| **AT1G63570** | *CRRSP7* | 100.7 | 4.2 | **18.6** |
| **AT2G36770** | *UDP-Glycosyltransferase superfamily protein* | 55.8 | 4.2 | **18.1** |
| **AT4G04480** | *F box protein* | 77.4 | 4.1 | **17.5** |
| **AT4G23030** | *MATE efflux family protein* | 173.9 | 4.1 | **17.1** |
| **AT1G51915** | *cryptdin protein-related* | 71.0 | 4.1 | **16.9** |
| **AT1G32350** | *AOX3* | 93.5 | 4.1 | **16.6** |
| **AT3G26200** | *CYP71B22* | 298.2 | 4.0 | **16.5** |
| **AT1G11300** | *EGM1* | 3531.3 | 4.0 | **16.5** |
| **AT1G26420** | *ATBBE7* | 2925.1 | 4.0 | **16.1** |
| **AT4G23250** | *CRK17* | 231.8 | 4.0 | **15.9** |
| **AT5G51465** | *?* | 48.1 | 4.0 | **15.7** |
| **AT3G53590** | *Leucine-rich repeat protein kinase family protein* | 128.6 | 4.0 | **15.6** |
| **AT5G47850** | *CCR4* | 101.8 | 4.0 | **15.5** |
| **AT5G23980** | *FRO4* | 744.7 | 3.9 | **15.5** |
| **AT1G67000** | *Protein kinase superfamily protein* | 489.2 | 3.9 | **15.1** |
| **AT2G15220** | *Plant basic secretory protein (BSP) family* | 21850.1 | 3.9 | **15.0** |
| **AT2G29110** | *GLR2.8* | 166.5 | 3.9 | **14.7** |
| **AT1G74190** | *ATRLP15* | 58.5 | 3.8 | **14.4** |
| **AT3G13433** | *unknown* | 46.0 | 3.8 | **14.3** |
| **AT5G44565** | *unknown* | 154.3 | 3.8 | **14.2** |
| **AT3G28930** | *AIG2A* | 1441.1 | 3.8 | **14.2** |
| **AT1G57650** | *disease resistance protein with ATP-binding domain* | 46.2 | 3.8 | **14.0** |
| **AT1G36622** | *unknown* | 717.2 | 3.8 | **13.9** |
| **AT2G18680** | *unknown* | 244.3 | 3.8 | **13.6** |
| **AT5G07760** | *FH21A* | 197.2 | 3.8 | **13.6** |
| **AT2G43570** | *CHI* | 16103.8 | 3.8 | **13.5** |
| **AT1G63590** | *CRRSP5* | 115.5 | 3.7 | **13.3** |
| **AT2G17040** | *NAC036* | 80.0 | 3.7 | **13.2** |
| **AT1G78410** | *VQ motif-containing protein* | 168.9 | 3.7 | **13.1** |
| **AT1G21240** | *WAK3* | 67.4 | 3.7 | **12.9** |
| **AT5G36970** | *NHL25* | 86.7 | 3.7 | **12.9** |
| **AT5G64905** | *PEP3* | 816.7 | 3.7 | **12.8** |
| **AT5G25260** | *SPFH/Band 7/PHB domain-containing membrane-associated protein family* | 279.8 | 3.7 | **12.8** |
| **AT2G29220** | *LECRK31* | 105.5 | 3.7 | **12.6** |
| **AT2G04040** | *DTX1* | 570.8 | 3.6 | **12.6** |
| **AT5G38900** | *Thioredoxin superfamily protein* | 3441.5 | 3.6 | **12.5** |
| **AT1G56240** | *PP2B13* | 98.3 | 3.6 | **12.5** |
| **AT5G22910** | *CHX9* | 50.0 | 3.6 | **12.5** |
| **AT1G22890** | *unknown* | 755.3 | 3.6 | **12.5** |
| **AT1G66090** | *Disease resistance protein (TIR-NBS class)* | 105.4 | 3.6 | **12.5** |
| **AT5G39130** | *RmlC-like cupins superfamily protein* | 332.1 | 3.6 | **12.4** |
| **AT1G21120** | *IGMT2* | 25625.7 | 3.6 | **12.2** |
| **AT2G23830** | *PVA31* | 161.5 | 3.6 | **12.1** |
| **AT4G22710** | *T12H17.100* | 6270.0 | 3.6 | **12.0** |
| **AT1G74080** | *MYB122* | 1127.2 | 3.5 | **11.6** |
| **AT4G14365** | *XBAT34* | 902.3 | 3.5 | **11.6** |
| **AT2G41100** | *CML12* | 4733.7 | 3.5 | **11.4** |
| **AT5G39030** | *Protein kinase superfamily protein* | 157.1 | 3.5 | **11.4** |
| **AT5G25250** | *FLOT1* | 2640.7 | 3.5 | **11.3** |
| **AT5G39160** | *RmlC-like cupins superfamily protein* | 724.0 | 3.5 | **11.1** |
| **AT5G39190** | *GLP5A* | 848.9 | 3.5 | **11.0** |
| **AT4G22690** | *CYP706A1* | 7714.5 | 3.4 | **10.8** |
| **AT1G66465** | *?* | 98.8 | 3.4 | **10.7** |
| **AT2G43620** | *Chitinase family protein* | 1159.7 | 3.4 | **10.6** |
| **AT2G38870** | *PR peptide* | 6779.2 | 3.4 | **10.5** |
| **AT1G47130** | *?* | 185.2 | 3.4 | **10.2** |
| **AT3G18250** | *Putative membrane lipoprotein* | 1202.4 | 3.3 | **10.1** |
| **AT4G23260** | *CRK18* | 357.0 | 3.3 | **10.0** |
| **AT5G54710** | *Ankyrin repeat family protein* | 250.8 | 3.3 | **10.0** |
| **AT4G04540** | *CRK39* | 55.0 | 3.3 | **9.9** |
| **AT4G00970** | *CRK41* | 36.4 | 3.3 | **9.9** |
| **AT4G11480** | *CRK32* | 208.9 | 3.3 | **9.8** |
| **AT3G21780** | *UGT71B6* | 199.9 | 3.3 | **9.8** |
| **AT4G04570** | *CRK40* | 699.9 | 3.3 | **9.7** |
| **AT1G75830** | *PDF1.1* | 46.4 | 3.3 | **9.7** |
| **AT2G43510** | *ATTI1* | 9149.2 | 3.3 | **9.6** |
| **AT2G14440** | *Leucine-rich repeat protein kinase family protein* | 312.3 | 3.3 | **9.6** |
| **AT1G06137** | *unknown* | 27.2 | 3.3 | **9.5** |
| **AT1G21230** | *WAK5* | 185.9 | 3.2 | **9.4** |
| **AT1G18570** | *MYB51* | 4294.1 | 3.2 | **9.4** |
| **AT4G26120** | *Ankyrin repeat family protein* | 1565.3 | 3.2 | **9.1** |
| **AT2G18690** | *unknown* | 5917.2 | 3.2 | **9.1** |
| **AT3G14225** | *GLIP4* | 724.4 | 3.2 | **9.1** |
| **AT5G37490** | *ARM repeat superfamily protein* | 133.8 | 3.2 | **9.0** |
| **AT3G23123** | *?* | 32.0 | 3.2 | **9.0** |
| **AT5G05730** | *ASA1* | 8576.7 | 3.2 | **9.0** |
| **AT3G28580** | *P-loop containing nucleoside triphosphate hydrolases superfamily protein* | 689.9 | 3.2 | **9.0** |
| **AT1G11610** | *CYP71A18* | 26.7 | 3.2 | **8.9** |
| **AT1G18970** | *GLP1* | 10724.2 | 3.2 | **8.9** |
| **AT1G36640** | *unknown* | 321.8 | 3.1 | **8.8** |
| **AT2G37810** | *Cysteine/Histidine-rich C1 domain family protein* | 25.8 | 3.1 | **8.8** |
| **AT5G03390** | *unknown* | 331.3 | 3.1 | **8.7** |
| **AT5G05300** | *IDL6* | 57.9 | 3.1 | **8.7** |
| **AT3G54150** | *EFD* | 1679.4 | 3.1 | **8.7** |
| **AT5G20230** | *BCB* | 852.2 | 3.1 | **8.7** |
| **AT1G01560** | *MPK11* | 191.7 | 3.1 | **8.5** |
| **AT5G11210** | *GLR2.5* | 1072.6 | 3.1 | **8.4** |
| **AT1G65500** | *unknown* | 249.2 | 3.1 | **8.3** |
| **AT1G44130** | *aspartyl protease family protein* | 101.3 | 3.0 | **8.3** |
| **AT5G39670** | *CML45* | 804.7 | 3.0 | **8.2** |
| **AT4G14450** | *unknown* | 56.1 | 3.0 | **8.2** |
| **AT1G69920** | *GSTU12* | 1529.6 | 3.0 | **8.2** |
| **AT2G31345** | *unknown* | 27.7 | 3.0 | **8.2** |
| **AT2G38860** | *DJ1E* | 28587.3 | 3.0 | **8.1** |
| **AT2G47240** | *LACS1* | 2514.1 | 3.0 | **8.1** |
| **AT4G18990** | *XTH29* | 59.4 | 3.0 | **8.1** |
| **AT5G22555** | *unknown* | 325.0 | 3.0 | **8.1** |
| **AT1G55230** | *unknown* | 63.6 | 3.0 | **8.1** |
| **AT4G11470** | *CRK31* | 357.3 | 3.0 | **8.0** |
| **AT1G64400** | *LACS3* | 4271.6 | 3.0 | **7.9** |
| **AT2G29120** | *GLR2.7* | 238.4 | 3.0 | **7.9** |
| **AT5G17990** | *PAT1* | 9037.3 | 3.0 | **7.9** |
| **AT1G65800** | *ARK2* | 93.1 | 3.0 | **7.8** |
| **AT2G19190** | *FRK1* | 298.1 | 3.0 | **7.7** |
| **AT3G21305** | *?* | 141.7 | 2.9 | **7.6** |
| **AT3G60140** | *BGLU30* | 2691.5 | 2.9 | **7.6** |
| **AT5G44590** | *S-adenosyl-L-methionine-dependent methyltransferases superfamily protein* | 76.3 | 2.9 | **7.6** |
| **AT1G36623** | *?* | 22.0 | 2.9 | **7.6** |
| **AT2G40180** | *PP2C5* | 192.9 | 2.9 | **7.6** |
| **AT1G53625** | *unknown* | 669.1 | 2.9 | **7.5** |
| **AT4G23320** | *CRK24* | 29.1 | 2.9 | **7.5** |
| **AT1G21130** | *IGMT4* | 18281.2 | 2.9 | **7.5** |
| **AT1G21550** | *CML44* | 197.2 | 2.9 | **7.5** |
| **AT5G44567** | *?* | 464.3 | 2.9 | **7.4** |
| **AT5G52720** | *Copper transport protein family* | 70.9 | 2.9 | **7.4** |
| **AT5G45240** | *Disease resistance protein* | 205.7 | 2.9 | **7.4** |
| **AT4G39830** | *Cupredoxin superfamily protein* | 762.5 | 2.9 | **7.4** |
| **AT4G31960** | *unknown* | 40.0 | 2.9 | **7.3** |
| **AT1G02940** | *GSTF5* | 21.4 | 2.9 | **7.3** |
| **AT4G23170** | *CRK9* | 208.0 | 2.9 | **7.3** |
| **AT1G33600** | *Leucine-rich repeat (LRR) family protein* | 637.4 | 2.9 | **7.3** |
| **AT1G67270** | *Zinc-finger domain of monoamine-oxidase A repressor R1 protein* | 213.7 | 2.9 | **7.3** |
| **AT4G18170** | *WRKY28* | 542.5 | 2.9 | **7.2** |
| **AT4G21680** | *NPF7.2* | 1232.9 | 2.8 | **7.2** |
| **AT1G33030** | *O-methyltransferase family protein* | 469.3 | 2.8 | **7.2** |
| **AT1G48980** | *2-oxoglutarate (2OG) and Fe(II)-dependent oxygenase superfamily protein* | 24.8 | 2.8 | **7.2** |
| **AT1G69930** | *GSTU11* | 116.2 | 2.8 | **7.1** |
| **AT1G64160** | *DIR5* | 654.6 | 2.8 | **7.1** |
| **AT5G45090** | *PP2A7* | 395.7 | 2.8 | **7.0** |
| **AT5G22300** | *NIT4* | 480.8 | 2.8 | **7.0** |
| **AT2G19500** | *CKX2* | 116.6 | 2.8 | **6.9** |
| **AT5G64120** | *PER71* | 45175.5 | 2.8 | **6.9** |
| **AT1G09930** | *OPT2* | 33.4 | 2.8 | **6.9** |
| **AT5G42830** | *HXXXD-type acyl-transferase family protein* | 3965.5 | 2.8 | **6.8** |
| **AT2G29460** | *GSTU4* | 646.7 | 2.8 | **6.7** |
| **AT1G03670** | *Ankyrin repeat containing protein* | 24.1 | 2.7 | **6.7** |
| **AT5G01540** | *LECRK62* | 243.6 | 2.7 | **6.7** |
| **AT1G53970** | *unknown* | 92.8 | 2.7 | **6.7** |
| **AT3G54640** | *TSA1* | 26231.5 | 2.7 | **6.6** |
| **AT3G48850** | *MPT2* | 785.0 | 2.7 | **6.6** |
| **AT1G33900** | *IAN4* | 62.2 | 2.7 | **6.6** |
| **AT4G39610** | *unknown* | 28.7 | 2.7 | **6.5** |
| **AT3G44840** | *S-adenosyl-L-methionine-dependent methyltransferases superfamily protein* | 43.2 | 2.7 | **6.5** |
| **AT1G24140** | *3MMP* | 297.1 | 2.7 | **6.5** |
| **AT1G13520** | *unknown* | 3457.0 | 2.7 | **6.4** |
| **AT1G47890** | *ATRLP7* | 660.5 | 2.7 | **6.4** |
| **AT5G54720** | *Ankyrin repeat family protein* | 67.3 | 2.7 | **6.4** |
| **AT4G13090** | *XTH2* | 34.1 | 2.7 | **6.4** |
| **AT4G18195** | *PUP8* | 86.6 | 2.7 | **6.4** |
| **AT5G19700** | *DTX52* | 31.0 | 2.7 | **6.3** |
| **AT4G14630** | *GLP9* | 4897.3 | 2.7 | **6.3** |
| **AT2G43590** | *Chitinase family protein* | 49624.4 | 2.7 | **6.3** |
| **AT3G12900** | *2-oxoglutarate (2OG) and Fe(II)-dependent oxygenase superfamily protein* | 38.0 | 2.7 | **6.3** |
| **AT5G22520** | *unknown* | 31.4 | 2.6 | **6.3** |
| **AT4G11521** | *CRK34* | 30.1 | 2.6 | **6.3** |
| **AT4G40020** | *Myosin heavy chain-related protein* | 357.2 | 2.6 | **6.2** |
| **AT4G16820** | *DAD1-LIKE LIPASE 1* | 54.3 | 2.6 | **6.2** |
| **AT1G19610** | *PDF1.4* | 502.8 | 2.6 | **6.1** |
| **AT2G24180** | *CYP71B6* | 27905.4 | 2.6 | **6.1** |
| **AT3G03670** | *PER28* | 2407.9 | 2.6 | **6.1** |
| **AT4G13095** | *LCR37* | 24.8 | 2.6 | **6.0** |
| **AT4G23810** | *WRKY53* | 888.1 | 2.6 | **5.9** |
| **AT1G65790** | *ARK1* | 24.8 | 2.6 | **5.9** |
| **AT1G33910** | *P-loop containing nucleoside triphosphate hydrolases superfamily protein* | 25.1 | 2.6 | **5.9** |
| **AT4G37060** | *PLP5* | 351.5 | 2.6 | **5.9** |
| **AT5G41750** | *Disease resistance protein (TIR-NBS-LRR class) family* | 1075.8 | 2.6 | **5.9** |
| **AT1G15625** | *unknown* | 682.8 | 2.5 | **5.8** |
| **AT5G39110** | *RmlC-like cupins superfamily protein* | 1765.2 | 2.5 | **5.8** |
| **AT1G53980** | *Ubiquitin-like superfamily protein* | 239.2 | 2.5 | **5.8** |
| **AT3G23250** | *MYB15* | 669.6 | 2.5 | **5.7** |
| **AT1G28040** | *RING/U-box superfamily protein* | 31.9 | 2.5 | **5.7** |
| **AT2G23260** | *UGT84B1* | 18.0 | 2.5 | **5.7** |
| **AT1G08630** | *THA1* | 430.5 | 2.5 | **5.6** |
| **AT1G09932** | *Phosphoglycerate mutase family protein* | 701.0 | 2.5 | **5.6** |
| **AT5G53990** | *UGT79B9* | 2000.2 | 2.5 | **5.6** |
| **AT1G09080** | *BIP3* | 39.1 | 2.5 | **5.5** |
| **AT3G47250** | *unknown* | 84.6 | 2.5 | **5.5** |
| **AT1G06135** | *unknown* | 14.9 | 2.5 | **5.5** |
| **AT1G61550** | *S-locus lectin protein kinase family protein* | 333.3 | 2.5 | **5.5** |
| **AT1G51620** | *Protein kinase superfamily protein* | 326.7 | 2.5 | **5.5** |
| **AT4G32950** | *Protein phosphatase 2C family protein* | 269.7 | 2.4 | **5.4** |
| **AT1G09176** | *unknown* | 70.9 | 2.4 | **5.4** |
| **AT1G73805** | *SARD1* | 173.2 | 2.4 | **5.4** |
| **AT1G56250** | *PP2-B14* | 41.9 | 2.4 | **5.4** |
| **AT2G28210** | *ACA2* | 1369.1 | 2.4 | **5.4** |
| **AT1G55450** | *S-adenosyl-L-methionine-dependent methyltransferases superfamily protein* | 7618.3 | 2.4 | **5.4** |
| **AT1G75000** | *GNS1/SUR4 membrane protein family* | 1973.0 | 2.4 | **5.3** |
| **AT5G46350** | *WRKY8* | 385.4 | 2.4 | **5.3** |
| **AT5G49780** | *Leucine-rich repeat protein kinase family protein* | 2033.0 | 2.4 | **5.3** |
| **AT2G03410** | *Mo25 family protein* | 44.2 | 2.4 | **5.2** |
| **AT1G21850** | *sks8* | 21.1 | 2.4 | **5.2** |
| **AT2G40250** | *SGNH hydrolase-type esterase superfamily protein* | 87.8 | 2.4 | **5.2** |
| **AT4G23180** | *CRK10* | 686.2 | 2.4 | **5.2** |
| **AT3G63380** | *ACA12* | 4113.7 | 2.4 | **5.2** |
| **AT1G13480** | *unknown* | 716.4 | 2.4 | **5.2** |
| **AT3G48840** | *RNA-binding (RRM/RBD/RNP motifs) family protein* | 88.0 | 2.4 | **5.2** |
| **AT2G43890** | *Pectin lyase-like superfamily protein* | 179.1 | 2.3 | **5.0** |
| **AT5G48400** | *GLR1.2* | 985.4 | 2.3 | **5.0** |
| **AT5G67310** | *CYP81G1* | 55.9 | 2.3 | **5.0** |
| **AT1G21110** | *IGMT3* | 28018.0 | 2.3 | **5.0** |
| **AT5G52670** | *Copper transport protein family* | 48.7 | 2.3 | **5.0** |
| **AT3G45060** | *NRT2.6* | 918.6 | 2.3 | **4.9** |
| **AT4G39580** | *Galactose oxidase/kelch repeat superfamily protein* | 463.3 | 2.3 | **4.9** |
| **AT4G13300** | *ATTPS13* | 748.7 | 2.3 | **4.9** |
| **AT4G14368** | *Regulator of chromosome condensation (RCC1) family protein* | 117.7 | 2.3 | **4.9** |
| **AT4G39950** | *CYP79B2* | 34341.7 | 2.3 | **4.9** |
| **AT1G42980** | *Actin-binding FH2 (formin homology 2) family protein* | 53.5 | 2.3 | **4.9** |
| **AT3G15518** | *unknown* | 631.6 | 2.3 | **4.9** |
| **AT1G17420** | *LOX3* | 18.5 | 2.3 | **4.9** |
| **AT1G59865** | *unknown* | 32.1 | 2.3 | **4.9** |
| **AT4G10500** | *DLO1* | 1550.2 | 2.3 | **4.9** |
| **AT1G30730** | *ATBBE11* | 13168.7 | 2.3 | **4.9** |
| **AT3G26820** | *Esterase/lipase/thioesterase family protein* | 150.4 | 2.3 | **4.9** |
| **AT2G25410** | *RING/U-box superfamily protein* | 281.1 | 2.3 | **4.9** |
| **AT3G01500** | *BCA1* | 14.6 | 2.3 | **4.8** |
| **AT2G03260** | *PHO1-H2* | 641.9 | 2.3 | **4.8** |
| **AT5G22530** | *unknown* | 58.1 | 2.3 | **4.8** |
| **AT1G25220** | *ASB1* | 7528.1 | 2.3 | **4.8** |
| **AT2G31865** | *PARG2* | 1395.3 | 2.3 | **4.8** |
| **AT1G77210** | *STP14* | 377.1 | 2.3 | **4.8** |
| **AT1G11303** | *unknown* | 24.6 | 2.3 | **4.8** |
| **AT1G24807** | *Glutamine amidotransferase type 1 family protein* | 886.6 | 2.2 | **4.7** |
| **AT1G25083** | *Glutamine amidotransferase type 1 family protein* | 886.6 | 2.2 | **4.7** |
| **AT5G40010** | *AATP1* | 611.4 | 2.2 | **4.7** |
| **AT5G35525** | *PCR3* | 159.3 | 2.2 | **4.7** |
| **AT4G33050** | *IQM1* | 4619.4 | 2.2 | **4.7** |
| **AT1G63580** | *CRRSP6* | 356.4 | 2.2 | **4.7** |
| **AT3G19620** | *BXL5* | 48.5 | 2.2 | **4.7** |
| **AT4G26260** | *MIOX4* | 1213.6 | 2.2 | **4.7** |
| **AT1G74590** | *GSTU10* | 3936.1 | 2.2 | **4.7** |
| **AT5G02780** | *GSTL1* | 1651.0 | 2.2 | **4.6** |
| **AT1G54950** | *unknown* | 85.8 | 2.2 | **4.6** |
| **AT3G26230** | *CYP71B24* | 501.8 | 2.2 | **4.6** |
| **AT2G29250** | *LECRK32* | 21.0 | 2.2 | **4.6** |
| **AT1G21400** | *Thiamin diphosphate-binding fold (THDP-binding) superfamily protein* | 1704.1 | 2.2 | **4.6** |
| **AT1G24909** | *Glutamine amidotransferase type 1 family protein* | 1832.2 | 2.2 | **4.6** |
| **AT2G24195** | *unknown* | 20.3 | 2.2 | **4.6** |
| **AT4G37370** | *CYP81D8* | 696.1 | 2.2 | **4.6** |
| **AT1G25155** | *Glutamine amidotransferase type 1 family protein* | 901.3 | 2.2 | **4.6** |
| **AT5G44585** | *unknown* | 2360.3 | 2.2 | **4.5** |
| **AT5G16330** | *NC domain-containing protein-related* | 24.8 | 2.2 | **4.5** |
| **AT2G15080** | *RLP19* | 36.2 | 2.2 | **4.5** |
| **AT5G39490** | *F-box family protein* | 30.0 | 2.2 | **4.5** |
| **AT2G25735** | *unknown* | 455.2 | 2.2 | **4.5** |
| **AT5G47220** | *ERF2* | 114.4 | 2.2 | **4.5** |
| **AT4G29050** | *LECRK59* | 541.0 | 2.2 | **4.5** |
| **AT3G59080** | *aspartyl protease family protein* | 1403.8 | 2.2 | **4.4** |
| **AT4G02520** | *GSTF2* | 48808.8 | 2.1 | **4.4** |
| **AT2G37430** | *ZAT11* | 591.8 | 2.1 | **4.4** |
| **AT2G04070** | *DTX4* | 40.8 | 2.1 | **4.4** |
| **AT4G01010** | *CNGC13* | 837.8 | 2.1 | **4.4** |
| **AT3G11080** | *ATRLP35* | 1310.0 | 2.1 | **4.4** |
| **AT1G58420** | *Uncharacterised conserved protein* | 159.7 | 2.1 | **4.4** |
| **AT2G45550** | *CYP76C4* | 63.5 | 2.1 | **4.4** |
| **AT1G74360** | *Leucine-rich repeat protein kinase family protein* | 2524.3 | 2.1 | **4.4** |
| **AT4G35110** | *phospholipase-like protein (PEARLI 4)* | 2194.7 | 2.1 | **4.3** |
| **AT1G33870** | *P-loop containing nucleoside triphosphate hydrolases superfamily protein* | 137.6 | 2.1 | **4.3** |
| **AT4G02330** | *PME41* | 1173.9 | 2.1 | **4.3** |
| **AT1G53990** | *GLIP3* | 1243.7 | 2.1 | **4.3** |
| **AT1G63550** | *CRRSP9* | 72.7 | 2.1 | **4.3** |
| **AT4G23190** | *CRK11* | 3866.7 | 2.1 | **4.3** |
| **AT3G47480** | *CML47* | 31.9 | 2.1 | **4.3** |
| **AT3G09405** | *PAE4* | 913.3 | 2.1 | **4.3** |
| **AT1G26250** | *Proline-rich extensin-like family protein* | 1241.3 | 2.1 | **4.3** |
| **AT5G44690** | *unknown* | 17.6 | 2.1 | **4.3** |
| **AT1G61440** | *S-locus lectin protein kinase family protein* | 75.1 | 2.1 | **4.3** |
| **AT1G65484** | *unknown* | 240.1 | 2.1 | **4.2** |
| **AT3G19615** | *unknown* | 65.4 | 2.1 | **4.2** |
| **AT1G72520** | *LOX4* | 152.7 | 2.1 | **4.2** |
| **AT5G64890** | *PEP2* | 102.2 | 2.1 | **4.2** |
| **AT5G34780** | *KPR* | 42.1 | 2.1 | **4.2** |
| **AT2G29150** | *NAD(P)-binding Rossmann-fold superfamily protein* | 12.4 | 2.1 | **4.2** |
| **AT5G44568** | *unknown* | 74.5 | 2.1 | **4.2** |
| **AT2G26530** | *AR781* | 2703.4 | 2.1 | **4.2** |
| **AT1G07160** | *Protein phosphatase 2C family protein* | 156.6 | 2.1 | **4.2** |
| **AT5G06730** | *PER54* | 1360.9 | 2.1 | **4.2** |
| **AT4G28350** | *LECRK72* | 1240.4 | 2.0 | **4.1** |
| **AT2G24600** | *Ankyrin repeat family protein* | 320.5 | 2.0 | **4.1** |
| **AT1G02580** | *MEA* | 175.2 | 2.0 | **4.1** |
| **AT2G28755** | *UDP-D-glucuronate carboxy-lyase-related* | 112.1 | 2.0 | **4.1** |
| **AT1G21520** | *unknown* | 231.2 | 2.0 | **4.1** |
| **AT5G49770** | *Leucine-rich repeat protein kinase family protein* | 4077.6 | 2.0 | **4.1** |
| **AT2G23680** | *Cold acclimation protein WCOR413 family* | 1029.2 | 2.0 | **4.1** |
| **AT1G71140** | *DTX14* | 100.6 | 2.0 | **4.1** |
| **AT3G16150** | *N-terminal nucleophile aminohydrolases (Ntn hydrolases) superfamily protein* | 1113.6 | 2.0 | **4.1** |
| **AT3G55840** | *HSPRO1* | 517.1 | 2.0 | **4.1** |
| **AT1G63750** | *Disease resistance protein (TIR-NBS-LRR class) family* | 338.6 | 2.0 | **4.1** |
| **AT1G28190** | *unknown* | 1176.3 | 2.0 | **4.1** |
| **AT5G65980** | *PILS7* | 634.5 | 2.0 | **4.1** |
| **AT4G23610** | *Late embryogenesis abundant (LEA) hydroxyproline-rich glycoprotein family* | 50.5 | 2.0 | **4.0** |
| **AT3G14620** | *CYP72A8* | 1811.0 | 2.0 | **4.0** |
| **AT3G13610** | *2-oxoglutarate (2OG) and Fe(II)-dependent oxygenase superfamily protein* | 17573.1 | 2.0 | **4.0** |
| **AT5G48540** | *CRRSP55* | 2400.5 | 2.0 | **4.0** |
| **AT3G21520** | *DMP1* | 315.3 | 2.0 | **4.0** |
| **AT5G24550** | *BGLU32* | 17.1 | 2.0 | **4.0** |
| **AT1G32960** | *SBT3.3* | 65.9 | 2.0 | **4.0** |
| **AT4G15975** | *ATL17* | 109.1 | 2.0 | **3.9** |
| **AT2G43920** | *HOL2* | 8395.3 | 2.0 | **3.9** |
| **AT2G04400** | *IGPS* | 7865.9 | 2.0 | **3.9** |
| **AT1G67520** | *lectin protein kinase family protein* | 924.3 | 2.0 | **3.9** |
| **AT5G63250** | *Carbohydrate-binding X8 domain superfamily protein* | 38.2 | 2.0 | **3.9** |
| **AT5G52760** | *HIPP14* | 26.3 | 2.0 | **3.9** |
| **AT4G10510** | *Subtilase family protein* | 878.3 | 2.0 | **3.9** |
| **AT3G56710** | *SIB1* | 313.4 | 2.0 | **3.9** |
| **AT3G23570** | *alpha/beta-Hydrolases superfamily protein* | 4238.6 | 2.0 | **3.9** |
| **AT5G24240** | *phosphatidylinositol 4-kinase gamma-like protein* | 996.1 | 1.9 | **3.9** |
| **AT5G65500** | *PUB50* | 286.6 | 1.9 | **3.9** |
| **AT1G33880** | *IAN2* | 58.0 | 1.9 | **3.9** |
| **AT4G23220** | *CRK14* | 15.0 | 1.9 | **3.8** |
| **AT3G44300** | *NIT2* | 14919.1 | 1.9 | **3.8** |
| **AT1G58320** | *PCR9* | 662.5 | 1.9 | **3.8** |
| **AT2G45130** | *SPX3* | 42.5 | 1.9 | **3.8** |
| **AT5G40590** | *Cysteine/Histidine-rich C1 domain family protein* | 1164.2 | 1.9 | **3.8** |
| **AT5G39630** | *Vesicle transport v-SNARE family protein* | 11.0 | 1.9 | **3.8** |
| **AT3G53160** | *UGT73C7* | 313.7 | 1.9 | **3.8** |
| **AT5G40000** | *P-loop containing nucleoside triphosphate hydrolase* | 87.5 | 1.9 | **3.8** |
| **AT3G23230** | *ERF098* | 29.1 | 1.9 | **3.7** |
| **AT1G21326** | *VQ motif-containing protein* | 111.2 | 1.9 | **3.7** |
| **AT3G60415** | *phosphoglycerate mutase family protein* | 35.2 | 1.9 | **3.7** |
| **AT5G24180** | *Lipase class 3-related protein* | 17.9 | 1.9 | **3.7** |
| **AT1G17990** | *FMN-linked oxidoreductases superfamily protein* | 987.8 | 1.9 | **3.7** |
| **AT1G13550** | *DUF1262* | 201.5 | 1.9 | **3.7** |
| **AT1G18020** | *FMN-linked oxidoreductases superfamily protein* | 987.5 | 1.9 | **3.7** |
| **AT3G46080** | *ZAT8* | 71.3 | 1.9 | **3.7** |
| **AT4G10860** | *unknown* | 27.3 | 1.9 | **3.7** |
| **AT3G23120** | *RLP38* | 34.8 | 1.9 | **3.7** |
| **AT5G46295** | *transmembrane protein* | 206.8 | 1.9 | **3.7** |
| **AT1G61720** | *BAN* | 13.3 | 1.9 | **3.7** |
| **AT4G19810** | *Glycosyl hydrolase family protein with chitinase insertion domain-containing protein* | 6295.5 | 1.9 | **3.7** |
| **AT5G48430** | *aspartyl protease family protein* | 8737.5 | 1.9 | **3.7** |
| **AT2G44578** | *RING/U-box superfamily protein* | 92.3 | 1.9 | **3.7** |
| **AT3G47380** | *Plant invertase/pectin methylesterase inhibitor superfamily protein* | 547.7 | 1.9 | **3.6** |
| **AT1G29860** | *WRKY71* | 89.8 | 1.9 | **3.6** |
| **AT2G45450** | *ZPR1* | 321.7 | 1.9 | **3.6** |
| **AT1G16225** | *Target SNARE coiled-coil domain protein* | 60.9 | 1.9 | **3.6** |
| **AT1G64010** | *Serine protease inhibitor (SERPIN) family protein* | 31.7 | 1.9 | **3.6** |
| **AT5G60780** | *NRT2.3* | 10.5 | 1.9 | **3.6** |
| **AT1G66160** | *PUB20* | 1602.9 | 1.9 | **3.6** |
| **AT2G30660** | *ATP-dependent caseinolytic (Clp) protease/crotonase family protein* | 16.1 | 1.8 | **3.6** |
| **AT2G38470** | *WRKY33* | 8491.2 | 1.8 | **3.6** |
| **AT2G04495** | *transmembrane protein* | 20.2 | 1.8 | **3.6** |
| **AT3G61280** | *O-glucosyltransferase rumi-like protein* | 265.2 | 1.8 | **3.6** |
| **AT5G43650** | *BHLH92* | 11.5 | 1.8 | **3.6** |
| **AT1G13490** | *unknown* | 31.7 | 1.8 | **3.6** |
| **AT5G54810** | *TSB1* | 22846.9 | 1.8 | **3.6** |
| **AT2G29470** | *GSTU3* | 15.3 | 1.8 | **3.6** |
| **AT2G36780** | *UGT73C3* | 10.8 | 1.8 | **3.6** |
| **AT1G44010** | *transmembrane protein* | 11.9 | 1.8 | **3.5** |
| **AT1G53620** | *transmembrane protein* | 67.9 | 1.8 | **3.5** |
| **AT5G37450** | *Leucine-rich repeat protein kinase family protein* | 1500.9 | 1.8 | **3.5** |
| **AT5G13320** | *GH3.12* | 41.6 | 1.8 | **3.5** |
| **AT5G09730** | *BXL3* | 29.9 | 1.8 | **3.5** |
| **AT2G37770** | *AKR4C9* | 29.7 | 1.8 | **3.5** |
| **AT5G05340** | *PER52* | 73.2 | 1.8 | **3.5** |
| **AT2G33160** | *glycoside hydrolase family 28 protein* | 24.5 | 1.8 | **3.4** |
| **AT1G21250** | *WAK1* | 53.3 | 1.8 | **3.4** |
| **AT4G39940** | *APK2* | 10234.2 | 1.8 | **3.4** |
| **AT4G14370** | *dl3225c* | 1073.5 | 1.8 | **3.4** |
| **AT5G22270** | *unknown* | 117.0 | 1.8 | **3.4** |
| **AT4G27070** | *TSB2* | 1655.6 | 1.8 | **3.4** |
| **AT5G60350** | *unknown* | 65.3 | 1.8 | **3.4** |
| **AT5G66780** | *late embryogenesis abundant protein* | 120.2 | 1.8 | **3.4** |
| **AT1G61420** | *S-locus lectin protein kinase family protein* | 931.1 | 1.8 | **3.4** |
| **AT4G07820** | *PR1-related* | 7602.9 | 1.7 | **3.4** |
| **AT1G24270** | *unknown* | 54.4 | 1.7 | **3.4** |
| **AT3G60540** | *Preprotein translocase Sec, Sec61-beta subunit protein* | 166.7 | 1.7 | **3.4** |
| **AT5G40780** | *LHT1* | 6057.3 | 1.7 | **3.3** |
| **AT4G08380** | *Proline-rich extensin-like family protei* | 219.1 | 1.7 | **3.3** |
| **AT4G11070** | *WRKY41* | 390.2 | 1.7 | **3.3** |
| **AT3G51340** | *aspartyl protease family protein* | 690.4 | 1.7 | **3.3** |
| **AT4G11000** | *Ankyrin repeat family protein* | 26.4 | 1.7 | **3.3** |
| **AT1G68620** | *CXE6* | 5556.2 | 1.7 | **3.3** |
| **AT2G13275** | *unknown* | 34.0 | 1.7 | **3.3** |
| **AT3G51450** | *SSL7* | 838.6 | 1.7 | **3.3** |
| **AT1G33890** | *IAN3* | 122.1 | 1.7 | **3.3** |
| **AT3G09520** | *exocyst subunit exo70 family protein H4* | 125.5 | 1.7 | **3.3** |
| **AT1G01680** | *PUB54* | 24.1 | 1.7 | **3.3** |
| **AT1G19250** | *FMO1* | 470.6 | 1.7 | **3.3** |
| **AT1G13530** | *unknown* | 968.3 | 1.7 | **3.3** |
| **AT1G49570** | *PER10* | 745.2 | 1.7 | **3.3** |
| **AT5G64110** | *PER70* | 933.6 | 1.7 | **3.3** |
| **AT1G08100** | *NRT2.2* | 346.9 | 1.7 | **3.3** |
| **AT3G19920** | *BTB/POZ domain protein* | 29.9 | 1.7 | **3.3** |
| **AT1G51890** | *Leucine-rich repeat protein kinase family protein* | 5728.6 | 1.7 | **3.3** |
| **AT1G67470** | *Protein kinase superfamily protein* | 274.9 | 1.7 | **3.3** |
| **AT1G65481** | *transmembrane protein* | 105.9 | 1.7 | **3.2** |
| **AT2G45760** | *BAP2* | 60.6 | 1.7 | **3.2** |
| **AT3G45730** | *unknown* | 977.1 | 1.7 | **3.2** |
| **AT4G26560** | *CBL7* | 88.3 | 1.7 | **3.2** |
| **AT1G53610** | *transmembrane protein* | 308.1 | 1.7 | **3.2** |
| **AT5G27420** | *ATL31* | 4047.8 | 1.7 | **3.2** |
| **AT3G50900** | *unknown* | 1319.5 | 1.7 | **3.2** |
| **AT2G29340** | *NAD-dependent epimerase/dehydratase family protein* | 2592.2 | 1.7 | **3.2** |
| **AT2G46495** | *ATL21A* | 812.4 | 1.7 | **3.2** |
| **AT4G10720** | *Ankyrin repeat family protein* | 540.5 | 1.7 | **3.2** |
| **AT1G53950** | *ubiquitin-40S ribosomal S27a-like protein* | 408.7 | 1.7 | **3.2** |
| **AT4G15530** | *PPDK* | 1858.7 | 1.7 | **3.2** |
| **AT1G74140** | *RBL17* | 10.8 | 1.7 | **3.2** |
| **AT3G46260** | *kinase-like protein* | 23.0 | 1.7 | **3.2** |
| **AT5G10380** | *ATL55* | 50.9 | 1.7 | **3.2** |
| **AT5G38910** | *RmlC-like cupins superfamily protein* | 1007.0 | 1.7 | **3.2** |
| **AT1G23840** | *transmembrane protein* | 96.7 | 1.7 | **3.2** |
| **AT1G35210** | *unknown* | 216.6 | 1.7 | **3.2** |
| **AT1G49000** | *transmembrane protein* | 563.9 | 1.7 | **3.2** |
| **AT4G23700** | *CHX17* | 9398.3 | 1.7 | **3.1** |
| **AT4G19370** | *chitin synthase, putative* | 806.0 | 1.7 | **3.1** |
| **AT4G18250** | *receptor Serine/Threonine kinase-like protein* | 1062.0 | 1.7 | **3.1** |
| **AT3G49620** | *DIN11* | 18.5 | 1.7 | **3.1** |
| **AT3G01970** | *WRKY45* | 1392.3 | 1.7 | **3.1** |
| **AT5G42380** | *CML37* | 148.8 | 1.6 | **3.1** |
| **AT1G64710** | *GroES-like zinc-binding alcohol dehydrogenase family protein* | 12.3 | 1.6 | **3.1** |
| **AT5G57890** | *ASB2* | 1710.2 | 1.6 | **3.1** |
| **AT1G72900** | *Toll-Interleukin-Resistance (TIR) domain-containing protein* | 1197.8 | 1.6 | **3.1** |
| **AT1G56060** | *cysteine-rich/transmembrane domain protein B* | 10.5 | 1.6 | **3.1** |
| **AT3G19930** | *STP4* | 23953.4 | 1.6 | **3.1** |
| **AT3G17700** | *CNGC20* | 1230.5 | 1.6 | **3.1** |
| **AT5G36925** | *unknown* | 21.8 | 1.6 | **3.1** |
| **AT3G23122** | *unknown* | 8.2 | 1.6 | **3.1** |
| **AT4G17490** | *ERF6* | 2423.1 | 1.6 | **3.1** |
| **AT1G61560** | *MLO6* | 6448.3 | 1.6 | **3.1** |
| **AT4G21920** | *unknown* | 37.4 | 1.6 | **3.1** |
| **AT5G45340** | *CYP707A3* | 928.9 | 1.6 | **3.0** |
| **AT1G58225** | *unknown* | 10.2 | 1.6 | **3.0** |
| **AT4G19975** | *nucleotide-diphospho-sugar transferase family protein* | 702.0 | 1.6 | **3.0** |
| **AT1G47603** | *PUP19* | 155.4 | 1.6 | **3.0** |
| **AT1G72920** | *Toll-Interleukin-Resistance (TIR) domain family protein* | 142.9 | 1.6 | **3.0** |
| **AT4G01630** | *EXPA17* | 1426.2 | 1.6 | **3.0** |

**Table S2.** List of down-regulated plant genes in *A. thaliana* (ATH) and *V. dahliae* (VDA) co-culture compared to *A. thaliana* grown separately. Cut-off was made at a log_2_-fold change of -1.33.

| **GeneID** | **Symbol** | **base Mean** | **log2 Fold Change** | **Fold Change** |
| --- | --- | --- | --- | --- |
| **AT3G48740** | *SWEET11* | 3067.60 | -3.28 | **-9.70** |
| **AT2G33790** | *AGP30* | 723.31 | -3.01 | **-8.08** |
| **AT5G53190** | *SWEET3* | 40.76 | -2.77 | **-6.81** |
| **AT3G04330** | *Kunitz family trypsin and protease inhibitor protein* | 141.81 | -2.61 | **-6.10** |
| **AT3G19430** | *LEA protein-related* | 545.06 | -2.58 | **-5.99** |
| **AT4G28530** | *ANAC074* | 351.08 | -2.39 | **-5.24** |
| **AT4G26880** | *Stigma-specific Stig1 family protein* | 31.70 | -2.38 | **-5.19** |
| **AT4G12543** | *unknown* | 811.61 | -2.25 | **-4.76** |
| **AT3G17130** | *Plant invertase/pectin methylesterase inhibitor superfamily protein* | 37.29 | -2.07 | **-4.20** |
| **AT1G19960** | *transmembrane receptor* | 1322.15 | -2.04 | **-4.11** |
| **AT1G73120** | *unknown* | 81.93 | -2.04 | **-4.11** |
| **AT2G22750** | *BHLH18* | 70.60 | -2.02 | **-4.05** |
| **AT5G47450** | *TIP2-3* | 2474.18 | -1.98 | **-3.96** |
| **AT4G27400** | *(LEA) protein-related* | 430.87 | -1.97 | **-3.91** |
| **AT4G12555** | *unknown* | 383.02 | -1.96 | **-3.89** |
| **AT5G54370** | *(LEA) protein-related* | 3880.46 | -1.96 | **-3.89** |
| **AT3G53980** | *Bifunctional inhibitor/lipid-transfer protein* | 6281.40 | -1.89 | **-3.71** |
| **AT1G65570** | *RCPG* | 367.42 | -1.88 | **-3.69** |
| **AT5G60060** | *F-box family protein* | 102.22 | -1.88 | **-3.68** |
| **AT4G37700** | *unknown* | 1246.15 | -1.87 | **-3.67** |
| **AT2G32530** | *CSLB3* | 171.94 | -1.87 | **-3.66** |
| **AT4G12170** | *thioredoxin family protein* | 50.23 | -1.87 | **-3.66** |
| **AT2G34430** | *Lhb1B1* | 220.01 | -1.84 | **-3.58** |
| **AT5G23660** | *SWEET12* | 4167.10 | -1.84 | **-3.58** |
| **AT2G34420** | *Lhb1B2* | 2455.70 | -1.84 | **-3.57** |
| **AT4G37160** | *SKS15* | 752.34 | -1.83 | **-3.56** |
| **AT1G64380** | *ERF061* | 458.20 | -1.83 | **-3.56** |
| **AT4G12480** | *EARLI1* | 2010.44 | -1.81 | **-3.50** |
| **AT2G01520** | *MLP328* | 53908.08 | -1.79 | **-3.45** |
| **AT1G45015** | *MD-2-related lipid recognition domain-containing protein;* | 772.51 | -1.78 | **-3.44** |
| **AT5G07990** | *CYP75B1* | 1331.59 | -1.78 | **-3.44** |
| **AT2G19990** | *PR-1-LIKE* | 285.27 | -1.78 | **-3.43** |
| **AT5G55110** | *Stigma-specific Stig1 family protein* | 192.86 | -1.77 | **-3.40** |
| **AT3G27250** | *unknown* | 39.40 | -1.76 | **-3.39** |
| **AT4G17340** | *TIP2-2* | 11371.19 | -1.76 | **-3.38** |
| **AT2G02450** | *NAC035* | 144.54 | -1.75 | **-3.37** |
| **AT5G62330** | *Plant invertase/pectin methylesterase inhibitor superfamily protein* | 104.83 | -1.75 | **-3.36** |
| **AT2G30220** | *GDSL-motif esterase/acyltransferase/lipase* | 32.52 | -1.75 | **-3.36** |
| **AT2G13810** | *ALD1* | 96.10 | -1.75 | **-3.36** |
| **AT5G53380** | *WSD1-like* | 118.15 | -1.69 | **-3.23** |
| **AT1G78970** | *LUP1* | 171.80 | -1.69 | **-3.23** |
| **AT4G12470** | *AZI1* | 413.71 | -1.68 | **-3.19** |
| **AT5G01900** | *WRKY62* | 29.71 | -1.67 | **-3.18** |
| **AT1G50060** | *CAP superfamily protein* | 763.74 | -1.66 | **-3.16** |
| **AT3G02620** | *AAD4* | 542.60 | -1.66 | **-3.16** |
| **AT1G66800** | *unknown* | 2983.14 | -1.64 | **-3.12** |
| **AT3G56080** | *S-adenosyl-L-methionine-dependent methyltransferases superfamily protein* | 793.88 | -1.60 | **-3.03** |
| **AT2G41850** | *ADPG2* | 133.72 | -1.59 | **-3.01** |
| **AT5G14650** | *Pectin lyase-like* | 646.58 | -1.58 | **-3.00** |
| **AT3G45860** | *CRK4* | 44.33 | -1.57 | **-2.97** |
| **AT2G39310** | *JAL22* | 21024.19 | -1.56 | **-2.95** |
| **AT1G35625** | *RMR6* | 61.81 | -1.55 | **-2.93** |
| **AT1G36675** | *glycine-rich protein* | 27.45 | -1.55 | **-2.92** |
| **AT5G45950** | *GDSL-motif esterase/acyltransferase/lipase* | 29.57 | -1.54 | **-2.91** |
| **AT2G37870** | *T8P21.22* | 302.78 | -1.54 | **-2.91** |
| **AT3G10870** | *MES17* | 122.82 | -1.54 | **-2.91** |
| **AT1G09350** | *GOLS3* | 107.95 | -1.54 | **-2.90** |
| **AT5G57785** | *unknown* | 1061.18 | -1.53 | **-2.89** |
| **AT3G22231** | *PCC1* | 44.96 | -1.52 | **-2.87** |
| **AT5G02170** | *Transmembrane amino acid transporter* | 671.33 | -1.52 | **-2.86** |
| **AT5G10570** | *BHLH61* | 40.51 | -1.52 | **-2.86** |
| **AT1G12740** | *CYP87A2* | 2058.85 | -1.51 | **-2.84** |
| **AT2G16005** | *ROSY1* | 3456.12 | -1.49 | **-2.80** |
| **AT1G52690** | *LEA7* | 66.68 | -1.48 | **-2.78** |
| **AT3G61890** | *ATHB-12* | 423.07 | -1.47 | **-2.78** |
| **AT2G35300** | *LEA18* | 26.59 | -1.47 | **-2.77** |
| **AT4G04710** | *CPK22* | 77.02 | -1.47 | **-2.76** |
| **AT3G50820** | *PSBO2* | 863.43 | -1.47 | **-2.76** |
| **AT4G15480** | *UGT84A1* | 824.21 | -1.46 | **-2.76** |
| **AT5G20045** | *unknown membrane protein* | 92.86 | -1.46 | **-2.76** |
| **AT1G49975** | *unknown* | 37.59 | -1.45 | **-2.74** |
| **AT4G33120** | *S-adenosyl-L-methionine-dependent methyltransferases superfamily protein* | 1892.13 | -1.45 | **-2.73** |
| **AT1G52060** | *JAL9* | 1903.93 | -1.45 | **-2.73** |
| **AT1G06330** | *Heavy metal transport/detoxification superfamily protein* | 32.61 | -1.43 | **-2.70** |
| **AT1G75460** | *ATP-dependent protease* | 35.27 | -1.43 | **-2.70** |
| **AT5G56860** | *GATA21* | 47.48 | -1.43 | **-2.69** |
| **AT5G17220** | *GSTF12* | 50.47 | -1.41 | **-2.67** |
| **AT5G50260** | *CEP1* | 208.51 | -1.41 | **-2.66** |
| **AT5G15970** | *KIN2* | 1731.19 | -1.40 | **-2.65** |
| **AT5G15960** | *KIN1* | 325.34 | -1.40 | **-2.64** |
| **AT1G18265** | *DUF593* | 26.18 | -1.40 | **-2.64** |
| **AT2G26370** | *MD-2-related lipid recognition domain-containing protein;* | 317.65 | -1.40 | **-2.63** |
| **AT4G22230** | *defensin-like* | 771.63 | -1.39 | **-2.62** |
| **AT4G02770** | *psaD1* | 440.30 | -1.39 | **-2.62** |
| **AT5G23840** | *MD-2-related lipid recognition domain-containing protein* | 2605.84 | -1.38 | **-2.61** |
| **AT1G52070** | *JAL10* | 5503.69 | -1.38 | **-2.60** |
| **AT3G20160** | *PPPS2* | 225.06 | -1.37 | **-2.59** |
| **AT2G32540** | *CSLB4* | 25.44 | -1.36 | **-2.58** |
| **AT4G05100** | *MYB74* | 905.39 | -1.36 | **-2.57** |
| **AT5G42580** | *CYP705A12* | 1595.11 | -1.36 | **-2.57** |
| **AT5G54270** | *LHCB3* | 482.96 | -1.35 | **-2.56** |
| **AT1G10370** | *GSTU17* | 301.83 | -1.34 | **-2.53** |
| **AT4G22610** | *Bifunctional inhibitor/lipid-transfer protein* | 1227.51 | -1.33 | **-2.52** |
| **AT1G24130** | *Transducin/WD40 repeat-like superfamily protein* | 261.80 | -1.33 | **-2.52** |

**Table S3.** List of up-regulated fungal genes in *A. thaliana* (ATH) and *V. dahliae* (VDA) co-culture compared to *V. dahliae* grown separately. Cut-off was made at a log_2_-fold change of 1.33.

| **GeneID** | **Symbol/ info** | **base Mean** | **log2 Fold Change** | **Fold Change** |
| --- | --- | --- | --- | --- |
| **VDAG_03942** | *beta-lactamase family protein* | 21117.45 | 12.47 | **5677.01** |
| **VDAG_09583** | *alcohol oxidase* | 10188.27 | 12.22 | **4776.55** |
| **VDAG_05799** | *pectinesterase* | 3586.52 | 11.93 | **3911.46** |
| **VDAG_07647** | *hypothetical protein* | 1998.47 | 11.37 | **2654.66** |
| **VDAG_03386** | *hypothetical protein* | 1461.42 | 11.37 | **2639.61** |
| **VDAG_05268** | *hypothetical protein* | 905.88 | 11.10 | **2194.22** |
| **VDAG_03943** | *cyclopentanone 1,2-monooxygenase* | 46559.17 | 10.56 | **1508.17** |
| **VDAG_05011** | *hypothetical protein* | 22820.13 | 10.50 | **1446.42** |
| **VDAG_09536** | *pectate lyase* | 1074.24 | 10.35 | **1302.12** |
| **VDAG_10464** | *hypothetical protein* | 3999.65 | 10.09 | **1091.04** |
| **VDAG_09647** | *MFS transporter* | 775.84 | 9.99 | **1018.10** |
| **VDAG_09651** | *aldehyde reductase* | 605.11 | 9.83 | **908.79** |
| **VDAG_10527** | *lectin-B* | 94.40 | 9.74 | **855.82** |
| **VDAG_01172** | *hypothetical protein* | 1830.08 | 9.65 | **803.80** |
| **VDAG_06237** | *hypothetical protein* | 655.48 | 9.44 | **694.92** |
| **VDAG_02269** | *pantothenate transporter liz1* | 4328.94 | 9.24 | **604.35** |
| **VDAG_02908** | *secretory phospholipase A2* | 679.84 | 9.13 | **561.82** |
| **VDAG_05402** | *pectate lyase* | 899.79 | 9.03 | **521.01** |
| **VDAG_09253** | *sulfate transporter 4.1* | 2065.19 | 8.97 | **502.46** |
| **VDAG_05798** | *rhamnogalacturonan acetylesterase* | 1007.68 | 8.92 | **483.51** |
| **VDAG_06109** | *hypothetical protein* | 167.94 | 8.88 | **470.73** |
| **VDAG_07135** | *starch binding domain-containing protein* | 157.67 | 8.72 | **420.39** |
| **VDAG_07273** | *leucyl aminopeptidase* | 162.38 | 8.60 | **387.01** |
| **VDAG_05266** | *hypothetical protein* | 1086.78 | 8.57 | **378.98** |
| **VDAG_03387** | *hypothetical protein* | 713.89 | 8.56 | **377.45** |
| **VDAG_05115** | *zinc carboxypeptidase A* | 435.64 | 8.30 | **315.30** |
| **VDAG_07322** | *hypothetical protein* | 1287.40 | 8.30 | **314.57** |
| **VDAG_07191** | *high-affinity nicotinic acid transporter* | 659.78 | 8.28 | **311.43** |
| **VDAG_07323** | *hypothetical protein* | 1103.85 | 8.25 | **303.50** |
| **VDAG_07326** | *hypothetical protein* | 219.34 | 8.07 | **269.34** |
| **VDAG_08286** | *alpha-glucosides permease MPH2/3* | 690.50 | 8.07 | **269.31** |
| **VDAG_05269** | *casein kinase I isoform alpha* | 103.43 | 8.05 | **264.85** |
| **VDAG_09654** | *hypothetical protein* | 293.59 | 7.93 | **244.43** |
| **VDAG_07181** | *short chain dehydrogenase* | 203.40 | 7.91 | **240.91** |
| **VDAG_06947** | *hypothetical protein* | 262.63 | 7.86 | **231.52** |
| **VDAG_01193** | *high-affinity nicotinic acid transporter* | 2430.62 | 7.85 | **229.92** |
| **VDAG_09254** | *glutathione-independent formaldehyde dehydrogenase* | 513.16 | 7.81 | **223.78** |
| **VDAG_02979** | *high-affinity glucose transporter RGT2* | 385.97 | 7.65 | **200.73** |
| **VDAG_08101** | *aldehyde dehydrogenase* | 845.02 | 7.64 | **199.23** |
| **VDAG_06236** | *hypothetical protein* | 610.27 | 7.60 | **193.72** |
| **VDAG_07759** | *pectate lyase B* | 1144.65 | 7.56 | **189.04** |
| **VDAG_09073** | *hypothetical protein* | 1858.43 | 7.55 | **187.71** |
| **VDAG_02879** | *exopolygalacturonase* | 874.31 | 7.55 | **187.57** |
| **VDAG_08098** | *polygalacturonase* | 4325.65 | 7.55 | **186.82** |
| **VDAG_03784** | *hypothetical protein* | 283.22 | 7.47 | **177.77** |
| **VDAG_09753** | *hypothetical protein* | 42.86 | 7.41 | **170.32** |
| **VDAG_09648** | *dihydroxyacetone kinase* | 184.96 | 7.36 | **163.90** |
| **VDAG_07223** | *quinone oxidoreductase* | 806.24 | 7.33 | **160.35** |
| **VDAG_07267** | *pectate lyase C* | 101.95 | 7.22 | **148.76** |
| **VDAG_06204** | *ligninase H8* | 1399.50 | 7.17 | **143.97** |
| **VDAG_03418** | *extracellular elastinolytic metalloproteinase* | 151.94 | 7.15 | **141.73** |
| **VDAG_05819** | *WW domain-containing oxidoreductase* | 168.94 | 7.14 | **140.93** |
| **VDAG_07946** | *hypothetical protein* | 3679.67 | 7.09 | **135.89** |
| **VDAG_06948** | *beta-lactamase family protein* | 5027.50 | 7.08 | **134.87** |
| **VDAG_07325** | *hypothetical protein* | 349.48 | 7.06 | **133.77** |
| **VDAG_04827** | *acetylesterase* | 1948.60 | 7.05 | **132.64** |
| **VDAG_07769** | *phytanoyl-CoA dioxygenase family protein* | 599.77 | 6.99 | **127.32** |
| **VDAG_08105** | *cell wall glycosyl hydrolase YteR* | 318.12 | 6.98 | **126.07** |
| **VDAG_08281** | *integral membrane protein* | 47.86 | 6.98 | **125.93** |
| **VDAG_09534** | *aflatoxin biosynthesis polyketide synthase* | 1108.11 | 6.96 | **124.46** |
| **VDAG_03118** | *NmrA family protein* | 3448.99 | 6.85 | **115.62** |
| **VDAG_06284** | *carboxylesterase* | 65.83 | 6.76 | **108.20** |
| **VDAG_05374** | *MFS hexose transporter* | 452.15 | 6.75 | **107.70** |
| **VDAG_05050** | *choline monooxygenase* | 26.00 | 6.74 | **106.80** |
| **VDAG_07312** | *stress responsive A/B barrel domain-containing protein* | 127.45 | 6.73 | **106.39** |
| **VDAG_03389** | *hypothetical protein* | 262.06 | 6.67 | **102.17** |
| **VDAG_10460** | *amino-acid permease inda1* | 1190.41 | 6.67 | **102.06** |
| **VDAG_01701** | *hypothetical protein* | 51.15 | 6.64 | **100.06** |
| **VDAG_10160** | *hypothetical protein* | 13.99 | 6.61 | **97.53** |
| **VDAG_03398** | *aldo-keto reductase yakc* | 341.75 | 6.55 | **93.90** |
| **VDAG_00751** | *metalloprotease* | 145.25 | 6.53 | **92.26** |
| **VDAG_07566** | *pectate lyase* | 163.13 | 6.50 | **90.72** |
| **VDAG_06279** | *beta-glucosidase* | 690.55 | 6.50 | **90.29** |
| **VDAG_06994** | *alpha-glucosides permease MPH2/3* | 133.48 | 6.45 | **87.14** |
| **VDAG_01687** | *rhamnogalacturonan acetylesterase* | 402.77 | 6.44 | **86.78** |
| **VDAG_05175** | *tubulin beta-3 chain* | 1122.15 | 6.39 | **83.92** |
| **VDAG_07645** | *hypothetical protein* | 62.29 | 6.38 | **83.14** |
| **VDAG_07646** | *acetamidase* | 220.54 | 6.35 | **81.44** |
| **VDAG_03392** | *hypothetical protein* | 282.46 | 6.29 | **78.39** |
| **VDAG_05651** | *hypothetical protein* | 238.82 | 6.28 | **77.83** |
| **VDAG_05278** | *hypothetical protein* | 130.93 | 6.25 | **76.16** |
| **VDAG_10515** | *hypothetical protein* | 5823.25 | 6.23 | **75.29** |
| **VDAG_09882** | *beta-Ig-H3/Fasciclin* | 143.00 | 6.22 | **74.70** |
| **VDAG_09337** | *hypothetical protein* | 264.21 | 6.20 | **73.65** |
| **VDAG_04103** | *hypothetical protein* | 848.58 | 6.16 | **71.50** |
| **VDAG_03941** | *regulatory protein alcR* | 172.67 | 6.12 | **69.69** |
| **VDAG_06367** | *hypothetical protein* | 443.30 | 5.95 | **62.03** |
| **VDAG_07321** | *pisatin demethylase* | 218.68 | 5.94 | **61.59** |
| **VDAG_09526** | *FAD binding domain-containing protein* | 3841.73 | 5.94 | **61.55** |
| **VDAG_05650** | *trypsin* | 28.79 | 5.87 | **58.35** |
| **VDAG_04017** | *endoglucanase-1* | 116.53 | 5.82 | **56.40** |
| **VDAG_03603** | *TPR domain-containing protein* | 197.99 | 5.77 | **54.49** |
| **VDAG_07075** | *7-dehydrocholesterol reductase* | 48.38 | 5.76 | **54.29** |
| **VDAG_07384** | *hypothetical protein* | 299.44 | 5.75 | **53.90** |
| **VDAG_01578** | *hypothetical protein* | 81.34 | 5.74 | **53.56** |
| **VDAG_07494** | *secreted protein* | 51.19 | 5.73 | **53.06** |
| **VDAG_10472** | *CFEM domain-containing protein* | 16.38 | 5.72 | **52.88** |
| **VDAG_07608** | *exopolygalacturonase* | 32.59 | 5.72 | **52.70** |
| **VDAG_02475** | *reductase* | 905.03 | 5.72 | **52.63** |
| **VDAG_05466** | *esterase* | 13.08 | 5.69 | **51.50** |
| **VDAG_07265** | *hypothetical protein* | 247.94 | 5.68 | **51.26** |
| **VDAG_06738** | *glycosyl hydrolase* | 108.18 | 5.67 | **51.00** |
| **VDAG_05101** | *hypothetical protein* | 132.41 | 5.67 | **50.99** |
| **VDAG_02898** | *exoglucanase* | 72.73 | 5.66 | **50.61** |
| **VDAG_02978** | *sucrose-6-phosphate hydrolase* | 167.63 | 5.63 | **49.51** |
| **VDAG_07328** | *hypothetical protein* | 185.30 | 5.63 | **49.38** |
| **VDAG_03479** | *quinate permease* | 1320.85 | 5.62 | **49.33** |
| **VDAG_01781** | *polygalacturonase* | 592.65 | 5.62 | **49.33** |
| **VDAG_05505** | *hypothetical protein* | 17.68 | 5.62 | **49.29** |
| **VDAG_07727** | *polysaccharide deacetylase family protein* | 694.32 | 5.49 | **44.95** |
| **VDAG_03496** | *integral membrane protein* | 312.16 | 5.49 | **44.87** |
| **VDAG_08099** | *NADPH-dependent methylglyoxal reductase GRE2* | 312.72 | 5.48 | **44.77** |
| **VDAG_03639** | *choline dehydrogenase* | 361.45 | 5.44 | **43.56** |
| **VDAG_04531** | *retinol dehydrogenase* | 134.31 | 5.44 | **43.42** |
| **VDAG_04019** | *secretory phospholipase A2* | 94.19 | 5.40 | **42.23** |
| **VDAG_09269** | *NAD(P) transhydrogenase* | 2395.97 | 5.38 | **41.75** |
| **VDAG_03762** | *fungal cellulose binding domain-containing protein* | 128.11 | 5.38 | **41.70** |
| **VDAG_08023** | *hypothetical protein* | 29.56 | 5.36 | **40.96** |
| **VDAG_09535** | *vacuolar basic amino acid transporter 2* | 55.90 | 5.26 | **38.23** |
| **VDAG_09088** | *MFS transporter* | 131.43 | 5.23 | **37.64** |
| **VDAG_08275** | *dienelactone hydrolase family protein* | 1032.87 | 5.19 | **36.46** |
| **VDAG_09712** | *succinate/fumarate mitochondrial transporter* | 963.07 | 5.19 | **36.45** |
| **VDAG_07664** | *hypothetical protein* | 480.42 | 5.18 | **36.25** |
| **VDAG_04611** | *G-protein coupled receptor* | 148.11 | 5.17 | **36.03** |
| **VDAG_01738** | *hypothetical protein* | 28.23 | 5.17 | **35.96** |
| **VDAG_04784** | *alcohol oxidase* | 689.95 | 5.16 | **35.72** |
| **VDAG_07240** | *carboxypeptidase A4* | 198.40 | 5.14 | **35.17** |
| **VDAG_09736** | *calpain clp-1* | 754.80 | 5.11 | **34.65** |
| **VDAG_03371** | *carboxylic acid transport protein* | 2426.65 | 5.09 | **34.01** |
| **VDAG_05267** | *hypothetical protein* | 507.65 | 5.08 | **33.85** |
| **VDAG_07673** | *gst19* | 1797.24 | 5.08 | **33.83** |
| **VDAG_03649** | *sugar transporter* | 117.59 | 5.05 | **33.16** |
| **VDAG_05751** | *acetylcholinesterase* | 64.38 | 5.05 | **33.04** |
| **VDAG_05094** | *FAD binding domain-containing protein* | 539.07 | 5.04 | **32.99** |
| **VDAG_03626** | *allantoate permease* | 90.21 | 5.03 | **32.78** |
| **VDAG_07195** | *hypothetical protein* | 466.29 | 5.03 | **32.67** |
| **VDAG_02089** | *quinate permease* | 73.09 | 5.03 | **32.64** |
| **VDAG_02079** | *metalloprotease* | 496.83 | 5.02 | **32.51** |
| **VDAG_00752** | *exoglucanase* | 214.60 | 5.01 | **32.26** |
| **VDAG_09621** | *hypothetical protein* | 68.99 | 4.98 | **31.62** |
| **VDAG_04705** | *hypothetical protein* | 19.14 | 4.97 | **31.43** |
| **VDAG_09774** | *hypothetical protein* | 236.03 | 4.97 | **31.37** |
| **VDAG_04762** | *beta-1,6-galactanase* | 270.95 | 4.97 | **31.28** |
| **VDAG_09383** | *hypothetical protein* | 15.35 | 4.95 | **30.84** |
| **VDAG_02705** | *hypothetical protein* | 641.60 | 4.94 | **30.76** |
| **VDAG_00474** | *hypothetical protein* | 23.08 | 4.93 | **30.51** |
| **VDAG_03551** | *pectate lyase* | 93.18 | 4.90 | **29.89** |
| **VDAG_00783** | *hypothetical protein* | 98.76 | 4.88 | **29.43** |
| **VDAG_03965** | *hypothetical protein* | 1558.50 | 4.88 | **29.41** |
| **VDAG_04685** | *AdhA* | 26.13 | 4.87 | **29.25** |
| **VDAG_01179** | *aldehyde dehydrogenase* | 16.61 | 4.87 | **29.24** |
| **VDAG_07077** | *hypothetical protein* | 92.32 | 4.86 | **29.14** |
| **VDAG_07327** | *aldehyde dehydrogenase* | 226.12 | 4.86 | **29.05** |
| **VDAG_00499** | *endothiapepsin* | 46.20 | 4.85 | **28.77** |
| **VDAG_09336** | *hypothetical protein* | 205.55 | 4.83 | **28.39** |
| **VDAG_04466** | *pfs domain-containing protein* | 232.14 | 4.81 | **27.97** |
| **VDAG_03844** | *hypothetical protein* | 2557.22 | 4.76 | **27.06** |
| **VDAG_02078** | *hypothetical protein* | 960.67 | 4.75 | **26.98** |
| **VDAG_04921** | *hypothetical protein* | 128.83 | 4.75 | **26.87** |
| **VDAG_06953** | *kinesin light chain* | 222.42 | 4.74 | **26.79** |
| **VDAG_01556** | *neutral ceramidase* | 339.16 | 4.73 | **26.54** |
| **VDAG_02013** | *aldehyde dehydrogenase* | 7336.18 | 4.73 | **26.49** |
| **VDAG_08118** | *high-affinity nicotinic acid transporter* | 57.30 | 4.72 | **26.26** |
| **VDAG_09527** | *hydrolase* | 4688.32 | 4.71 | **26.14** |
| **VDAG_07119** | *rhamnogalacturonate lyase* | 263.92 | 4.70 | **25.94** |
| **VDAG_04513** | *hexose transporter protein* | 246.68 | 4.70 | **25.93** |
| **VDAG_07675** | *hypothetical protein* | 80.87 | 4.69 | **25.83** |
| **VDAG_08142** | *hypothetical protein* | 268.54 | 4.69 | **25.81** |
| **VDAG_07185** | *glucan 1,3-beta-glucosidase* | 4884.23 | 4.68 | **25.65** |
| **VDAG_09653** | *finger protein* | 192.05 | 4.68 | **25.64** |
| **VDAG_07242** | *pectate lyase* | 56.46 | 4.67 | **25.54** |
| **VDAG_07305** | *glycoside hydrolase* | 38.59 | 4.67 | **25.41** |
| **VDAG_09528** | *hypothetical protein* | 1947.23 | 4.65 | **25.05** |
| **VDAG_03588** | *carboxypeptidase B* | 1121.57 | 4.62 | **24.64** |
| **VDAG_05248** | *hypothetical protein* | 137.38 | 4.61 | **24.50** |
| **VDAG_04341** | *hypothetical protein* | 655.30 | 4.61 | **24.43** |
| **VDAG_09063** | *rhamnogalacturonase B* | 900.55 | 4.60 | **24.27** |
| **VDAG_09381** | *hypothetical protein* | 1080.91 | 4.59 | **24.15** |
| **VDAG_09529** | *hypothetical protein* | 1443.26 | 4.59 | **24.04** |
| **VDAG_03755** | *hypothetical protein* | 640.80 | 4.59 | **24.03** |
| **VDAG_05550** | *general alpha-glucoside permease* | 920.77 | 4.59 | **24.02** |
| **VDAG_03390** | *4-coumarate-CoA ligase* | 28.85 | 4.58 | **23.98** |
| **VDAG_02888** | *hypothetical protein* | 37.04 | 4.58 | **23.93** |
| **VDAG_08144** | *homogentisate 1,2-dioxygenase* | 13.15 | 4.58 | **23.87** |
| **VDAG_03495** | *FAD binding domain-containing protein* | 365.41 | 4.57 | **23.81** |
| **VDAG_04540** | *metallo-beta-lactamase family protein* | 285.17 | 4.57 | **23.77** |
| **VDAG_09925** | *OefC* | 376.05 | 4.57 | **23.71** |
| **VDAG_05483** | *hypothetical protein* | 319.44 | 4.56 | **23.66** |
| **VDAG_05036** | *cell wall glycosyl hydrolase YteR* | 294.15 | 4.56 | **23.66** |
| **VDAG_01264** | *mono* | 455.18 | 4.56 | **23.59** |
| **VDAG_00329** | *hypothetical protein* | 27.19 | 4.52 | **22.97** |
| **VDAG_01180** | *ankyrin repeat protein* | 130.47 | 4.50 | **22.70** |
| **VDAG_02904** | *pectate lyase B* | 58.61 | 4.47 | **22.21** |
| **VDAG_06238** | *lactose permease* | 321.58 | 4.47 | **22.20** |
| **VDAG_10174** | *hypothetical protein* | 66.32 | 4.46 | **22.07** |
| **VDAG_10476** | *sugar transporter* | 23.89 | 4.45 | **21.92** |
| **VDAG_04807** | *hypothetical protein* | 12.95 | 4.45 | **21.83** |
| **VDAG_10456** | *hypothetical protein* | 250.53 | 4.44 | **21.69** |
| **VDAG_07937** | *hypothetical protein* | 37.39 | 4.44 | **21.66** |
| **VDAG_08891** | *hypothetical protein* | 68.50 | 4.43 | **21.63** |
| **VDAG_05249** | *hypothetical protein* | 24.69 | 4.43 | **21.56** |
| **VDAG_09739** | *galactan 1,3-beta-galactosidase* | 99.94 | 4.43 | **21.54** |
| **VDAG_07166** | *carnitine O-palmitoyltransferase I* | 1518.26 | 4.43 | **21.50** |
| **VDAG_05048** | *quinate permease* | 150.67 | 4.42 | **21.44** |
| **VDAG_09121** | *maltose permease MAL31* | 157.57 | 4.42 | **21.39** |
| **VDAG_03499** | *hypothetical protein* | 12.70 | 4.41 | **21.27** |
| **VDAG_02084** | *hypothetical protein* | 67.39 | 4.41 | **21.21** |
| **VDAG_00764** | *sugar transporter STL1* | 33.10 | 4.40 | **21.18** |
| **VDAG_01165** | *NADP-dependent mannitol dehydrogenase* | 212.20 | 4.40 | **21.13** |
| **VDAG_08147** | *maleylacetate reductase* | 33.35 | 4.39 | **20.99** |
| **VDAG_03419** | *hypothetical protein* | 551.98 | 4.39 | **20.91** |
| **VDAG_05102** | *hypothetical protein* | 95.02 | 4.36 | **20.60** |
| **VDAG_05448** | *metalloprotease* | 684.33 | 4.36 | **20.51** |
| **VDAG_02279** | *hypothetical protein* | 737.51 | 4.34 | **20.31** |
| **VDAG_09531** | *hypothetical protein* | 205.85 | 4.34 | **20.23** |
| **VDAG_02085** | *mannan endo-1,4-beta-mannosidase* | 84.58 | 4.34 | **20.22** |
| **VDAG_06023** | *isotrichodermin C-15 hydroxylase* | 88.03 | 4.33 | **20.12** |
| **VDAG_02909** | *hypothetical protein* | 73.25 | 4.32 | **19.92** |
| **VDAG_04962** | *FAD binding domain-containing protein* | 24.29 | 4.31 | **19.81** |
| **VDAG_02827** | *hypothetical protein* | 106.00 | 4.29 | **19.57** |
| **VDAG_02273** | *hypothetical protein* | 1396.33 | 4.29 | **19.57** |
| **VDAG_02887** | *general alpha-glucoside permease* | 37.50 | 4.27 | **19.32** |
| **VDAG_08654** | *acetyl-coenzyme A synthetase* | 1291.60 | 4.27 | **19.26** |
| **VDAG_06280** | *hypothetical protein* | 60.39 | 4.26 | **19.18** |
| **VDAG_04961** | *aldehyde dehydrogenase* | 57.09 | 4.25 | **19.03** |
| **VDAG_00808** | *acetylxylan esterase* | 135.94 | 4.25 | **18.97** |
| **VDAG_07253** | *hypothetical protein* | 117.80 | 4.24 | **18.94** |
| **VDAG_03938** | *cytochrome P450 52E2* | 671.51 | 4.24 | **18.92** |
| **VDAG_03714** | *sugar transporter* | 48.52 | 4.22 | **18.69** |
| **VDAG_08157** | *hypothetical protein* | 147.06 | 4.21 | **18.47** |
| **VDAG_08097** | *polygalacturonase* | 3076.69 | 4.20 | **18.44** |
| **VDAG_09366** | *polygalacturonase* | 2953.73 | 4.17 | **17.95** |
| **VDAG_10203** | *hypothetical protein* | 37.73 | 4.16 | **17.92** |
| **VDAG_06283** | *pantothenate transporter liz1* | 91.98 | 4.15 | **17.76** |
| **VDAG_07290** | *carboxypeptidase B* | 375.73 | 4.15 | **17.76** |
| **VDAG_07320** | *acyl-CoA dehydrogenase* | 358.91 | 4.13 | **17.50** |
| **VDAG_06147** | *hypothetical protein* | 7.27 | 4.13 | **17.50** |
| **VDAG_08119** | *hypothetical protein* | 21.53 | 4.12 | **17.34** |
| **VDAG_07188** | *allantoate permease* | 99.68 | 4.11 | **17.28** |
| **VDAG_07316** | *homogentisate 1,2-dioxygenase* | 101.44 | 4.11 | **17.27** |
| **VDAG_03636** | *N-acetyltransferase ats1* | 16.12 | 4.11 | **17.27** |
| **VDAG_04960** | *salicylate hydroxylase* | 142.33 | 4.09 | **17.07** |
| **VDAG_00621** | *hypothetical protein* | 272.29 | 4.09 | **17.00** |
| **VDAG_03086** | *hypothetical protein* | 225.39 | 4.08 | **16.97** |
| **VDAG_00066** | *hypothetical protein* | 266.61 | 4.08 | **16.96** |
| **VDAG_00389** | *E3 ubiquitin-protein ligase CCNB1IP1* | 20.68 | 4.08 | **16.93** |
| **VDAG_04607** | *malate dehydrogenase* | 319.23 | 4.08 | **16.92** |
| **VDAG_08456** | *hypothetical protein* | 53.32 | 4.08 | **16.87** |
| **VDAG_04352** | *hypothetical protein* | 1470.57 | 4.07 | **16.84** |
| **VDAG_08230** | *6-hydroxy-D-nicotine oxidase* | 7.55 | 4.07 | **16.83** |
| **VDAG_09848** | *hypothetical protein* | 54.10 | 4.07 | **16.82** |
| **VDAG_04808** | *hypothetical protein* | 23.39 | 4.06 | **16.71** |
| **VDAG_06250** | *hypothetical protein* | 52.73 | 4.06 | **16.71** |
| **VDAG_03656** | *pectin lyase B* | 30.68 | 4.04 | **16.49** |
| **VDAG_09290** | *cytochrome P450 71B28* | 101.93 | 4.04 | **16.41** |
| **VDAG_09532** | *serine 3-dehydrogenase* | 1396.47 | 4.03 | **16.38** |
| **VDAG_08067** | *pectate lyase B* | 151.83 | 4.02 | **16.25** |
| **VDAG_04334** | *alpha-glucosides permease MPH2/3* | 23.82 | 4.01 | **16.14** |
| **VDAG_01743** | *hypothetical protein* | 334.06 | 4.00 | **16.00** |
| **VDAG_09807** | *aromatic ring-opening dioxygenase family protein* | 13.65 | 4.00 | **16.00** |
| **VDAG_05976** | *alpha-amylase A type-1/2* | 798.60 | 4.00 | **15.99** |
| **VDAG_09058** | *hypothetical protein* | 155.26 | 3.98 | **15.74** |
| **VDAG_07681** | *ATP-binding cassette sub-family G member 5* | 276.44 | 3.97 | **15.70** |
| **VDAG_04875** | *hypothetical protein* | 174.26 | 3.97 | **15.62** |
| **VDAG_04323** | *oligo-1,6-glucosidase* | 145.37 | 3.96 | **15.54** |
| **VDAG_03441** | *hypothetical protein* | 280.42 | 3.95 | **15.48** |
| **VDAG_08145** | *C2H2 type zinc finger domain-containing protein* | 274.52 | 3.95 | **15.46** |
| **VDAG_06022** | *hypothetical protein* | 14.89 | 3.95 | **15.45** |
| **VDAG_04527** | *transaldolase* | 45.11 | 3.95 | **15.44** |
| **VDAG_09988** | *phosphatidylethanolamine-binding protein* | 441.65 | 3.95 | **15.43** |
| **VDAG_03108** | *hypothetical protein* | 3047.05 | 3.95 | **15.41** |
| **VDAG_03808** | *endo-1,4-beta-xylanase* | 48.33 | 3.94 | **15.38** |
| **VDAG_04994** | *cytochrome P450 1A2* | 54.80 | 3.94 | **15.36** |
| **VDAG_08120** | *hypothetical protein* | 58.46 | 3.93 | **15.26** |
| **VDAG_05250** | *hypothetical protein* | 10.91 | 3.92 | **15.12** |
| **VDAG_00184** | *amino acid adenylation* | 160.03 | 3.92 | **15.10** |
| **VDAG_09075** | *hypothetical protein* | 17.11 | 3.92 | **15.10** |
| **VDAG_09380** | *hypothetical protein* | 29.28 | 3.91 | **15.07** |
| **VDAG_06939** | *proton myo-inositol cotransporter* | 290.85 | 3.91 | **15.07** |
| **VDAG_09584** | *cellulose-binding protein* | 49.95 | 3.91 | **14.99** |
| **VDAG_09813** | *C6 transcription factor RegA* | 117.64 | 3.90 | **14.92** |
| **VDAG_07133** | *hypothetical protein* | 10.67 | 3.90 | **14.89** |
| **VDAG_08231** | *C6 zinc finger protein* | 51.41 | 3.90 | **14.88** |
| **VDAG_07950** | *hypothetical protein* | 4.07 | 3.88 | **14.76** |
| **VDAG_00147** | *hypothetical protein* | 123.30 | 3.86 | **14.57** |
| **VDAG_01596** | *D-3-phosphoglycerate dehydrogenase* | 494.27 | 3.86 | **14.55** |
| **VDAG_05649** | *BNR/Asp-box repeat domain-containing protein* | 71.03 | 3.86 | **14.51** |
| **VDAG_05235** | *hypothetical protein* | 4.05 | 3.86 | **14.48** |
| **VDAG_08482** | *hypothetical protein* | 273.93 | 3.85 | **14.40** |
| **VDAG_05378** | *alpha-glucosidase* | 132.39 | 3.84 | **14.28** |
| **VDAG_05103** | *isochorismatase hydrolase* | 8631.57 | 3.83 | **14.26** |
| **VDAG_04977** | *endopolygalacturonase* | 5327.07 | 3.83 | **14.22** |
| **VDAG_01103** | *hypothetical protein* | 296.67 | 3.82 | **14.16** |
| **VDAG_10050** | *dihydrodipicolinate synthase* | 1183.57 | 3.82 | **14.12** |
| **VDAG_06201** | *quinate permease* | 526.36 | 3.82 | **14.08** |
| **VDAG_02660** | *ferric reductase* | 403.75 | 3.82 | **14.08** |
| **VDAG_09530** | *hypothetical protein* | 58.18 | 3.81 | **13.98** |
| **VDAG_03121** | *allantoate permease* | 149.28 | 3.80 | **13.90** |
| **VDAG_08502** | *hypothetical protein* | 7.02 | 3.79 | **13.85** |
| **VDAG_07392** | *hypothetical protein* | 347.22 | 3.79 | **13.81** |
| **VDAG_04336** | *alpha-N-arabinofuranosidase* | 46.69 | 3.79 | **13.79** |
| **VDAG_00857** | *hypothetical protein* | 45.80 | 3.78 | **13.78** |
| **VDAG_03528** | *glucoamylase* | 199.30 | 3.77 | **13.69** |
| **VDAG_01704** | *fungal specific transcription factor domain-containing protein* | 94.91 | 3.77 | **13.63** |
| **VDAG_03713** | *hypothetical protein* | 158.19 | 3.77 | **13.62** |
| **VDAG_09615** | *hypothetical protein* | 143.16 | 3.76 | **13.50** |
| **VDAG_08685** | *pectate lyase* | 77.19 | 3.75 | **13.48** |
| **VDAG_03438** | *hypothetical protein* | 11.82 | 3.75 | **13.46** |
| **VDAG_02944** | *hypothetical protein* | 144.87 | 3.75 | **13.46** |
| **VDAG_04175** | *SAM and PH domain-containing protein* | 432.79 | 3.74 | **13.37** |
| **VDAG_06756** | *aldo-keto reductase yakc* | 313.35 | 3.73 | **13.30** |
| **VDAG_02102** | *hypothetical protein* | 26.10 | 3.73 | **13.24** |
| **VDAG_03897** | *beta-lactamase family protein* | 812.87 | 3.72 | **13.21** |
| **VDAG_07680** | *hypothetical protein* | 60.17 | 3.72 | **13.21** |
| **VDAG_04821** | *hypothetical protein* | 4.83 | 3.72 | **13.14** |
| **VDAG_10443** | *rhamnogalacturonan lyase* | 147.94 | 3.71 | **13.08** |
| **VDAG_09754** | *hypothetical protein* | 113.98 | 3.71 | **13.05** |
| **VDAG_05643** | *hypothetical protein* | 22.36 | 3.69 | **12.94** |
| **VDAG_05141** | *high-affinity potassium transport protein* | 125.81 | 3.69 | **12.91** |
| **VDAG_03740** | *hypothetical protein* | 398.47 | 3.69 | **12.90** |
| **VDAG_05303** | *hypothetical protein* | 457.78 | 3.69 | **12.87** |
| **VDAG_01288** | *hypothetical protein* | 727.45 | 3.69 | **12.87** |
| **VDAG_03507** | *aldo-keto reductase yakc* | 248.74 | 3.68 | **12.82** |
| **VDAG_05410** | *lipase* | 46.59 | 3.68 | **12.80** |
| **VDAG_05569** | *hypothetical protein* | 33.26 | 3.67 | **12.69** |
| **VDAG_03335** | *gibberellin 20 oxidase* | 156.11 | 3.66 | **12.61** |
| **VDAG_03403** | *cation diffusion facilitator 1* | 204.16 | 3.65 | **12.58** |
| **VDAG_06740** | *high-affinity methionine permease* | 1088.74 | 3.65 | **12.52** |
| **VDAG_02358** | *hypothetical protein* | 1.16 | 3.64 | 12.46 |
| **VDAG_00904** | *hypothetical protein* | 17.33 | 3.61 | **12.25** |
| **VDAG_04318** | *hypothetical protein* | 26.23 | 3.61 | **12.22** |
| **VDAG_09622** | *argininosuccinate lyase* | 60.66 | 3.61 | **12.20** |
| **VDAG_09734** | *major myo-inositol transporter iolT* | 2176.16 | 3.60 | **12.16** |
| **VDAG_08149** | *hypothetical protein* | 128.75 | 3.60 | **12.16** |
| **VDAG_07134** | *carboxypeptidase A5* | 4.50 | 3.60 | **12.10** |
| **VDAG_01698** | *FAD/FMN-containing dehydrogenase* | 205.99 | 3.60 | **12.08** |
| **VDAG_04530** | *UVI-1 protein* | 811.25 | 3.59 | **12.08** |
| **VDAG_07138** | *hypothetical protein* | 129.01 | 3.59 | **12.02** |
| **VDAG_10414** | *carboxypeptidase* | 736.02 | 3.59 | **12.01** |
| **VDAG_09847** | *maltose permease* | 27.06 | 3.58 | **12.00** |
| **VDAG_07046** | *hypothetical protein* | 11.82 | 3.58 | **12.00** |
| **VDAG_03758** | *hypothetical protein* | 34.39 | 3.58 | **11.99** |
| **VDAG_09317** | *hypothetical protein* | 249.09 | 3.58 | **11.98** |
| **VDAG_04310** | *hypothetical protein* | 228.01 | 3.58 | **11.95** |
| **VDAG_06133** | *amine oxidase* | 386.84 | 3.57 | **11.91** |
| **VDAG_08004** | *transcriptional activator xlnR* | 130.49 | 3.57 | **11.87** |
| **VDAG_02241** | *linoleate diol synthase* | 2703.52 | 3.57 | **11.86** |
| **VDAG_10450** | *galactosyl transferase GMA12/MNN10 family protein* | 38.61 | 3.57 | **11.86** |
| **VDAG_07349** | *cysteine-rich-protein* | 270.94 | 3.57 | **11.84** |
| **VDAG_07682** | *hypothetical protein* | 186.20 | 3.57 | **11.84** |
| **VDAG_08124** | *sugar transporter family protein* | 1402.88 | 3.56 | **11.82** |
| **VDAG_04820** | *maltose permease* | 71.02 | 3.56 | **11.81** |
| **VDAG_03420** | *hypothetical protein* | 3606.48 | 3.56 | **11.78** |
| **VDAG_06707** | *beta,beta-carotene 9',10'-dioxygenase* | 345.66 | 3.56 | **11.77** |
| **VDAG_00407** | *pisatin demethylase* | 134.25 | 3.56 | **11.76** |
| **VDAG_09991** | *quinate permease* | 4320.24 | 3.55 | **11.73** |
| **VDAG_08096** | *interferon-induced GTP-binding protein Mx1* | 66.08 | 3.55 | **11.69** |
| **VDAG_05467** | *monooxygenase* | 13.79 | 3.55 | **11.68** |
| **VDAG_09506** | *5'/3'-nucleotidase SurE family protein* | 182.85 | 3.54 | **11.60** |
| **VDAG_07141** | *H+/hexose cotransporter 1* | 204.81 | 3.52 | **11.50** |
| **VDAG_09811** | *hypothetical protein* | 34.64 | 3.52 | **11.47** |
| **VDAG_05297** | *3-alpha-(or 20-beta)-hydroxysteroid dehydrogenase* | 29.12 | 3.51 | **11.41** |
| **VDAG_04726** | *hypothetical protein* | 96.69 | 3.50 | **11.35** |
| **VDAG_00531** | *high-affinity nicotinic acid transporter* | 53.22 | 3.50 | **11.32** |
| **VDAG_03236** | *alpha-galactosidase* | 142.96 | 3.50 | **11.31** |
| **VDAG_04703** | *general alpha-glucoside permease* | 15.89 | 3.48 | **11.19** |
| **VDAG_09282** | *proton myo-inositol cotransporter* | 161.77 | 3.48 | **11.16** |
| **VDAG_09582** | *hypothetical protein* | 95.26 | 3.48 | **11.15** |
| **VDAG_02207** | *uracil permease* | 277.31 | 3.48 | **11.15** |
| **VDAG_03761** | *hypothetical protein* | 52.50 | 3.48 | **11.14** |
| **VDAG_05416** | *guanyl-specific ribonuclease F1* | 72.56 | 3.48 | **11.14** |
| **VDAG_09507** | *hypothetical protein* | 4.53 | 3.48 | **11.13** |
| **VDAG_05514** | *pantothenate transporter liz1* | 69.42 | 3.48 | **11.13** |
| **VDAG_04553** | *hypothetical protein* | 299.55 | 3.48 | **11.12** |
| **VDAG_10467** | *dicarboxylic amino acid permease* | 3763.24 | 3.47 | **11.11** |
| **VDAG_04830** | *hypothetical protein* | 47.33 | 3.46 | **11.02** |
| **VDAG_09071** | *high-affinity glucose transporter RGT2* | 18877.73 | 3.46 | **10.99** |
| **VDAG_05456** | *pisatin demethylase* | 184.12 | 3.46 | **10.99** |
| **VDAG_02115** | *hypothetical protein* | 32.17 | 3.44 | **10.85** |
| **VDAG_09794** | *hypothetical protein* | 385.77 | 3.43 | **10.81** |
| **VDAG_07651** | *glucose/galactose transporter* | 377.20 | 3.43 | **10.80** |
| **VDAG_07887** | *benzoate 4-monooxygenase* | 448.43 | 3.43 | **10.77** |
| **VDAG_01838** | *hypothetical protein* | 13.61 | 3.43 | **10.74** |
| **VDAG_02958** | *guanylate kinase* | 4.28 | 3.42 | **10.73** |
| **VDAG_03439** | *hypothetical protein* | 138.75 | 3.42 | **10.73** |
| **VDAG_00169** | *hypothetical protein* | 69.39 | 3.42 | **10.71** |
| **VDAG_09368** | *hypothetical protein* | 304.23 | 3.42 | **10.71** |
| **VDAG_05469** | *protein kinase domain-containing protein* | 39.91 | 3.42 | **10.67** |
| **VDAG_02270** | *hypothetical protein* | 14.76 | 3.41 | **10.64** |
| **VDAG_07120** | *integral membrane protein* | 921.29 | 3.41 | **10.64** |
| **VDAG_10204** | *vitamin H transporter* | 14.19 | 3.41 | **10.64** |
| **VDAG_05209** | *hypothetical protein* | 2.09 | 3.39 | **10.47** |
| **VDAG_05191** | *hypothetical protein* | 67.05 | 3.38 | **10.44** |
| **VDAG_03707** | *alpha-N-arabinofuranosidase* | 71.03 | 3.38 | **10.42** |
| **VDAG_01684** | *maltose permease MAL31* | 78.26 | 3.38 | **10.40** |
| **VDAG_09276** | *hypothetical protein* | 27.97 | 3.37 | **10.37** |
| **VDAG_09864** | *ankyrin-1* | 65.99 | 3.37 | **10.35** |
| **VDAG_01904** | *hypothetical protein* | 62.93 | 3.37 | **10.35** |
| **VDAG_07113** | *hypothetical protein* | 78.37 | 3.35 | **10.21** |
| **VDAG_04547** | *hypothetical protein* | 168.32 | 3.35 | **10.21** |
| **VDAG_06747** | *hypothetical protein* | 127.51 | 3.35 | **10.20** |
| **VDAG_04690** | *phenylacetone monooxygenase* | 25.89 | 3.35 | **10.16** |
| **VDAG_02764** | *urea active transporter* | 128.34 | 3.33 | **10.07** |
| **VDAG_06070** | *hypothetical protein* | 209.70 | 3.32 | **9.96** |
| **VDAG_06211** | *hypothetical protein* | 48.49 | 3.31 | **9.89** |
| **VDAG_09709** | *C2H2 type zinc finger domain-containing protein* | 163.15 | 3.30 | **9.84** |
| **VDAG_02751** | *hypothetical protein* | 26.71 | 3.30 | **9.83** |
| **VDAG_07259** | *quinate permease* | 19.80 | 3.30 | **9.83** |
| **VDAG_01003** | *hypothetical protein* | 28.90 | 3.29 | **9.81** |
| **VDAG_09750** | *beta-glucosidase* | 1032.78 | 3.29 | **9.75** |
| **VDAG_05564** | *cytochrome b2* | 24.32 | 3.28 | **9.74** |
| **VDAG_09652** | *galactonate dehydratase* | 263.72 | 3.28 | **9.73** |
| **VDAG_08381** | *galactose-proton symporter* | 157.48 | 3.27 | **9.64** |
| **VDAG_04795** | *cellulose-growth-specific protein* | 14.02 | 3.26 | **9.58** |
| **VDAG_01403** | *hypothetical protein* | 46.65 | 3.26 | **9.58** |
| **VDAG_04691** | *lipase* | 45.06 | 3.25 | **9.54** |
| **VDAG_08213** | *hypothetical protein* | 125.76 | 3.25 | **9.54** |
| **VDAG_02240** | *hypothetical protein* | 1121.77 | 3.25 | **9.50** |
| **VDAG_07160** | *hypothetical protein* | 599.89 | 3.24 | **9.46** |
| **VDAG_06955** | *hypothetical protein* | 295.82 | 3.24 | **9.42** |
| **VDAG_00390** | *hypothetical protein* | 107.51 | 3.23 | **9.40** |
| **VDAG_04974** | *glucose transporter* | 191.47 | 3.23 | **9.38** |
| **VDAG_09077** | *acetyltransferase* | 72.12 | 3.23 | **9.37** |
| **VDAG_05708** | *endoglucanase II* | 3.28 | 3.23 | **9.37** |
| **VDAG_09649** | *IBR finger domain-containing protein* | 1.19 | 3.23 | 9.37 |
| **VDAG_01700** | *glucarate dehydratase* | 27.68 | 3.22 | **9.29** |
| **VDAG_01866** | *xylosidase/arabinosidase* | 165.33 | 3.21 | **9.25** |
| **VDAG_02101** | *cytochrome P450 4d8* | 146.01 | 3.21 | **9.25** |
| **VDAG_10163** | *hypothetical protein* | 81.16 | 3.20 | **9.20** |
| **VDAG_00728** | *hypothetical protein* | 200.06 | 3.20 | **9.20** |
| **VDAG_08480** | *N-terminal amidase* | 326.80 | 3.20 | **9.19** |
| **VDAG_07057** | *acetyl-coenzyme A synthetase* | 932.89 | 3.19 | **9.13** |
| **VDAG_08474** | *hypothetical protein* | 376.67 | 3.19 | **9.11** |
| **VDAG_07373** | *serin endopeptidase* | 349.83 | 3.19 | **9.10** |
| **VDAG_05396** | *cellobiose dehydrogenase* | 66.68 | 3.19 | **9.10** |
| **VDAG_03686** | *lipase* | 404.80 | 3.18 | **9.08** |
| **VDAG_02710** | *high-affinity glucose transporter SNF3* | 1425.28 | 3.18 | **9.08** |
| **VDAG_08125** | *hypothetical protein* | 153.68 | 3.18 | **9.04** |
| **VDAG_04150** | *hypothetical protein* | 57.85 | 3.17 | **9.00** |
| **VDAG_05563** | *beta-mannosidase* | 78.05 | 3.16 | **8.92** |
| **VDAG_08143** | *3-carboxy-cis,cis-muconate cycloisomerase* | 26.43 | 3.15 | **8.90** |
| **VDAG_05052** | *hypothetical protein* | 755.68 | 3.15 | **8.88** |
| **VDAG_02763** | *pisatin demethylase* | 19.90 | 3.15 | **8.87** |
| **VDAG_03709** | *hypothetical protein* | 7.20 | 3.15 | **8.87** |
| **VDAG_07158** | *ECM14 protein* | 40.38 | 3.15 | **8.86** |
| **VDAG_05453** | *hypothetical protein* | 40.45 | 3.14 | **8.84** |
| **VDAG_01177** | *rhamnolipids biosynthesis 3-oxoacyl-[acyl-carrier-protein] reductase* | 61.84 | 3.14 | **8.82** |
| **VDAG_01932** | *norsolorinic acid reductase* | 560.27 | 3.14 | **8.80** |
| **VDAG_05055** | *hypothetical protein* | 30.99 | 3.14 | **8.80** |
| **VDAG_02676** | *cholinesterase* | 189.20 | 3.14 | **8.80** |
| **VDAG_05750** | *hypothetical protein* | 66.43 | 3.14 | **8.79** |
| **VDAG_04787** | *quinate permease* | 158.96 | 3.13 | **8.78** |
| **VDAG_07178** | *hypothetical protein* | 356.64 | 3.13 | **8.73** |
| **VDAG_09485** | *dehydrogenase/reductase SDR family member* | 82.27 | 3.11 | **8.66** |
| **VDAG_08205** | *myo-inositol 2-dehydrogenase* | 18.36 | 3.11 | **8.65** |
| **VDAG_08872** | *DUF636 domain-containing protein* | 1151.16 | 3.11 | **8.63** |
| **VDAG_08514** | *hypothetical protein* | 116.66 | 3.11 | **8.62** |
| **VDAG_05358** | *hypothetical protein* | 1607.77 | 3.11 | **8.62** |
| **VDAG_07114** | *cytochrome b2* | 81.96 | 3.11 | **8.61** |
| **VDAG_01149** | *FAD binding domain-containing protein* | 1612.37 | 3.11 | **8.61** |
| **VDAG_07495** | *serine/threonine-protein kinase SRPK3* | 32.24 | 3.10 | **8.60** |
| **VDAG_00335** | *secreted protein* | 100.14 | 3.10 | **8.55** |
| **VDAG_05254** | *DOC family protein* | 4.51 | 3.10 | **8.55** |
| **VDAG_06861** | *hypothetical protein* | 312.17 | 3.09 | **8.52** |
| **VDAG_03895** | *hypothetical protein* | 9.78 | 3.09 | **8.49** |
| **VDAG_01558** | *hypothetical protein* | 469.11 | 3.09 | **8.49** |
| **VDAG_02134** | *hypothetical protein* | 10.06 | 3.08 | **8.44** |
| **VDAG_09086** | *hypothetical protein* | 343.84 | 3.08 | **8.43** |
| **VDAG_00301** | *transcriptional activator protein acu-15* | 698.42 | 3.07 | **8.42** |
| **VDAG_00678** | *hypothetical protein* | 851.30 | 3.07 | **8.41** |
| **VDAG_09382** | *glucose oxidase* | 524.91 | 3.06 | **8.36** |
| **VDAG_02048** | *MFS transporter* | 9.81 | 3.06 | **8.36** |
| **VDAG_02114** | *4-coumarate-CoA ligase* | 19.46 | 3.06 | **8.36** |
| **VDAG_07157** | *hypothetical protein* | 747.74 | 3.06 | **8.35** |
| **VDAG_07660** | *beta-galactosidase* | 62.93 | 3.06 | **8.35** |
| **VDAG_06122** | *NmrA family protein* | 43.73 | 3.06 | **8.33** |
| **VDAG_04959** | *peroxisomal hydratase-dehydrogenase-epimerase* | 160.15 | 3.06 | **8.32** |
| **VDAG_03135** | *PRO41 protein* | 161.36 | 3.05 | **8.30** |
| **VDAG_09669** | *6-phosphogluconolactonase* | 108.91 | 3.05 | **8.29** |
| **VDAG_09106** | *alpha-N-arabinofuranosidase A* | 55.84 | 3.05 | **8.26** |
| **VDAG_04757** | *alpha-galactosidase* | 5.18 | 3.04 | **8.24** |
| **VDAG_03610** | *Tna1* | 151.54 | 3.04 | **8.21** |
| **VDAG_08024** | *allantoate permease* | 72.41 | 3.03 | **8.18** |
| **VDAG_05642** | *hypothetical protein* | 23.80 | 3.03 | **8.16** |
| **VDAG_05645** | *hypothetical protein* | 731.51 | 3.03 | **8.15** |
| **VDAG_07365** | *hypothetical protein* | 9.70 | 3.03 | **8.14** |
| **VDAG_03760** | *alpha-glucosides permease MPH2/3* | 60.66 | 3.03 | **8.14** |
| **VDAG_03437** | *hypothetical protein* | 23.14 | 3.02 | **8.13** |
| **VDAG_07362** | *high-affinity nicotinic acid transporter* | 146.85 | 3.02 | **8.12** |
| **VDAG_04905** | *ankyrin repeat protein* | 88.92 | 3.02 | **8.11** |
| **VDAG_08295** | *GTP cyclohydrolase II* | 1112.45 | 3.02 | **8.10** |
| **VDAG_03685** | *minor extracellular protease vpr* | 384.69 | 3.02 | **8.09** |
| **VDAG_05449** | *hypothetical protein* | 5.70 | 3.01 | **8.08** |
| **VDAG_04640** | *C6 zinc finger protein* | 663.52 | 3.01 | **8.08** |
| **VDAG_04148** | *hypothetical protein* | 49.94 | 3.01 | **8.08** |
| **VDAG_04429** | *hypothetical protein* | 213.53 | 3.01 | **8.07** |
| **VDAG_05979** | *alcohol dehydrogenase* | 69.83 | 3.01 | **8.05** |
| **VDAG_09191** | *hypothetical protein* | 144.98 | 3.01 | **8.04** |
| **VDAG_09533** | *hypothetical protein* | 1002.41 | 3.01 | **8.04** |
| **VDAG_10193** | *cellulose-binding family II* | 19.90 | 3.00 | **8.02** |
| **VDAG_04459** | *hypothetical protein* | 891.49 | 3.00 | **7.97** |
| **VDAG_05124** | *hypothetical protein* | 6.23 | 3.00 | **7.97** |
| **VDAG_09820** | *thymine dioxygenase* | 49.90 | 2.98 | **7.91** |
| **VDAG_08445** | *actin* | 5517.16 | 2.98 | **7.90** |
| **VDAG_08287** | *hypothetical protein* | 12.39 | 2.98 | **7.89** |
| **VDAG_00429** | *hypothetical protein* | 7.60 | 2.98 | **7.88** |
| **VDAG_01169** | *beta-xylosidase* | 150.42 | 2.98 | **7.88** |
| **VDAG_01580** | *fluconazole resistance protein* | 153.78 | 2.98 | **7.88** |
| **VDAG_06212** | *alpha/beta hydrolase* | 392.89 | 2.98 | **7.87** |
| **VDAG_02133** | *vitamin H transporter* | 144.07 | 2.97 | **7.84** |
| **VDAG_06890** | *SpvB domain-containing protein* | 19.46 | 2.97 | **7.82** |
| **VDAG_09646** | *hypothetical protein* | 5.46 | 2.97 | **7.82** |
| **VDAG_06297** | *hypothetical protein* | 74.45 | 2.97 | **7.81** |
| **VDAG_07359** | *hypothetical protein* | 102.48 | 2.97 | **7.81** |
| **VDAG_07738** | *glycerol kinase* | 441.69 | 2.96 | **7.77** |
| **VDAG_09706** | *polysaccharide deacetylase family protein* | 24.58 | 2.96 | **7.77** |
| **VDAG_01604** | *regulatory protein (transcription)* | 209.02 | 2.96 | **7.77** |
| **VDAG_00473** | *hypothetical protein* | 25.68 | 2.96 | **7.75** |
| **VDAG_02072** | *leucyl aminopeptidase* | 90.84 | 2.95 | **7.75** |
| **VDAG_06037** | *GPR1/FUN34/yaaH family protein* | 10941.70 | 2.95 | **7.75** |
| **VDAG_08071** | *hypothetical protein* | 7.57 | 2.95 | **7.73** |
| **VDAG_05818** | *retinol dehydrogenase* | 287.78 | 2.95 | **7.73** |
| **VDAG_03339** | *hypothetical protein* | 1559.94 | 2.95 | **7.73** |
| **VDAG_00583** | *hypothetical protein* | 9.23 | 2.95 | **7.72** |
| **VDAG_07293** | *hypothetical protein* | 113.98 | 2.93 | **7.61** |
| **VDAG_00600** | *acetyl esterase* | 433.49 | 2.92 | **7.59** |
| **VDAG_03364** | *serine/threonine protein kinase* | 1.23 | 2.92 | 7.57 |
| **VDAG_04483** | *cytochrome P450 52A11* | 183.57 | 2.92 | **7.55** |
| **VDAG_05357** | *FAD binding domain-containing protein* | 755.61 | 2.92 | **7.55** |
| **VDAG_02099** | *integral membrane protein* | 424.61 | 2.92 | **7.55** |
| **VDAG_05092** | *hypothetical protein* | 10.86 | 2.91 | **7.52** |
| **VDAG_07672** | *galactoside O-acetyltransferase* | 155.71 | 2.91 | **7.52** |
| **VDAG_05463** | *hypothetical protein* | 12.20 | 2.90 | **7.46** |
| **VDAG_07919** | *hypothetical protein* | 3.37 | 2.90 | **7.45** |
| **VDAG_07661** | *hypothetical protein* | 174.04 | 2.89 | **7.41** |
| **VDAG_06589** | *hypothetical protein* | 5.33 | 2.89 | **7.40** |
| **VDAG_08380** | *hypothetical protein* | 141.98 | 2.88 | **7.37** |
| **VDAG_09554** | *lysozyme* | 374.23 | 2.88 | **7.37** |
| **VDAG_04963** | *hypothetical protein* | 107.34 | 2.88 | **7.36** |
| **VDAG_02394** | *ankyrin repeat protein* | 1.76 | 2.86 | 7.28 |
| **VDAG_09878** | *hypothetical protein* | 136.43 | 2.86 | **7.28** |
| **VDAG_09288** | *arabinogalactan endo-1,4-beta-galactosidase* | 296.83 | 2.86 | **7.27** |
| **VDAG_07351** | *hypothetical protein* | 27.91 | 2.86 | **7.25** |
| **VDAG_09560** | *chitinase* | 21.30 | 2.86 | **7.25** |
| **VDAG_03340** | *hypothetical protein* | 4.05 | 2.86 | **7.25** |
| **VDAG_05506** | *hypothetical protein* | 3381.73 | 2.85 | **7.23** |
| **VDAG_05256** | *hypothetical protein* | 369.30 | 2.85 | **7.23** |
| **VDAG_03502** | *pyrroline-5-carboxylate reductase* | 36.50 | 2.85 | **7.22** |
| **VDAG_09289** | *hypothetical protein* | 104.79 | 2.85 | **7.22** |
| **VDAG_06146** | *hypothetical protein* | 20.59 | 2.85 | **7.21** |
| **VDAG_10298** | *hypothetical protein* | 9.99 | 2.85 | **7.20** |
| **VDAG_03323** | *aryl-alcohol dehydrogenase* | 2325.36 | 2.85 | **7.19** |
| **VDAG_00408** | *glucoamylase P* | 163.56 | 2.84 | **7.18** |
| **VDAG_07527** | *hypothetical protein* | 441.29 | 2.84 | **7.18** |
| **VDAG_03599** | *lactose permease* | 25.92 | 2.84 | **7.16** |
| **VDAG_07685** | *hypothetical protein* | 318.79 | 2.84 | **7.15** |
| **VDAG_05853** | *solute carrier family 35 member E3* | 327.03 | 2.84 | **7.14** |
| **VDAG_08237** | *fluconazole resistance protein* | 73.40 | 2.84 | **7.14** |
| **VDAG_09611** | *hypothetical protein* | 33.12 | 2.83 | **7.13** |
| **VDAG_02982** | *hypothetical protein* | 5.64 | 2.83 | **7.12** |
| **VDAG_05135** | *carboxypeptidase S1* | 14.06 | 2.83 | **7.11** |
| **VDAG_09087** | *hypothetical protein* | 69.31 | 2.83 | **7.11** |
| **VDAG_05148** | *hypothetical protein* | 3.65 | 2.83 | **7.10** |
| **VDAG_02776** | *hypothetical protein* | 315.46 | 2.83 | **7.10** |
| **VDAG_04337** | *hypothetical protein* | 156.27 | 2.83 | **7.09** |
| **VDAG_00833** | *thiol-specific monooxygenase* | 243.89 | 2.83 | **7.09** |
| **VDAG_05112** | *hypothetical protein* | 188.47 | 2.82 | **7.08** |
| **VDAG_05454** | *hypothetical protein* | 75.15 | 2.82 | **7.06** |
| **VDAG_08061** | *SGE1 protein* | 103.37 | 2.82 | **7.06** |
| **VDAG_05169** | *hypothetical protein* | 5.19 | 2.82 | **7.05** |
| **VDAG_01680** | *hypothetical protein* | 371.23 | 2.81 | **7.04** |
| **VDAG_08736** | *peptidoglycan binding domain-containing protein* | 168.50 | 2.81 | **7.03** |
| **VDAG_04555** | *hypothetical protein* | 115.42 | 2.81 | **7.02** |
| **VDAG_05823** | *hypothetical protein* | 51942.00 | 2.81 | **7.02** |
| **VDAG_09732** | *ubiquitin carboxyl-terminal hydrolase* | 27.32 | 2.81 | **7.02** |
| **VDAG_05824** | *DJ-1/PfpI family protein* | 280.30 | 2.81 | **7.02** |
| **VDAG_08263** | *thermostable beta-glucosidase B* | 267.81 | 2.81 | **7.01** |
| **VDAG_04667** | *hypothetical protein* | 286.82 | 2.81 | **7.01** |
| **VDAG_03553** | *alpha-N-arabinofuranosidase* | 49.57 | 2.81 | **7.00** |
| **VDAG_06769** | *hypothetical protein* | 222.74 | 2.81 | **7.00** |
| **VDAG_07927** | *hypothetical protein* | 14.17 | 2.81 | **6.99** |
| **VDAG_01203** | *benomyl/methotrexate resistance protein* | 100.79 | 2.81 | **6.99** |
| **VDAG_07643** | *hypothetical protein* | 209.87 | 2.80 | **6.99** |
| **VDAG_06739** | *hypothetical protein* | 312.50 | 2.80 | **6.97** |
| **VDAG_04701** | *necrosis-and ethylene-inducing protein and ethylene inducing peptide* | 162.72 | 2.80 | **6.94** |
| **VDAG_07422** | *hypothetical protein* | 12.82 | 2.79 | **6.94** |
| **VDAG_08086** | *vitamin H transporter 1* | 43.44 | 2.79 | **6.94** |
| **VDAG_03625** | *NAD-binding Rossmann fold oxidoreductase family protein* | 147.87 | 2.79 | **6.93** |
| **VDAG_09363** | *hypothetical protein* | 619.81 | 2.79 | **6.90** |
| **VDAG_01368** | *hypothetical protein* | 264.97 | 2.78 | **6.89** |
| **VDAG_04434** | *hypothetical protein* | 481.77 | 2.78 | **6.87** |
| **VDAG_05580** | *beta-glucosidase* | 90.55 | 2.78 | **6.86** |
| **VDAG_01549** | *hypothetical protein* | 1648.44 | 2.78 | **6.86** |
| **VDAG_02846** | *G-protein coupled receptor* | 45.39 | 2.78 | **6.85** |
| **VDAG_04671** | *amine oxidase B* | 28.21 | 2.77 | **6.84** |
| **VDAG_06251** | *hypothetical protein* | 22.14 | 2.77 | **6.83** |
| **VDAG_09153** | *hypothetical protein* | 1.23 | 2.77 | 6.83 |
| **VDAG_03814** | *phosphotransferase enzyme family protein* | 1.37 | 2.77 | 6.83 |
| **VDAG_03514** | *hypothetical protein* | 2.86 | 2.76 | **6.76** |
| **VDAG_03059** | *hypothetical protein* | 375.92 | 2.75 | **6.74** |
| **VDAG_00087** | *hypothetical protein* | 9.95 | 2.75 | **6.73** |
| **VDAG_02528** | *RNA-dependent RNA polymerase* | 141.41 | 2.75 | **6.73** |
| **VDAG_07741** | *bilirubin oxidase* | 38.43 | 2.74 | **6.69** |
| **VDAG_01156** | *hypothetical protein* | 120.59 | 2.74 | **6.68** |
| **VDAG_07980** | *aminopeptidase Y* | 604.51 | 2.74 | **6.67** |
| **VDAG_00576** | *hypothetical protein* | 144.83 | 2.73 | **6.65** |
| **VDAG_07758** | *HHE domain-containing protein* | 27.00 | 2.73 | **6.65** |
| **VDAG_07982** | *hypothetical protein* | 6.41 | 2.73 | **6.64** |
| **VDAG_09080** | *mitochondrial dicarboxylate transporter* | 130.75 | 2.73 | **6.62** |
| **VDAG_03810** | *hypothetical protein* | 82.02 | 2.72 | **6.59** |
| **VDAG_01338** | *hypothetical protein* | 124.39 | 2.72 | **6.58** |
| **VDAG_09335** | *protein kinase domain-containing protein* | 36.92 | 2.72 | **6.58** |
| **VDAG_03604** | *ABC transporter CDR4* | 50.69 | 2.72 | **6.58** |
| **VDAG_02714** | *RhiN protein* | 197.39 | 2.72 | **6.57** |
| **VDAG_07411** | *ankyrin and HET domain-containing protein* | 94.59 | 2.71 | **6.56** |
| **VDAG_07183** | *carboxypeptidase A* | 122.67 | 2.71 | **6.54** |
| **VDAG_00432** | *C6 zinc finger domain-containing protein* | 143.35 | 2.71 | **6.54** |
| **VDAG_04319** | *hypothetical protein* | 105.18 | 2.71 | **6.53** |
| **VDAG_07648** | *hypothetical protein* | 100.18 | 2.70 | **6.52** |
| **VDAG_07196** | *hypothetical protein* | 83.58 | 2.70 | **6.50** |
| **VDAG_02828** | *YkgB* | 347.44 | 2.70 | **6.50** |
| **VDAG_00805** | *neutral alpha-glucosidase ab* | 277.83 | 2.70 | **6.49** |
| **VDAG_05327** | *dienelactone hydrolase* | 876.77 | 2.70 | **6.49** |
| **VDAG_04758** | *hypothetical protein* | 2.84 | 2.70 | 6.49 |
| **VDAG_02239** | *high-affinity methionine permease* | 117.46 | 2.70 | **6.48** |
| **VDAG_10325** | *hypothetical protein* | 186.29 | 2.69 | **6.46** |
| **VDAG_10470** | *glucanase B* | 61.36 | 2.69 | **6.44** |
| **VDAG_07677** | *hypothetical protein* | 553.37 | 2.68 | **6.42** |
| **VDAG_03391** | *benzoate 4-monooxygenase cytochrome P450* | 5.78 | 2.68 | **6.40** |
| **VDAG_05820** | *hypothetical protein* | 241.12 | 2.68 | **6.40** |
| **VDAG_09192** | *hypothetical protein* | 74.81 | 2.68 | **6.39** |
| **VDAG_07518** | *hypothetical protein* | 14.02 | 2.67 | **6.38** |
| **VDAG_02743** | *beta-galactosidase* | 86.39 | 2.67 | **6.37** |
| **VDAG_05224** | *hypothetical protein* | 24.11 | 2.67 | **6.36** |
| **VDAG_01166** | *hypothetical protein* | 46.80 | 2.67 | **6.36** |
| **VDAG_04335** | *hypothetical protein* | 20.84 | 2.67 | 6.34 |
| **VDAG_05120** | *hypothetical protein* | 93.94 | 2.66 | **6.34** |
| **VDAG_05245** | *hypothetical protein* | 66.29 | 2.66 | **6.34** |
| **VDAG_01663** | *ent-kaurene oxidase* | 81.13 | 2.66 | **6.33** |
| **VDAG_05375** | *covalently-linked cell wall protein* | 60.81 | 2.66 | **6.31** |
| **VDAG_06168** | *hypothetical protein* | 3387.38 | 2.66 | **6.31** |
| **VDAG_05983** | *hypothetical protein* | 120.05 | 2.66 | **6.30** |
| **VDAG_02880** | *hypothetical protein* | 437.68 | 2.65 | **6.28** |
| **VDAG_00721** | *hypothetical protein* | 1278.41 | 2.65 | **6.28** |
| **VDAG_05338** | *hypothetical protein* | 564.73 | 2.65 | **6.26** |
| **VDAG_05800** | *hypothetical protein* | 10.23 | 2.64 | **6.25** |
| **VDAG_10471** | *hypothetical protein* | 37.79 | 2.64 | **6.23** |
| **VDAG_05644** | *hypothetical protein* | 36.58 | 2.64 | **6.22** |
| **VDAG_08816** | *verprolin* | 49.53 | 2.63 | **6.20** |
| **VDAG_02501** | *hypothetical protein* | 4.55 | 2.63 | **6.20** |
| **VDAG_07255** | *NAD-dependent epimerase/dehydratase* | 7.10 | 2.63 | **6.19** |
| **VDAG_07735** | *xanthine dehydrogenase* | 207.10 | 2.63 | **6.17** |
| **VDAG_07895** | *hypothetical protein* | 134.30 | 2.63 | **6.17** |
| **VDAG_10367** | *hypothetical protein* | 1467.03 | 2.63 | **6.17** |
| **VDAG_01659** | *ent-kaurene oxidase* | 74.06 | 2.62 | **6.16** |
| **VDAG_06425** | *hypothetical protein* | 294.69 | 2.62 | **6.14** |
| **VDAG_09562** | *hypothetical protein* | 179.49 | 2.62 | **6.14** |
| **VDAG_10074** | *tubulin beta chain* | 10391.08 | 2.62 | **6.13** |
| **VDAG_05324** | *3-alpha-(or 20-beta)-hydroxysteroid dehydrogenase* | 203.27 | 2.61 | **6.09** |
| **VDAG_05397** | *hypothetical protein* | 147.58 | 2.60 | **6.07** |
| **VDAG_10194** | *sodium/nucleoside cotransporter* | 449.80 | 2.60 | **6.07** |
| **VDAG_05339** | *3-hydroxyacyl-CoA dehydrogenase type-2* | 114.24 | 2.60 | **6.07** |
| **VDAG_08367** | *hypothetical protein* | 8.78 | 2.60 | **6.07** |
| **VDAG_10373** | *hypothetical protein* | 16.66 | 2.60 | **6.06** |
| **VDAG_00492** | *phosphorylcholine phosphatase* | 658.48 | 2.60 | **6.05** |
| **VDAG_01555** | *alpha-glucosidase* | 96.65 | 2.59 | **6.04** |
| **VDAG_00784** | *para-nitrobenzyl esterase* | 1639.50 | 2.59 | **6.01** |
| **VDAG_10161** | *hypothetical protein* | 19.40 | 2.59 | **6.01** |
| **VDAG_02142** | *hypothetical protein* | 11.44 | 2.59 | **6.00** |
| **VDAG_03410** | *hypothetical protein* | 50.36 | 2.58 | **5.99** |
| **VDAG_01683** | *hypothetical protein* | 32.71 | 2.58 | **5.98** |
| **VDAG_08703** | *alpha-1,2 mannosyltransferase KTR1* | 9.25 | 2.58 | **5.97** |
| **VDAG_01318** | *cutinase transcription factor 1 beta* | 262.13 | 2.58 | **5.97** |
| **VDAG_06611** | *C2H2 type zinc finger domain-containing protein* | 129.10 | 2.57 | **5.95** |
| **VDAG_04737** | *hypothetical protein* | 28.17 | 2.57 | **5.95** |
| **VDAG_08840** | *hypothetical protein* | 92.24 | 2.57 | **5.95** |
| **VDAG_06298** | *cAMP-independent regulatory protein pac2* | 338.80 | 2.57 | **5.94** |
| **VDAG_08146** | *3-ketoacyl-CoA thiolase* | 24.11 | 2.57 | **5.94** |
| **VDAG_07713** | *bifunctional P-450:NADPH-P450 reductase* | 455.11 | 2.57 | **5.93** |
| **VDAG_05379** | *maltose permease MAL31* | 922.22 | 2.57 | **5.93** |
| **VDAG_07670** | *alpha-ketoglutarate-dependent sulfonate dioxygenase* | 9.27 | 2.56 | **5.91** |
| **VDAG_09688** | *hypothetical protein* | 7.93 | 2.56 | **5.91** |
| **VDAG_02503** | *hypothetical protein* | 52.06 | 2.56 | **5.91** |
| **VDAG_04958** | *hypothetical protein* | 355.35 | 2.56 | **5.91** |
| **VDAG_04367** | *ADP-ribosylation factor* | 13782.35 | 2.56 | **5.90** |
| **VDAG_06014** | *HET domain-containing protein* | 24.82 | 2.55 | **5.87** |
| **VDAG_09681** | *hypothetical protein* | 5.61 | 2.55 | **5.87** |
| **VDAG_03763** | *high-affinity nicotinic acid transporter* | 93.43 | 2.55 | **5.86** |
| **VDAG_07344** | *cutinase* | 15.23 | 2.55 | **5.85** |
| **VDAG_05263** | *hypothetical protein* | 3.18 | 2.55 | 5.85 |
| **VDAG_01289** | *riboflavin transporter MCH5* | 513.77 | 2.55 | **5.84** |
| **VDAG_04781** | *LysM domain-containing protein* | 5.83 | 2.54 | **5.83** |
| **VDAG_10270** | *hypothetical protein* | 77.66 | 2.54 | **5.82** |
| **VDAG_01782** | *pectinesterase family protein* | 47.48 | 2.54 | **5.80** |
| **VDAG_09710** | *2-(R)-hydroxypropyl-CoM dehydrogenase* | 97.79 | 2.54 | **5.80** |
| **VDAG_09302** | *beta-xylosidase* | 198.67 | 2.53 | **5.78** |
| **VDAG_04806** | *hypothetical protein* | 49.88 | 2.53 | **5.77** |
| **VDAG_08087** | *hypothetical protein* | 22.27 | 2.53 | **5.77** |
| **VDAG_07948** | *hypothetical protein* | 68.15 | 2.53 | **5.77** |
| **VDAG_09708** | *alcohol dehydrogenase* | 17.53 | 2.53 | **5.76** |
| **VDAG_02844** | *ubiquitin carboxyl-terminal hydrolase* | 84.67 | 2.53 | **5.76** |
| **VDAG_03119** | *dimethylaniline monooxygenase* | 36.01 | 2.53 | **5.76** |
| **VDAG_03799** | *hypothetical protein* | 56.79 | 2.52 | **5.75** |
| **VDAG_02498** | *abscisic acid ABA receptor* | 62.41 | 2.52 | **5.75** |
| **VDAG_02105** | *hypothetical protein* | 7.07 | 2.52 | **5.75** |
| **VDAG_03855** | *hypothetical protein* | 82.96 | 2.52 | **5.73** |
| **VDAG_05981** | *hypothetical protein* | 124.88 | 2.52 | **5.72** |
| **VDAG_08199** | *hypothetical protein* | 4.32 | 2.51 | **5.71** |
| **VDAG_03188** | *hypothetical protein* | 7.91 | 2.51 | **5.70** |
| **VDAG_02357** | *PHO85 cyclin-1* | 0.86 | 2.51 | 5.68 |
| **VDAG_07369** | *peroxisomal copper amine oxidase* | 471.45 | 2.50 | **5.66** |
| **VDAG_06936** | *hypothetical protein* | 51.11 | 2.50 | **5.65** |
| **VDAG_08148** | *succinyl-CoA:3-ketoacid-coenzyme A transferase subunit A* | 18.94 | 2.50 | **5.65** |
| **VDAG_02347** | *hypothetical protein* | 3.09 | 2.50 | 5.64 |
| **VDAG_05992** | *exo-polygalacturonase* | 93.16 | 2.50 | **5.64** |
| **VDAG_09516** | *exoglucanase* | 86.51 | 2.49 | **5.63** |
| **VDAG_08283** | *hypothetical protein* | 0.58 | 2.49 | 5.61 |
| **VDAG_05982** | *hypothetical protein* | 151.22 | 2.49 | **5.61** |
| **VDAG_09940** | *hypothetical protein* | 145.90 | 2.48 | **5.60** |
| **VDAG_03903** | *general alpha-glucoside permease* | 21.19 | 2.48 | **5.59** |
| **VDAG_09928** | *ATP-binding cassette sub-family G member 2* | 42.67 | 2.48 | **5.57** |
| **VDAG_06920** | *hypothetical protein* | 1245.84 | 2.48 | **5.57** |
| **VDAG_01199** | *hypothetical protein* | 38.48 | 2.48 | **5.57** |
| **VDAG_08211** | *hypothetical protein* | 27.33 | 2.47 | **5.55** |
| **VDAG_03811** | *pantothenate transporter liz1* | 549.09 | 2.47 | **5.55** |
| **VDAG_07318** | *stress responsive A/B barrel domain-containing protein* | 61.24 | 2.47 | **5.55** |
| **VDAG_06252** | *exoglucanase-6A* | 149.59 | 2.47 | **5.54** |
| **VDAG_04692** | *2-(S)-hydroxypropyl-CoM dehydrogenase* | 11.39 | 2.47 | **5.54** |
| **VDAG_02984** | *secreted protein* | 1.82 | 2.47 | 5.53 |
| **VDAG_03161** | *hypothetical protein* | 1.82 | 2.47 | 5.53 |
| **VDAG_09869** | *hypothetical protein* | 59.55 | 2.47 | **5.52** |
| **VDAG_00623** | *hypothetical protein* | 102.16 | 2.46 | **5.51** |
| **VDAG_04915** | *transcription factor* | 9.16 | 2.46 | **5.51** |
| **VDAG_03617** | *riboflavin biosynthesis protein RibD C-terminal domain-containing protein* | 55.73 | 2.46 | **5.49** |
| **VDAG_07040** | *lipase/esterase* | 134.35 | 2.46 | **5.49** |
| **VDAG_07329** | *salicylate hydroxylase* | 839.70 | 2.45 | **5.47** |
| **VDAG_09849** | *hypothetical protein* | 145.62 | 2.45 | **5.47** |
| **VDAG_09655** | *hypothetical protein* | 16.78 | 2.45 | **5.47** |
| **VDAG_08615** | *isocitrate lyase* | 137.21 | 2.45 | **5.47** |
| **VDAG_08941** | *potassium transporter 1* | 322.31 | 2.45 | **5.46** |
| **VDAG_00741** | *hypothetical protein* | 45.92 | 2.45 | **5.45** |
| **VDAG_00587** | *hypothetical protein* | 84.13 | 2.45 | **5.45** |
| **VDAG_08047** | *hypothetical protein* | 66.44 | 2.44 | **5.44** |
| **VDAG_06971** | *hypothetical protein* | 129.33 | 2.44 | **5.44** |
| **VDAG_04979** | *secreted aspartic proteinase* | 991.93 | 2.44 | **5.42** |
| **VDAG_09340** | *hypothetical protein* | 2495.31 | 2.44 | **5.41** |
| **VDAG_09923** | *hypothetical protein* | 120.67 | 2.43 | **5.40** |
| **VDAG_00618** | *alcohol dehydrogenase* | 51.90 | 2.43 | **5.39** |
| **VDAG_07176** | *alkaline proteinase* | 148.73 | 2.43 | **5.38** |
| **VDAG_04149** | *NACHT and WD domain-containing protein* | 18.69 | 2.42 | **5.37** |
| **VDAG_00307** | *hypothetical protein* | 270.32 | 2.42 | **5.36** |
| **VDAG_00942** | *phospholipase* | 190.06 | 2.42 | **5.36** |
| **VDAG_07366** | *hypothetical protein* | 47.84 | 2.42 | **5.35** |
| **VDAG_09202** | *hypothetical protein* | 28.52 | 2.42 | **5.35** |
| **VDAG_09370** | *hypothetical protein* | 8.52 | 2.42 | **5.35** |
| **VDAG_10081** | *L-galactonate dehydratase* | 513.98 | 2.42 | **5.35** |
| **VDAG_09186** | *hypothetical protein* | 40.93 | 2.42 | **5.34** |
| **VDAG_00330** | *high-affinity nicotinic acid transporter* | 4.49 | 2.41 | **5.30** |
| **VDAG_08102** | *alcohol dehydrogenase* | 653.82 | 2.41 | **5.30** |
| **VDAG_03472** | *hypothetical protein* | 7.09 | 2.40 | **5.28** |
| **VDAG_05125** | *oligopeptide transporter 1* | 84.67 | 2.40 | **5.27** |
| **VDAG_09812** | *hypothetical protein* | 22.71 | 2.39 | **5.24** |
| **VDAG_09230** | *hypothetical protein* | 202.04 | 2.39 | **5.23** |
| **VDAG_08059** | *molybdopterin biosynthesis protein moeA* | 160.34 | 2.38 | **5.21** |
| **VDAG_04340** | *hypothetical protein* | 94.39 | 2.38 | **5.20** |
| **VDAG_03593** | *hypothetical protein* | 102.03 | 2.38 | **5.20** |
| **VDAG_08646** | *hypothetical protein* | 76.01 | 2.38 | **5.20** |
| **VDAG_03501** | *cellobiose dehydrogenase* | 42.34 | 2.38 | **5.19** |
| **VDAG_09325** | *lactose permease* | 468.26 | 2.38 | **5.19** |
| **VDAG_09074** | *hypothetical protein* | 459.04 | 2.38 | **5.19** |
| **VDAG_10499** | *hypothetical protein* | 94.12 | 2.38 | **5.19** |
| **VDAG_00674** | *ankyrin repeat and SAM domain-containing protein* | 25.50 | 2.38 | **5.19** |
| **VDAG_10290** | *hypothetical protein* | 18.08 | 2.37 | **5.18** |
| **VDAG_08738** | *hypothetical protein* | 6.72 | 2.37 | **5.17** |
| **VDAG_02164** | *lactose permease* | 64.01 | 2.37 | **5.16** |
| **VDAG_00351** | *hypothetical protein* | 3.51 | 2.36 | 5.13 |
| **VDAG_07254** | *voltage-gated potassium channel subunit beta-1* | 7.04 | 2.36 | **5.12** |
| **VDAG_02857** | *hypothetical protein* | 43.12 | 2.35 | **5.11** |
| **VDAG_04736** | *hypothetical protein* | 3.08 | 2.35 | 5.11 |
| **VDAG_05595** | *ubiquitin* | 143489.18 | 2.35 | **5.10** |
| **VDAG_10297** | *peptidase M28* | 171.39 | 2.35 | **5.09** |
| **VDAG_09262** | *hypothetical protein* | 2.81 | 2.35 | 5.09 |
| **VDAG_08288** | *catechol O-methyltransferase* | 2.56 | 2.34 | 5.06 |
| **VDAG_10324** | *hypothetical protein* | 71.08 | 2.34 | **5.06** |
| **VDAG_00763** | *carnitinyl-CoA dehydratase* | 12.64 | 2.34 | **5.06** |
| **VDAG_09055** | *hypothetical protein* | 2228.61 | 2.34 | **5.05** |
| **VDAG_01077** | *hypothetical protein* | 46.82 | 2.34 | **5.05** |
| **VDAG_04706** | *hypothetical protein* | 19.86 | 2.33 | **5.04** |
| **VDAG_01355** | *hypothetical protein* | 6.60 | 2.33 | **5.04** |
| **VDAG_07659** | *hypothetical protein* | 18.18 | 2.33 | **5.03** |
| **VDAG_07446** | *phosphoenolpyruvate carboxykinase* | 6426.47 | 2.33 | **5.03** |
| **VDAG_09403** | *hypothetical protein* | 11.63 | 2.33 | **5.02** |
| **VDAG_07444** | *RanGTP-binding protein* | 161.98 | 2.33 | **5.02** |
| **VDAG_09132** | *hypothetical protein* | 5.79 | 2.33 | **5.02** |
| **VDAG_04030** | *hypothetical protein* | 1.83 | 2.33 | 5.02 |
| **VDAG_07487** | *hypothetical protein* | 29.46 | 2.32 | **5.01** |
| **VDAG_10120** | *hypothetical protein* | 39.54 | 2.32 | **5.00** |
| **VDAG_07701** | *hypothetical protein* | 969.68 | 2.32 | **5.00** |

**Table S4.** List of down-regulated fungal genes in *A. thaliana* (ATH) and *V. dahliae* (VDA) co-culture compared to *V. dahliae* grown separately. Cut-off was made at a log_2_-fold change of -1.33.

| **GeneID** | **Symbol** | **base Mean** | **log2 Fold Change** | **Fold Change** |
| --- | --- | --- | --- | --- |
| **VDAG_01183** | *hypothetical protein* | 830.49 | -7.95 | **-246.83** |
| **VDAG_04417** | *hypothetical protein* | 1477.05 | -7.92 | **-241.55** |
| **VDAG_02257** | *xylulose-5-phosphate phosphoketolase* | 9492.47 | -7.52 | **-183.72** |
| **VDAG_01185** | *hypothetical protein* | 224.94 | -7.32 | **-159.30** |
| **VDAG_09524** | *dibenzothiophene desulfurization enzyme C* | 5711.77 | -7.29 | **-156.42** |
| **VDAG_08137** | *hypothetical protein* | 1457.99 | -6.92 | **-121.09** |
| **VDAG_00299** | *hypothetical protein* | 8564.18 | -6.92 | **-120.70** |
| **VDAG_10189** | *hypothetical protein* | 5503.22 | -6.81 | **-112.49** |
| **VDAG_02849** | *polyamine transporter 3* | 961.62 | -6.78 | **-110.14** |
| **VDAG_06151** | *high affinity copper transporter* | 2708.21 | -6.77 | **-109.32** |
| **VDAG_07939** | *hypothetical protein* | 5312.84 | -6.53 | **-92.73** |
| **VDAG_08136** | *hypothetical protein* | 1029.37 | -6.51 | **-91.04** |
| **VDAG_04718** | *pectate lyase B* | 4502.12 | -6.34 | **-80.80** |
| **VDAG_01194** | *polyamine transporter 2* | 247.23 | -6.33 | **-80.33** |
| **VDAG_07973** | *hypothetical protein* | 220.91 | -6.31 | **-79.17** |
| **VDAG_03216** | *hypothetical protein* | 755.28 | -6.31 | **-79.15** |
| **VDAG_06572** | *sterol 24-C-methyltransferase* | 1317.03 | -6.09 | **-68.02** |
| **VDAG_07346** | *hypothetical protein* | 323.77 | -6.07 | **-67.08** |
| **VDAG_06199** | *SnodProt1* | 5613.07 | -6.04 | **-65.98** |
| **VDAG_09443** | *pyruvate decarboxylase* | 33566.19 | -6.03 | **-65.57** |
| **VDAG_10190** | *hypothetical protein* | 482.11 | -6.02 | **-65.01** |
| **VDAG_04907** | *hypothetical protein* | 455.94 | -5.98 | **-63.21** |
| **VDAG_07715** | *glucan 1,4-alpha-maltohexaosidase* | 2726.65 | -5.98 | **-63.08** |
| **VDAG_08797** | *30 kDa heat shock protein* | 6559.17 | -5.97 | **-62.88** |
| **VDAG_07587** | *2,5-diketo-D-gluconic acid reductase A* | 77.27 | -5.97 | **-62.57** |
| **VDAG_09149** | *hypothetical protein* | 1781.23 | -5.80 | **-55.73** |
| **VDAG_00852** | *tubulin-specific chaperone C* | 2170.00 | -5.73 | **-53.06** |
| **VDAG_09303** | *NADPH dehydrogenase* | 2310.74 | -5.69 | **-51.64** |
| **VDAG_09522** | *aquaporin-9* | 2244.58 | -5.67 | **-51.07** |
| **VDAG_01226** | *coproporphyrinogen III oxidase* | 26858.51 | -5.62 | **-49.13** |
| **VDAG_07940** | *beta-lactamase family protein* | 2412.61 | -5.59 | **-48.16** |
| **VDAG_01379** | *hypothetical protein* | 1458.85 | -5.56 | **-47.16** |
| **VDAG_01184** | *hypothetical protein* | 121.21 | -5.40 | **-42.33** |
| **VDAG_07396** | *hypothetical protein* | 1033.96 | -5.32 | **-40.07** |
| **VDAG_04908** | *hypothetical protein* | 49.85 | -5.32 | **-40.01** |
| **VDAG_02468** | *hypothetical protein* | 535.04 | -5.28 | **-38.91** |
| **VDAG_09805** | *siderophore iron transporter mirB* | 588.33 | -5.27 | **-38.49** |
| **VDAG_06698** | *hypothetical protein* | 435.31 | -5.19 | **-36.45** |
| **VDAG_02103** | *hypothetical protein* | 359.91 | -5.19 | **-36.44** |
| **VDAG_02045** | *high affinity copper transporter* | 1604.47 | -5.17 | **-36.00** |
| **VDAG_04297** | *hypothetical protein* | 1089.42 | -5.17 | **-35.95** |
| **VDAG_06038** | *osmotic growth protein* | 1766.92 | -5.14 | **-35.17** |
| **VDAG_03209** | *alternative oxidase* | 2893.95 | -5.13 | **-35.02** |
| **VDAG_04349** | *J-type co-chaperone JAC1* | 187.37 | -5.02 | **-32.55** |
| **VDAG_08712** | *cyanide hydratase* | 4600.65 | -4.98 | **-31.51** |
| **VDAG_04906** | *hypothetical protein* | 33.41 | -4.92 | **-30.23** |
| **VDAG_06328** | *hypothetical protein* | 798.35 | -4.89 | **-29.66** |
| **VDAG_06900** | *hypothetical protein* | 688.29 | -4.84 | **-28.73** |
| **VDAG_05174** | *hypothetical protein* | 103.90 | -4.84 | **-28.55** |
| **VDAG_10220** | *hemoglobin* | 1571.69 | -4.83 | **-28.43** |
| **VDAG_07173** | *conidiation-specific protein* | 127.69 | -4.82 | **-28.34** |
| **VDAG_04365** | *heat shock protein* | 7875.58 | -4.81 | **-27.99** |
| **VDAG_04793** | *alcohol dehydrogenase* | 1820.83 | -4.76 | **-27.18** |
| **VDAG_02943** | *MNNG and nitrosoguanidine resistance protein* | 442.28 | -4.75 | **-26.99** |
| **VDAG_06183** | *hemoglobin* | 507.42 | -4.69 | **-25.82** |
| **VDAG_05443** | *galactose transporter* | 949.73 | -4.62 | **-24.65** |
| **VDAG_05796** | *hypothetical protein* | 63.12 | -4.57 | **-23.68** |
| **VDAG_01705** | *NmrA family protein* | 1221.53 | -4.55 | **-23.35** |
| **VDAG_00005** | *phenazine biosynthesis PhzC/PhzF protein* | 854.53 | -4.46 | **-22.05** |
| **VDAG_00511** | *glucan 1,3-beta-glucosidase* | 491.92 | -4.44 | **-21.67** |
| **VDAG_03732** | *hypothetical protein* | 68.40 | -4.43 | **-21.56** |
| **VDAG_03345** | *succinate-semialdehyde dehydrogenase* | 163.03 | -4.41 | **-21.20** |
| **VDAG_00409** | *C6 zinc finger domain-containing protein* | 1758.54 | -4.40 | **-21.13** |
| **VDAG_04571** | *4-aminobutyrate aminotransferase* | 963.36 | -4.38 | **-20.85** |
| **VDAG_07229** | *hypothetical protein* | 1205.51 | -4.36 | **-20.52** |
| **VDAG_05829** | *heat shock protein HSP98* | 4504.00 | -4.31 | **-19.86** |
| **VDAG_03922** | *4-hydroxyphenylpyruvate dioxygenase* | 944.28 | -4.30 | **-19.72** |
| **VDAG_05386** | *amino acid transporter* | 122.44 | -4.28 | **-19.47** |
| **VDAG_01676** | *hypothetical protein* | 19.39 | -4.28 | **-19.39** |
| **VDAG_09777** | *FAD binding domain-containing protein* | 466.02 | -4.25 | **-18.96** |
| **VDAG_09644** | *arginine permease* | 167.04 | -4.23 | **-18.79** |
| **VDAG_08179** | *multidrug transporter* | 282.28 | -4.22 | **-18.69** |
| **VDAG_09857** | *hypothetical protein* | 375.74 | -4.22 | **-18.64** |
| **VDAG_10056** | *3-methyl-2-oxobutanoate hydroxymethyltransferase* | 2473.42 | -4.13 | **-17.47** |
| **VDAG_05905** | *hypothetical protein* | 46.44 | -4.12 | **-17.35** |
| **VDAG_01467** | *glucose-repressible protein* | 3024.94 | -4.08 | **-16.86** |
| **VDAG_01692** | *endoglucanase* | 309.88 | -4.03 | **-16.28** |
| **VDAG_07414** | *homogentisate 1,2-dioxygenase* | 1212.03 | -4.02 | **-16.27** |
| **VDAG_06175** | *phenylacetone monooxygenase* | 27.04 | -3.99 | **-15.84** |
| **VDAG_00437** | *hypothetical protein* | 663.76 | -3.95 | **-15.44** |
| **VDAG_09785** | *hypothetical protein* | 14.90 | -3.93 | **-15.29** |
| **VDAG_07201** | *hypothetical protein* | 837.33 | -3.93 | **-15.22** |
| **VDAG_09432** | *hypothetical protein* | 341.27 | -3.90 | **-14.92** |
| **VDAG_03676** | *hypothetical protein* | 25.25 | -3.90 | **-14.89** |
| **VDAG_03785** | *hypothetical protein* | 382.17 | -3.86 | **-14.50** |
| **VDAG_09801** | *siderophore iron transporter mirB* | 568.98 | -3.84 | **-14.31** |
| **VDAG_04572** | *succinate-semialdehyde dehydrogenase* | 165.52 | -3.82 | **-14.14** |
| **VDAG_00482** | *hypothetical protein* | 1399.75 | -3.81 | **-14.07** |
| **VDAG_02238** | *hypothetical protein* | 350.58 | -3.81 | **-14.03** |
| **VDAG_04712** | *hypothetical protein* | 36.32 | -3.78 | **-13.73** |
| **VDAG_00706** | *bli-3* | 946.20 | -3.77 | **-13.60** |
| **VDAG_09444** | *FAD-linked sulfhydryl oxidase ALR* | 1172.30 | -3.76 | **-13.58** |
| **VDAG_01832** | *hypothetical protein* | 11.97 | -3.74 | **-13.40** |
| **VDAG_00938** | *hypothetical protein* | 206.43 | -3.74 | **-13.36** |
| **VDAG_00660** | *hypothetical protein* | 21.58 | -3.74 | **-13.32** |
| **VDAG_08193** | *hypothetical protein* | 5572.26 | -3.70 | **-13.04** |
| **VDAG_02244** | *hypothetical protein* | 192.29 | -3.70 | **-13.01** |
| **VDAG_03764** | *hypothetical protein* | 86.69 | -3.70 | **-12.98** |
| **VDAG_06021** | *retinol dehydrogenase* | 91.60 | -3.68 | **-12.84** |
| **VDAG_04464** | *benzodiazepine receptor family protein* | 714.69 | -3.67 | **-12.69** |
| **VDAG_06161** | *glucokinase* | 33.06 | -3.66 | **-12.63** |
| **VDAG_09898** | *AN1-type zinc finger protein* | 1140.25 | -3.63 | **-12.38** |
| **VDAG_08267** | *hypothetical protein* | 1716.93 | -3.63 | **-12.38** |
| **VDAG_03780** | *clock-controlled-9 protein* | 1137.34 | -3.62 | **-12.33** |
| **VDAG_01812** | *hypothetical protein* | 108.86 | -3.62 | **-12.31** |
| **VDAG_08489** | *psi1* | 4465.86 | -3.58 | **-11.96** |
| **VDAG_00282** | *hypothetical protein* | 30.95 | -3.58 | **-11.94** |
| **VDAG_07230** | *superoxide dismutase* | 621.06 | -3.57 | **-11.88** |
| **VDAG_06159** | *glucosamine-6-phosphate deaminase* | 224.09 | -3.57 | **-11.86** |
| **VDAG_02150** | *arginase* | 834.75 | -3.56 | **-11.77** |
| **VDAG_10037** | *SILG protein* | 1218.79 | -3.55 | **-11.71** |
| **VDAG_04083** | *hypothetical protein* | 46.83 | -3.54 | **-11.59** |
| **VDAG_05554** | *arrestin domain-containing protein* | 1177.16 | -3.53 | **-11.55** |
| **VDAG_08931** | *C-4 methylsterol oxidase* | 3585.13 | -3.52 | **-11.51** |
| **VDAG_06314** | *C-5 sterol desaturase* | 83.89 | -3.51 | **-11.42** |
| **VDAG_04010** | *hypothetical protein* | 174.18 | -3.49 | **-11.27** |
| **VDAG_09301** | *calcium-transporting ATPase* | 1045.40 | -3.49 | **-11.27** |
| **VDAG_04825** | *S-(hydroxymethyl)glutathione dehydrogenase* | 103.99 | -3.49 | **-11.26** |
| **VDAG_02216** | *hypothetical protein* | 188.06 | -3.48 | **-11.19** |
| **VDAG_09803** | *hypothetical protein* | 117.57 | -3.48 | **-11.19** |
| **VDAG_06094** | *mitochondrial hypoxia responsive domain-containing protein* | 5299.30 | -3.48 | **-11.12** |
| **VDAG_09965** | *CCR4-Not complex subunit Caf16* | 900.71 | -3.45 | **-10.96** |
| **VDAG_01870** | *hypothetical protein* | 37.36 | -3.45 | **-10.95** |
| **VDAG_10084** | *hypothetical protein* | 1391.83 | -3.45 | **-10.94** |
| **VDAG_03354** | *pectate lyase* | 9.45 | -3.45 | **-10.90** |
| **VDAG_02057** | *fumarylacetoacetase* | 1094.32 | -3.43 | **-10.81** |
| **VDAG_04771** | *hypothetical protein* | 58.42 | -3.42 | **-10.69** |
| **VDAG_00530** | *cysteine dioxygenase* | 6766.43 | -3.42 | **-10.67** |
| **VDAG_04910** | *hypothetical protein* | 9.42 | -3.42 | **-10.67** |
| **VDAG_09148** | *hypothetical protein* | 101.56 | -3.41 | **-10.61** |
| **VDAG_08579** | *hypothetical protein* | 1703.17 | -3.41 | **-10.60** |
| **VDAG_01109** | *hypothetical protein* | 2372.97 | -3.39 | **-10.49** |
| **VDAG_08114** | *exostosin-2* | 175.88 | -3.39 | **-10.48** |
| **VDAG_07718** | *2-hydroxyacid dehydrogenase* | 26.22 | -3.38 | **-10.43** |
| **VDAG_09377** | *hypothetical protein* | 81.20 | -3.37 | **-10.37** |
| **VDAG_08138** | *hypothetical protein* | 53.85 | -3.37 | **-10.36** |
| **VDAG_07579** | *SH3 domain-containing protein* | 278.03 | -3.35 | **-10.20** |
| **VDAG_01409** | *hypothetical protein* | 330.52 | -3.35 | **-10.16** |
| **VDAG_03116** | *cytochrome c peroxidase* | 1501.12 | -3.34 | **-10.16** |
| **VDAG_02734** | *hypothetical protein* | 236.50 | -3.34 | **-10.13** |
| **VDAG_00528** | *endoglucanase II* | 16.48 | -3.32 | **-9.97** |
| **VDAG_06764** | *FDD123 protein* | 2734.33 | -3.32 | **-9.96** |
| **VDAG_07577** | *hypothetical protein* | 42.59 | -3.30 | **-9.87** |
| **VDAG_05117** | *surface protein* | 8329.79 | -3.30 | **-9.86** |
| **VDAG_07808** | *hypothetical protein* | 26.53 | -3.30 | **-9.86** |
| **VDAG_07277** | *hypothetical protein* | 43.32 | -3.30 | **-9.85** |
| **VDAG_03554** | *hypothetical protein* | 9.03 | -3.29 | **-9.81** |
| **VDAG_05666** | *hypothetical protein* | 426.23 | -3.29 | **-9.78** |
| **VDAG_01291** | *hypothetical protein* | 668.88 | -3.27 | **-9.67** |
| **VDAG_03867** | *multidrug resistance protein mdtG* | 14.86 | -3.24 | **-9.46** |
| **VDAG_02708** | *hypothetical protein* | 118.29 | -3.24 | **-9.46** |
| **VDAG_01834** | *UDP-glucosyl transferase family protein* | 31.97 | -3.24 | **-9.43** |
| **VDAG_03567** | *hypothetical protein* | 24.07 | -3.24 | **-9.43** |
| **VDAG_09793** | *lactate 2-monooxygenase* | 8.18 | -3.22 | **-9.31** |
| **VDAG_05491** | *copper-transporting ATPase* | 396.75 | -3.19 | **-9.10** |
| **VDAG_01811** | *phosphopantothenate-cysteine ligase* | 222.27 | -3.17 | **-8.98** |
| **VDAG_01312** | *hypothetical protein* | 1312.49 | -3.17 | **-8.98** |
| **VDAG_00480** | *hypothetical protein* | 21.51 | -3.17 | **-8.97** |
| **VDAG_07952** | *dual specificity phosphatase* | 1317.48 | -3.16 | **-8.95** |
| **VDAG_05971** | *hypothetical protein* | 15.56 | -3.15 | **-8.86** |
| **VDAG_02508** | *hypothetical protein* | 83.15 | -3.15 | **-8.85** |
| **VDAG_07278** | *hypothetical protein* | 8.33 | -3.14 | **-8.79** |
| **VDAG_06775** | *tRNA (uracil-5-)-methyltransferase TRM9* | 83.39 | -3.13 | **-8.77** |
| **VDAG_06513** | *6-phosphogluconolactonase* | 292.93 | -3.13 | **-8.77** |
| **VDAG_01503** | *hypothetical protein* | 564.25 | -3.13 | **-8.74** |
| **VDAG_09592** | *betaine aldehyde dehydrogenase* | 764.13 | -3.12 | **-8.72** |
| **VDAG_01845** | *branched-chain-amino-acid aminotransferase* | 213.31 | -3.12 | **-8.70** |
| **VDAG_10185** | *pantothenate synthetase* | 197.67 | -3.12 | **-8.69** |
| **VDAG_03694** | *acetylcholinesterase* | 84.43 | -3.12 | **-8.67** |
| **VDAG_05481** | *2-oxoisovalerate dehydrogenase subunit alpha* | 361.67 | -3.11 | **-8.62** |
| **VDAG_06200** | *hypothetical protein* | 1033.41 | -3.11 | **-8.61** |
| **VDAG_06478** | *hypothetical protein* | 1392.26 | -3.10 | **-8.58** |
| **VDAG_01922** | *isovaleryl-CoA dehydrogenase* | 230.45 | -3.08 | **-8.48** |
| **VDAG_05352** | *hypothetical protein* | 176.23 | -3.07 | **-8.39** |
| **VDAG_01934** | *squalene monooxygenase* | 343.78 | -3.05 | **-8.27** |
| **VDAG_07212** | *hypothetical protein* | 489.38 | -3.05 | **-8.27** |
| **VDAG_09963** | *hypothetical protein* | 796.17 | -3.04 | **-8.25** |
| **VDAG_02173** | *hypothetical protein* | 4652.27 | -3.03 | **-8.17** |
| **VDAG_07101** | *hypothetical protein* | 1251.61 | -3.03 | **-8.17** |
| **VDAG_01666** | *hypothetical protein* | 350.35 | -3.02 | **-8.13** |
| **VDAG_02204** | *hypothetical protein* | 706.05 | -3.00 | **-8.01** |
| **VDAG_02129** | *hypothetical protein* | 20.21 | -3.00 | **-8.01** |
| **VDAG_04516** | *fumarylacetoacetate hydrolase domain-containing protein 2A* | 1546.68 | -3.00 | **-8.00** |
| **VDAG_05023** | *hypothetical protein* | 12.69 | -3.00 | **-8.00** |
| **VDAG_07018** | *inositolphosphorylceramide-B C-26 hydroxylase* | 2789.97 | -3.00 | **-7.97** |
| **VDAG_04732** | *aromatic and neutral aliphatic amino acid permease* | 1089.96 | -2.99 | **-7.94** |
| **VDAG_08444** | *Hsp70 nucleotide exchange factor FES1* | 574.63 | -2.98 | **-7.88** |
| **VDAG_03267** | *hypothetical protein* | 139.89 | -2.97 | **-7.83** |
| **VDAG_04686** | *phenylacetone monooxygenase* | 95.19 | -2.97 | **-7.83** |
| **VDAG_07227** | *plasma membrane iron permease* | 729.16 | -2.97 | **-7.82** |
| **VDAG_03963** | *short-chain-fatty-acid-CoA ligase* | 102.46 | -2.95 | **-7.75** |
| **VDAG_03920** | *hypothetical protein* | 681.76 | -2.95 | **-7.72** |
| **VDAG_08768** | *protein phosphatase methylesterase* | 115.06 | -2.94 | **-7.65** |
| **VDAG_06870** | *D-lactate dehydrogenase* | 1005.57 | -2.93 | **-7.63** |
| **VDAG_00975** | *IBR domain-containing protein* | 6.19 | -2.92 | **-7.56** |
| **VDAG_01492** | *mlo2* | 492.28 | -2.91 | **-7.54** |
| **VDAG_03582** | *L-xylulose reductase* | 1544.63 | -2.91 | **-7.54** |
| **VDAG_03447** | *adiponectin receptor protein* | 362.13 | -2.91 | **-7.53** |
| **VDAG_07717** | *hypothetical protein* | 6.34 | -2.91 | **-7.52** |
| **VDAG_02724** | *hypothetical protein* | 166.99 | -2.90 | **-7.49** |
| **VDAG_07340** | *hypothetical protein* | 1254.96 | -2.90 | **-7.47** |
| **VDAG_06974** | *hypothetical protein* | 296.81 | -2.90 | **-7.46** |
| **VDAG_08788** | *hypothetical protein* | 49.88 | -2.90 | **-7.45** |
| **VDAG_02215** | *endoglucanase-1* | 1409.59 | -2.89 | **-7.40** |
| **VDAG_09297** | *O-acetylhomoserine (thiol)-lyase* | 114.05 | -2.89 | **-7.40** |
| **VDAG_04291** | *hypothetical protein* | 782.43 | -2.89 | **-7.39** |
| **VDAG_05016** | *PQ loop repeat protein* | 66.46 | -2.89 | **-7.39** |
| **VDAG_00478** | *pyridoxamine 5'-phosphate oxidase* | 902.83 | -2.88 | **-7.37** |
| **VDAG_08265** | *aspergillopepsin F* | 1034.26 | -2.87 | **-7.32** |
| **VDAG_07100** | *hypothetical protein* | 204.58 | -2.87 | **-7.32** |
| **VDAG_02833** | *hypothetical protein* | 45.38 | -2.86 | **-7.28** |
| **VDAG_02921** | *hypothetical protein* | 106.15 | -2.86 | **-7.26** |
| **VDAG_06952** | *hypothetical protein* | 397.09 | -2.86 | **-7.25** |
| **VDAG_02922** | *hypothetical protein* | 112.41 | -2.85 | **-7.20** |
| **VDAG_00958** | *hypothetical protein* | 55.83 | -2.85 | **-7.19** |
| **VDAG_07695** | *chorismate synthase* | 246.23 | -2.84 | **-7.18** |
| **VDAG_08580** | *hypothetical protein* | 363.70 | -2.84 | **-7.18** |
| **VDAG_07129** | *hypothetical protein* | 25.44 | -2.83 | **-7.12** |
| **VDAG_03378** | *hypothetical protein* | 102.30 | -2.82 | **-7.08** |
| **VDAG_07279** | *hypothetical protein* | 53.46 | -2.82 | **-7.05** |
| **VDAG_05916** | *hypothetical protein* | 672.79 | -2.82 | **-7.04** |
| **VDAG_05578** | *hypothetical protein* | 34.36 | -2.82 | **-7.04** |
| **VDAG_09133** | *hypothetical protein* | 5.83 | -2.81 | **-7.02** |
| **VDAG_01819** | *glutamate decarboxylase* | 10662.91 | -2.81 | **-7.02** |
| **VDAG_02545** | *hypothetical protein* | 301.12 | -2.80 | **-6.97** |
| **VDAG_08248** | *hypothetical protein* | 254.37 | -2.79 | **-6.92** |
| **VDAG_08354** | *hypothetical protein* | 10.69 | -2.78 | **-6.88** |
| **VDAG_08063** | *ribose-5-phosphate isomerase* | 1082.38 | -2.77 | **-6.84** |
| **VDAG_04032** | *hypothetical protein* | 25.65 | -2.77 | **-6.82** |
| **VDAG_05087** | *NADPH-dependent D-xylose reductase II,III* | 17.93 | -2.76 | **-6.78** |
| **VDAG_03932** | *alkanesulfonate monooxygenase* | 1536.68 | -2.76 | **-6.77** |
| **VDAG_08413** | *hypothetical protein* | 91.61 | -2.75 | **-6.75** |
| **VDAG_07211** | *pyrimidine precursor biosynthesis enzyme THI11* | 5849.60 | -2.75 | **-6.74** |
| **VDAG_04794** | *LolT* | 231.63 | -2.75 | **-6.73** |
| **VDAG_03376** | *hypothetical protein* | 29.27 | -2.75 | **-6.72** |
| **VDAG_00954** | *hypothetical protein* | 5.61 | -2.75 | **-6.71** |
| **VDAG_07042** | *polyamine transporter 1* | 390.53 | -2.74 | **-6.70** |
| **VDAG_04833** | *endochitinase* | 5.87 | -2.74 | **-6.70** |
| **VDAG_10229** | *saccharopine dehydrogenase* | 603.09 | -2.74 | **-6.68** |
| **VDAG_06343** | *5-aminolevulinate synthase* | 164.39 | -2.73 | **-6.66** |
| **VDAG_04863** | *hypothetical protein* | 6.00 | -2.73 | **-6.65** |
| **VDAG_09376** | *hypothetical protein* | 332.01 | -2.73 | **-6.64** |
| **VDAG_00570** | *hypothetical protein* | 537.05 | -2.71 | **-6.56** |
| **VDAG_03550** | *dimethylaniline monooxygenase* | 199.57 | -2.71 | **-6.54** |
| **VDAG_04455** | *hypothetical protein* | 13141.06 | -2.71 | **-6.53** |
| **VDAG_07348** | *nucleoside-diphosphate-sugar epimerase family protein* | 249.09 | -2.71 | **-6.53** |
| **VDAG_01298** | *hypothetical protein* | 10.83 | -2.71 | **-6.52** |
| **VDAG_01825** | *ubiquinone biosynthesis protein CAT5* | 73.92 | -2.70 | **-6.51** |
| **VDAG_03931** | *GTP-binding protein YPTC4* | 156.92 | -2.70 | **-6.51** |
| **VDAG_07972** | *hypothetical protein* | 10.03 | -2.70 | **-6.51** |
| **VDAG_07083** | *H+/hexose cotransporter 1* | 22.72 | -2.70 | **-6.50** |
| **VDAG_01874** | *hypothetical protein* | 43.18 | -2.70 | **-6.49** |
| **VDAG_01036** | *hypothetical protein* | 3523.26 | -2.69 | **-6.47** |
| **VDAG_01852** | *SnodProt1* | 130.53 | -2.69 | **-6.46** |
| **VDAG_02884** | *hypothetical protein* | 8241.00 | -2.69 | **-6.46** |
| **VDAG_05241** | *hypothetical protein* | 22.10 | -2.69 | **-6.46** |
| **VDAG_08711** | *hypothetical protein* | 603.78 | -2.69 | **-6.46** |
| **VDAG_01575** | *2-oxoisovalerate dehydrogenase subunit beta* | 205.50 | -2.67 | **-6.38** |
| **VDAG_00658** | *hypothetical protein* | 151.39 | -2.67 | **-6.38** |
| **VDAG_05331** | *acid phosphatase* | 125.71 | -2.67 | **-6.37** |
| **VDAG_06106** | *endoplasmic oxidoreductin-1* | 1226.62 | -2.67 | **-6.36** |
| **VDAG_04348** | *DJ-1/PfpI family protein* | 93.03 | -2.66 | **-6.30** |
| **VDAG_04418** | *ran GTPase-activating protein* | 1224.17 | -2.65 | **-6.30** |
| **VDAG_07590** | *hypothetical protein* | 10.10 | -2.65 | **-6.28** |
| **VDAG_07420** | *hypothetical protein* | 95.44 | -2.65 | **-6.28** |
| **VDAG_08705** | *peroxisome assembly protein* | 9.95 | -2.65 | **-6.26** |
| **VDAG_08681** | *hypothetical protein* | 2532.28 | -2.65 | **-6.26** |
| **VDAG_03860** | *hypothetical protein* | 1298.07 | -2.64 | **-6.24** |
| **VDAG_04157** | *phenol 2-monooxygenase* | 928.83 | -2.64 | **-6.23** |
| **VDAG_01794** | *hypothetical protein* | 1411.44 | -2.63 | **-6.19** |
| **VDAG_08163** | *hypothetical protein* | 724.07 | -2.63 | **-6.19** |
| **VDAG_00159** | *hypothetical protein* | 113.39 | -2.63 | **-6.19** |
| **VDAG_02259** | *glutamate decarboxylase* | 366.62 | -2.62 | **-6.16** |
| **VDAG_02046** | *ferric reductase transmembrane component 5* | 705.98 | -2.62 | **-6.15** |
| **VDAG_03667** | *cysteine synthase B* | 405.59 | -2.62 | **-6.14** |
| **VDAG_09667** | *cytochrome c oxidase polypeptide IV* | 3599.02 | -2.62 | **-6.14** |
| **VDAG_03861** | *thiol-specific monooxygenase* | 769.08 | -2.61 | **-6.12** |
| **VDAG_02519** | *peroxiredoxin type-2* | 1904.49 | -2.61 | **-6.11** |
| **VDAG_00948** | *solute carrier family 25 member 40* | 534.81 | -2.60 | **-6.08** |
| **VDAG_02631** | *hypothetical protein* | 82.83 | -2.60 | **-6.08** |
| **VDAG_03789** | *hypothetical protein* | 560.71 | -2.60 | **-6.05** |
| **VDAG_05646** | *NADP-dependent alcohol dehydrogenase* | 456.58 | -2.59 | **-6.04** |
| **VDAG_03653** | *hypothetical protein* | 364.87 | -2.59 | **-6.03** |
| **VDAG_06435** | *hypothetical protein* | 9.76 | -2.59 | **-6.03** |
| **VDAG_08011** | *hypothetical protein* | 90.74 | -2.58 | **-6.00** |
| **VDAG_06726** | *diphthamide biosynthesis protein* | 227.56 | -2.58 | **-5.99** |
| **VDAG_05760** | *hypothetical protein* | 4.76 | -2.58 | **-5.97** |
| **VDAG_02467** | *hypothetical protein* | 96.54 | -2.58 | **-5.96** |
| **VDAG_07213** | *hypothetical protein* | 50.52 | -2.57 | **-5.94** |
| **VDAG_02568** | *hypothetical protein* | 8.86 | -2.57 | **-5.93** |
| **VDAG_10213** | *sulfite reductase flavoprotein alpha-component* | 492.57 | -2.57 | **-5.93** |
| **VDAG_09250** | *alanyl-tRNA synthetase domain-containing protein* | 1108.85 | -2.57 | **-5.93** |
| **VDAG_01258** | *aspartate aminotransferase* | 2307.20 | -2.56 | **-5.91** |
| **VDAG_10045** | *transcriptional activator protein DAL81* | 469.51 | -2.56 | **-5.91** |
| **VDAG_03226** | *tubulin-specific chaperone E* | 710.11 | -2.55 | **-5.88** |
| **VDAG_01538** | *cytochrome c oxidase assembly protein COX15* | 415.59 | -2.55 | **-5.86** |
| **VDAG_07415** | *integral membrane protein* | 67.46 | -2.55 | **-5.84** |
| **VDAG_02969** | *hypothetical protein* | 34.95 | -2.54 | **-5.84** |
| **VDAG_01728** | *hypothetical protein* | 13.82 | -2.54 | **-5.83** |
| **VDAG_04421** | *hypothetical protein* | 165.28 | -2.54 | **-5.81** |
| **VDAG_06194** | *hypothetical protein* | 1246.58 | -2.54 | **-5.81** |
| **VDAG_01708** | *phosphoribosylaminoimidazole-succinocarboxamide synthase* | 24.58 | -2.54 | **-5.81** |
| **VDAG_07951** | *hypothetical protein* | 54.50 | -2.53 | **-5.79** |
| **VDAG_05428** | *hypothetical protein* | 15.74 | -2.53 | **-5.78** |
| **VDAG_04225** | *hypothetical protein* | 19.43 | -2.53 | **-5.77** |
| **VDAG_09921** | *hypothetical protein* | 716.94 | -2.53 | **-5.77** |
| **VDAG_09491** | *phenylacetaldoxime dehydratase* | 234.35 | -2.53 | **-5.77** |
| **VDAG_03962** | *aerobactin siderophore biosynthesis protein iucB* | 89.78 | -2.53 | **-5.77** |
| **VDAG_04426** | *hypothetical protein* | 46.55 | -2.53 | **-5.77** |
| **VDAG_01582** | *riboflavin kinase* | 85.20 | -2.52 | **-5.75** |
| **VDAG_07583** | *phosphate-repressible phosphate permease* | 536.75 | -2.52 | **-5.75** |
| **VDAG_03221** | *hypothetical protein* | 36.17 | -2.52 | **-5.75** |
| **VDAG_08388** | *hypothetical protein* | 137.33 | -2.52 | **-5.74** |
| **VDAG_03083** | *integral membrane protein* | 342.78 | -2.52 | **-5.73** |
| **VDAG_02618** | *hypothetical protein* | 124.81 | -2.52 | **-5.72** |
| **VDAG_09135** | *WSC domain-containing protein* | 4.74 | -2.51 | **-5.71** |
| **VDAG_03358** | *hypothetical protein* | 220.70 | -2.51 | **-5.70** |
| **VDAG_04729** | *acetylornithine aminotransferase* | 232.77 | -2.51 | **-5.68** |
| **VDAG_00228** | *hypothetical protein* | 1083.22 | -2.50 | **-5.68** |
| **VDAG_01501** | *hypothetical protein* | 55.51 | -2.50 | **-5.67** |
| **VDAG_00574** | *3-phytase A* | 62.07 | -2.50 | **-5.66** |
| **VDAG_04914** | *transcription factor* | 9.15 | -2.50 | **-5.65** |
| **VDAG_04084** | *hypothetical protein* | 79.78 | -2.50 | **-5.64** |
| **VDAG_03318** | *nucleotide exchange factor SIL1* | 507.03 | -2.49 | **-5.63** |
| **VDAG_03110** | *hypothetical protein* | 229.13 | -2.49 | **-5.62** |
| **VDAG_02077** | *L-lactate dehydrogenase* | 557.62 | -2.48 | **-5.59** |
| **VDAG_02576** | *monothiol glutaredoxin-5* | 389.61 | -2.48 | **-5.58** |
| **VDAG_02775** | *methylcrotonoyl-CoA carboxylase subunit alpha* | 1060.78 | -2.48 | **-5.57** |
| **VDAG_06759** | *hypothetical protein* | 142.60 | -2.47 | **-5.55** |
| **VDAG_07888** | *hypothetical protein* | 605.99 | -2.47 | **-5.55** |
| **VDAG_05792** | *peroxisomal catalase* | 858.85 | -2.47 | **-5.53** |
| **VDAG_05699** | *hypothetical protein* | 35.39 | -2.47 | **-5.53** |
| **VDAG_10486** | *cytochrome P450 monooxygenase* | 81.61 | -2.47 | **-5.53** |
| **VDAG_04011** | *methylglutaconyl-CoA hydratase* | 339.50 | -2.46 | **-5.51** |
| **VDAG_03467** | *hypothetical protein* | 979.04 | -2.46 | **-5.50** |
| **VDAG_03863** | *calcium-transporting ATPase* | 523.23 | -2.46 | **-5.50** |
| **VDAG_04629** | *hypothetical protein* | 14.13 | -2.46 | **-5.49** |
| **VDAG_03584** | *hypothetical protein* | 135.46 | -2.45 | **-5.48** |
| **VDAG_07238** | *pectin lyase* | 134.99 | -2.45 | **-5.45** |
| **VDAG_03820** | *carbonic anhydrase* | 118.25 | -2.43 | **-5.41** |
| **VDAG_09919** | *iron transport multicopper oxidase FET3* | 1089.84 | -2.43 | **-5.38** |
| **VDAG_02483** | *COQ4* | 299.75 | -2.43 | **-5.38** |
| **VDAG_00795** | *zinc carboxypeptidase A 1* | 23.37 | -2.42 | **-5.37** |
| **VDAG_10079** | *hypothetical protein* | 32.93 | -2.42 | **-5.36** |
| **VDAG_00814** | *hypothetical protein* | 8.40 | -2.42 | **-5.35** |
| **VDAG_02856** | *DNA mismatch repair protein mutS* | 87.37 | -2.42 | **-5.34** |
| **VDAG_07553** | *NAD dependent epimerase/dehydratase family protein* | 245.05 | -2.42 | **-5.34** |
| **VDAG_05230** | *hypothetical protein* | 7.87 | -2.42 | **-5.34** |
| **VDAG_03222** | *inorganic phosphate transporter* | 890.92 | -2.42 | **-5.34** |
| **VDAG_08589** | *ketol-acid reductoisomerase* | 2565.98 | -2.41 | **-5.33** |
| **VDAG_02457** | *sulfate adenylyltransferase* | 596.56 | -2.41 | **-5.31** |
| **VDAG_02041** | *aspartyl-tRNA synthetase* | 333.72 | -2.41 | **-5.31** |
| **VDAG_07347** | *vip1* | 1280.35 | -2.41 | **-5.30** |
| **VDAG_07065** | *hypothetical protein* | 170.20 | -2.40 | **-5.29** |
| **VDAG_08549** | *mitogen-activated protein kinase MAF1* | 937.88 | -2.40 | **-5.29** |
| **VDAG_01339** | *hypothetical protein* | 918.87 | -2.40 | **-5.27** |
| **VDAG_09593** | *choline dehydrogenase* | 847.35 | -2.40 | **-5.27** |
| **VDAG_09678** | *hypothetical protein* | 28.44 | -2.40 | **-5.27** |
| **VDAG_04029** | *hypothetical protein* | 255.18 | -2.40 | **-5.26** |
| **VDAG_07116** | *aromatic and neutral aliphatic amino acid permease* | 180.51 | -2.39 | **-5.25** |
| **VDAG_09520** | *hypothetical protein* | 215.01 | -2.39 | **-5.23** |
| **VDAG_05970** | *hypothetical protein* | 482.61 | -2.38 | **-5.22** |
| **VDAG_08781** | *hypothetical protein* | 67.12 | -2.38 | **-5.22** |
| **VDAG_09114** | *galactose oxidase* | 47.49 | -2.38 | **-5.21** |
| **VDAG_09316** | *hypothetical protein* | 293.96 | -2.38 | **-5.20** |
| **VDAG_01989** | *hydroxymethylglutaryl-CoA lyase* | 1115.88 | -2.38 | **-5.20** |
| **VDAG_01869** | *taurine catabolism dioxygenase TauD* | 101.13 | -2.38 | **-5.20** |
| **VDAG_01727** | *molybdenum cofactor synthesis protein 2 large subunit* | 63.20 | -2.38 | **-5.19** |
| **VDAG_02003** | *hypothetical protein* | 1312.01 | -2.37 | **-5.18** |
| **VDAG_00121** | *hypothetical protein* | 24.18 | -2.37 | **-5.18** |
| **VDAG_10015** | *1,4-alpha-glucan-branching enzyme* | 1399.08 | -2.37 | **-5.17** |
| **VDAG_08249** | *sulfate transporter 4.1* | 533.18 | -2.37 | **-5.17** |
| **VDAG_09804** | *alpha/beta hydrolase fold-3 domain-containing protein* | 57.98 | -2.36 | **-5.15** |
| **VDAG_08272** | *mitochondrial phosphate carrier protein* | 290.08 | -2.36 | **-5.14** |
| **VDAG_07610** | *hypothetical protein* | 1183.75 | -2.36 | **-5.13** |
| **VDAG_04049** | *CAP20 protein* | 1132.06 | -2.36 | **-5.12** |
| **VDAG_08045** | *hypothetical protein* | 590.82 | -2.36 | **-5.12** |
| **VDAG_07578** | *FAD binding domain-containing protein* | 72.18 | -2.35 | **-5.11** |
| **VDAG_08400** | *GTPase-activating protein GYP7* | 844.13 | -2.35 | **-5.11** |
| **VDAG_06561** | *hypothetical protein* | 210.60 | -2.35 | **-5.10** |
| **VDAG_01655** | *rhamnolipids biosynthesis 3-oxoacyl-[acyl-carrier-protein] reductase* | 38.73 | -2.35 | **-5.09** |
| **VDAG_04515** | *hypothetical protein* | 22.17 | -2.34 | **-5.07** |
| **VDAG_08511** | *hypothetical protein* | 928.77 | -2.34 | **-5.06** |
| **VDAG_05671** | *hypothetical protein* | 69.62 | -2.34 | **-5.06** |
| **VDAG_01898** | *hypothetical protein* | 205.27 | -2.33 | **-5.03** |
| **VDAG_09856** | *hypothetical protein* | 278.55 | -2.33 | **-5.02** |
| **VDAG_08672** | *hypothetical protein* | 23.62 | -2.32 | **-5.00** |

**Table S5.** Primers used for qPCR analysis.

| **Gene** | **Primer** | **Sequence** |
| --- | --- | --- |
| ***RPS18B***  (AT1G34030) | forward | 5’-GTC TCC AAT GCC CTT GAC AT-3’ |
|  | reverse | 5’-TCT TTC CTC TGC GAC CAG TT-3’ |
| ***WAKL10***  (AT1G79680) | forward | 5’-AGG GAA GGA AAC GAC CAA GT-3’ |
|  | reverse | 5’-GCG ACG AAG ATG TTG TAG CA-3’ |
| ***SWEET11***  (AT3G48740) | forward | 5’-AGG CAC AGT TTC ATC CCC TG-3’ |
|  | reverse | 5’-TGC TTG CCA TGT TTA GGG GT-3’ |
| ***SWEET3***  (AT5G53190) | forward | 5’-GTC GGC ATC CTT CTC GAA TCT-3’ |
|  | reverse | 5’-CTG TCG TTA AGC CGA ACC CA-3’ |
| ***Actin2_VD***  (VDAG_07506) | forward | 5’-CGT TCA GGT CAT CAC GCA C-3’ |
|  | reverse | 5’-TAT TCT TGG TAC TCC GCC TTG-3’ |
| ***VD - Gluc. transp.***  (VDAG_02979) | forward | 5’-ATC ACG TCC GCC ATC AAT GT-3’ |
|  | reverse | 5’-GAC GAA ACT GTG CCC GAA AC-3’ |
| ***VD - PARN***  (VDAG_06565) | forward | 5’-TTC CAG GAC ACC AGA GAC GA-3’ |
|  | reverse | 5’-TTC AAA GCC GTT GAG GGT CA-3’ |
