# Supplementary material for: Verticillium dahliae-Arabidopsis Interaction Causes Changes in Gene Expression Profiles and Jasmonate Levels on Different Time Scales
Source: Front Microbiol. 2018 Feb 13;9:217. doi: 10.3389/fmicb.2018.00217 (PMC5819561; doi:10.3389/fmicb.2018.00217)
Supplement: Supplementary file 2 [file DataSheet1.pdf]

Supplemental Material

Figure S1

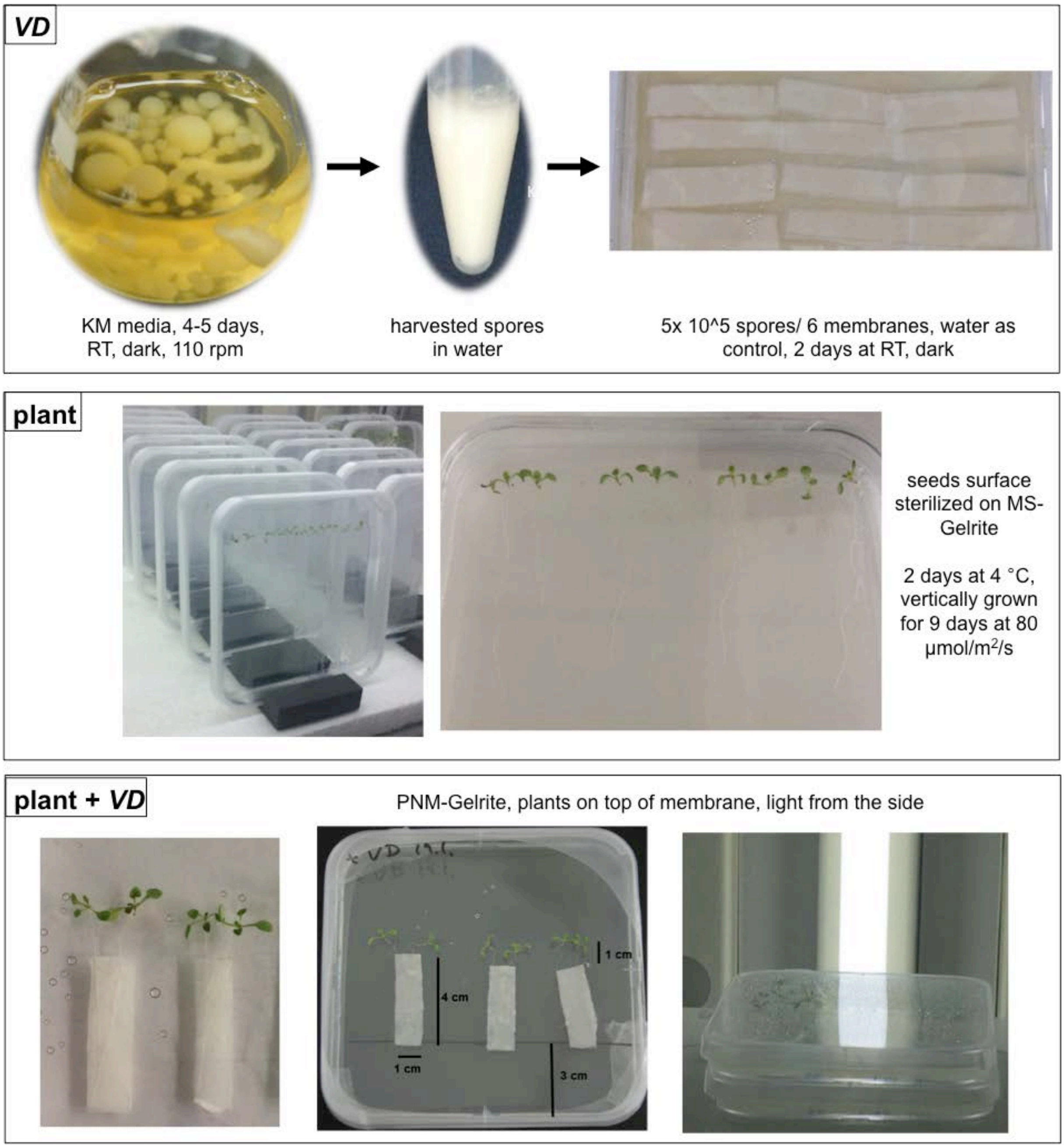

Figure S1. Workflow for Co-cultivation of *Arabidopsis thaliana* and *Verticillium dahliae* for RNA-seq analysis. Shown are the growth of the fungus (*VD*, upper lane), the seedlings (plant, middle) and the co-cultivation (plant + *VD*, lower lane).

Figure S2

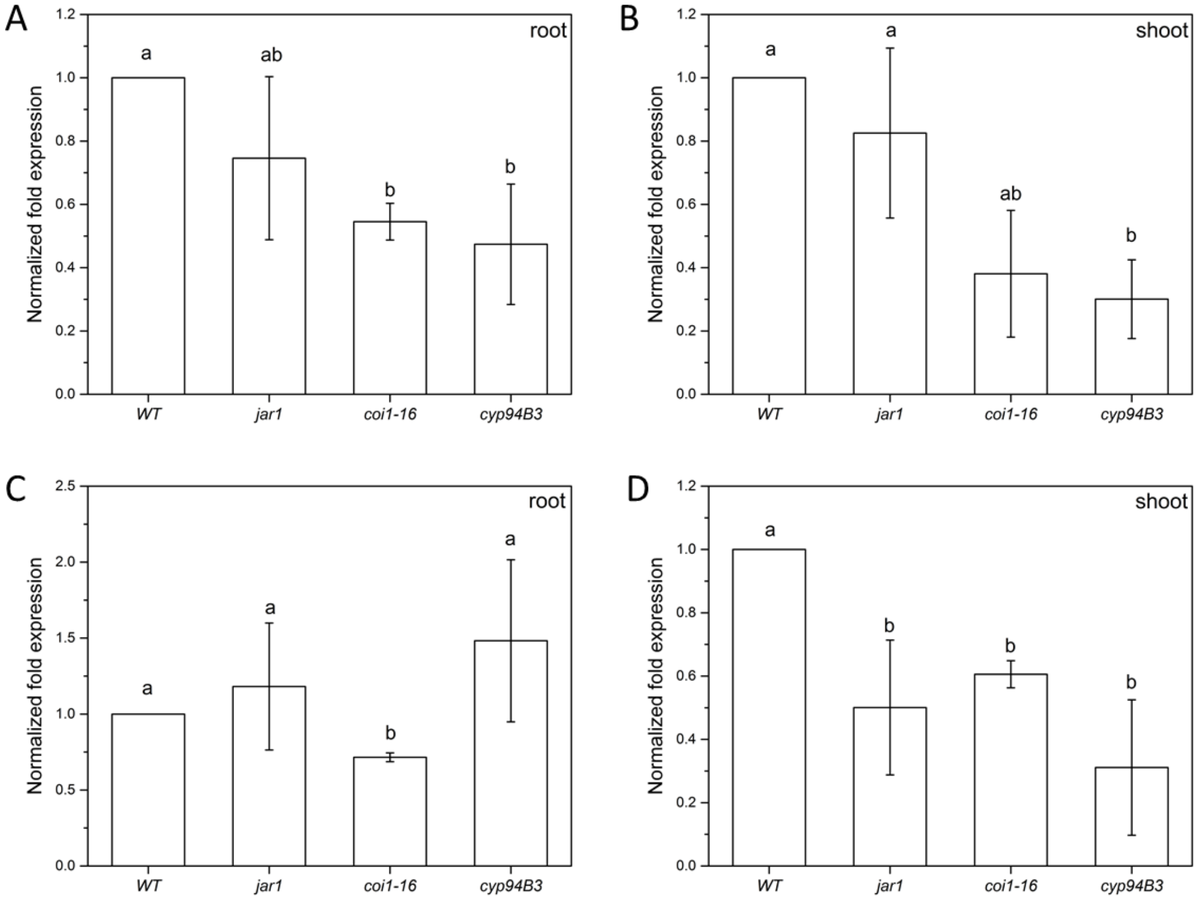

Figure S2. Colonization of WT and different *Arabidopsis* mutant plants by *V. dahliae* 10 and *20 dpi*. Shown is the normalized fold expression (± SE, n=3) of *VD_Actin2* 10 (**A, B**) and 20 (**C**, **D**) *dpi* in *VD*-infected WT, *jar1*, *coi1-16* and *cyp94B3* plants. The expression level of *VD_Actin2* in *VD*-infected WT plants was used as control and set to 1.0. The mRNA levels for each cDNA probe were normalized with respect to the *RPS18B* mRNA level. Statistically significant differences between the mutants were analyzed by one-way ANOVA, $p<0.05$ (Sidak). Different letters indicate a statistically significant difference.
